# Copper(II)-Catalyzed Selective C_Ar_-H Bond Formylation: Synthesis of Dialdehyde Aniline

**DOI:** 10.3389/fchem.2022.891858

**Published:** 2022-05-24

**Authors:** Shiwei Guo, Yinghua Li, Weibin Fan, Zhiqi Liu, Deguang Huang

**Affiliations:** ^1^ State Key Laboratory of Structural Chemistry, Fujian Institute of Research on the Structure of Matter, Chinese Academy of Sciences, Fuzhou, China; ^2^ College of Materials Science and Opto Electronic Technology, University of Chinese Academy of Sciences, Beijing, China

**Keywords:** formylation, C-H bond activation, copper catalyzed, Selectfluor, radical, carbon and oxygen sources

## Abstract

A simple and efficient method for the synthesis of dialdehyde aniline in good yields (up to 83%) is explored using Cu(OTf)_2_ as the catalyst, Selectfluor as the radical initiator, and DMSO as both the carbon and oxygen sources. Experimental studies indicate that the reaction is achieved by the formylation of two C_Ar_-H bonds, first at the para-position and then at the ortho-position. A possible mechanism is proposed, including the thermal homolysis of Selectfluor, the Cu(II)-facilitated formylation of the C_Ar_-H bonds, and the hydrolysis of the amide under alkaline conditions in air atmosphere.

## Introduction

**SCHEME 1:**
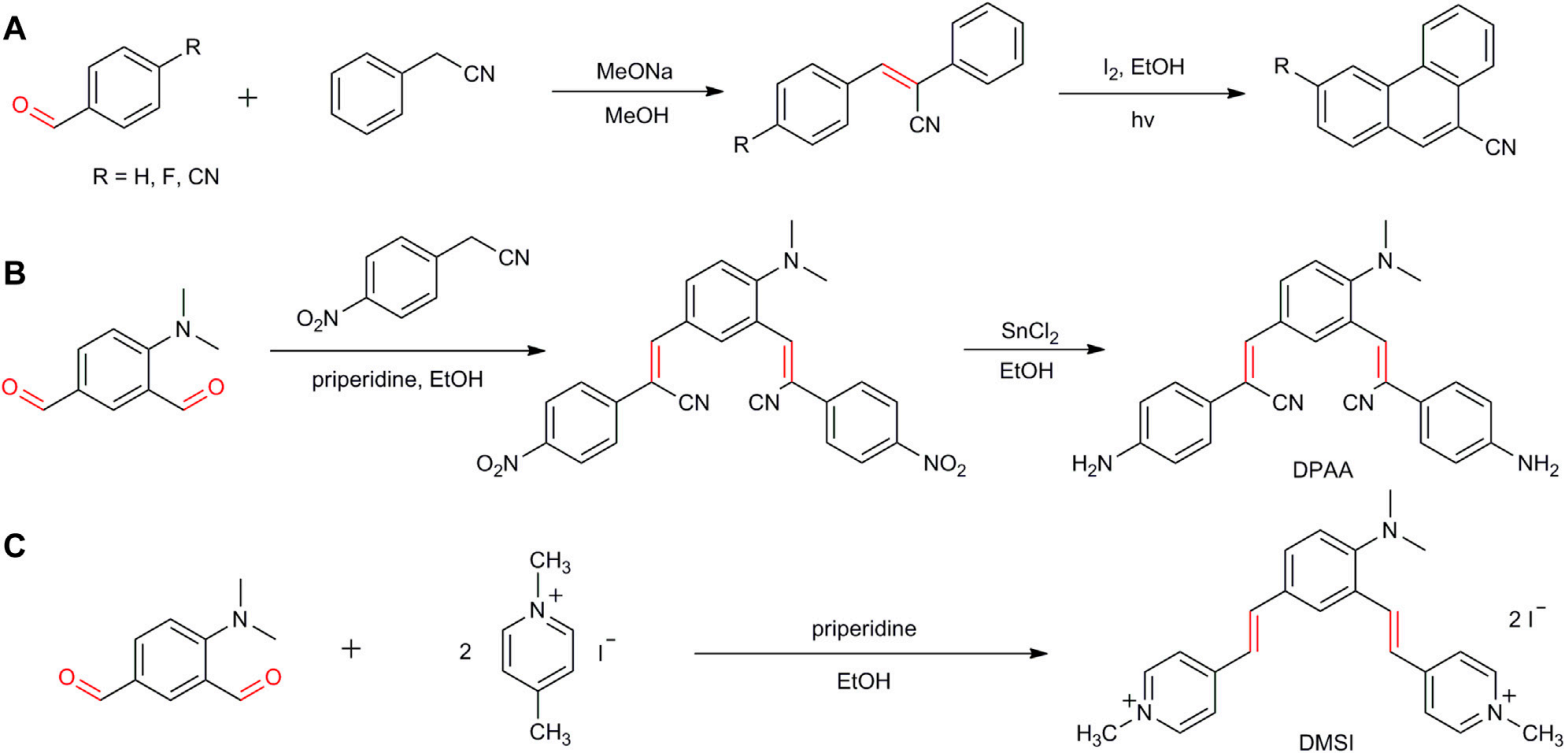
Pharmaceuticals and functional materials synthesized by aromatic aldehydes.

Aromatic aldehydes are valuable synthetic intermediates in the fields of pharmaceuticals, agrochemicals, pesticides, and chemical sciences. The formyl groups display good stability and exhibit high activity toward coupling reactions with the formation of C–X (X = C, N, S, etc.) bonds (Dirksen and Dawson., 2008; Waddell and Mack., 2009; Ambreen and Wirth., 2014; Liang et al., 2017; Tamang and Findlater., 2017). A couple of reactions have been developed for the synthesis of pharmaceuticals and functional materials. For example, phenanthrene-9-carbonitrile and its derivatives, a kind of anticancer drugs, were synthesized by the reaction of 4-substituted benzaldehydes with phenylacetonitrile in MeOH under alkaline conditions, followed by the coupling reaction for forming the C_Ar_-C_Ar_ bond (Scheme 1A) (Perin et al., 2020). (2Z, 2′Z)-3,3'-[4-(dimethylamino)-1,3-phenylene]bis[2-(4-aminophenyl)acrylonitrile] (DPAA), a stimuli-responsive organic fluorescent material, was obtained by the condensation of 4-(dimethylamino)isophthalaldehyde with *p*-nitrophenylacetonitrile in EtOH with the reduction of the nitro groups to the amine groups (Scheme 1B) (Fang et al., 2018). Trans-4-[4-(dimethylamino)styryl]-1-methylpyridinium iodide (DSMI), a cationic hemicyanine dye, commonly used as a fluorescent probe for DNA analysis, was also synthesized by the reaction of 4-(dimethylamino)isophthalaldehyde with 1,4-dimethylpyrinium iodide under reflux conditions (Scheme 1C) (Sun et al., 2014).

**SCHEME 2:**
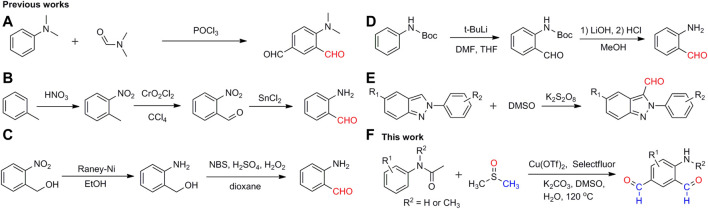
Different strategies to synthesize aromatic aldehydes.

To date, a number of methodologies have been developed for the synthesis of aromatic aldehydes. One of the most efficient methods is the direct installation of formyl groups on the aromatic rings. For example, the reaction of N,N-dimethylaniline with phosphorus oxychloride using N, N-dimethylformamide (DMF) as the formyl source yielded the dialdehyde compound (dimethylamino)isophthalaldehyde, in which the amino group was protected by two methyl groups (Scheme 2A) (Fang et al., 2018; Sun et al., 2014). It will be more difficult to synthesize aminobenzaldehydes in one step. A common practice is to generate the amino group and formyl group in different reactions, including the oxidation of the methyl group to the formyl group first and then the reduction of the nitro group to the amino group (Scheme 2B) (Wheeler., 1958; Kijima et al., 1984; Dai et al., 2011), the reduction of the nitro group to the amino group first and then the oxidation of the alcohol group to the formyl group (Scheme 2C) (Sorkin and Hinden., 1949; Liu et al., 2015), and the addition of the formyl group to the BOC-protected aniline first and then the deprotection of the protected amino group (Scheme 2D) (Ma et al., 2002). In addition, a couple of small molecules have been studied as the formyl source for the construction of heterocyclic aromatic aldehydes, such as dimethyl sulfoxide (Scheme 2E) (Zhang et al., 2014; Suvam et al., 2021), N, N-dimethylformamide (Koeller and Lellouche., 1999; Tang and Shi., 2008; Khadka et al., 2012; Badalyan et al., 2014; Popov et al., 2019), and carbon monoxide (Schoenbe et al., 1974; Klaus et al., 2005; Sergeev et al., 2008). All these methods have their limitations for the production of aminobenzaldehydes, such as the rigorous reaction conditions, strong reducing agents, multistep processes, and/or low yields. A simple and efficient method is required for the synthesis of aminobenzaldehydes in one-step, consistent with the development of aldehyde–amine chemistry and its related pharmacological chemistry.

Selectfluor is an efficient oxidant and/or radical initiator in organic synthesis (Galloway et al., 2017; Gao et al., 2018; Hu et al., 2019; Niu et al., 2019). We have used it as an oxidant for the generation of methylene groups *via* C–N/C–O bond break (Jin et al., 2021; Zhang et al., 2022). Herein, we report a facile and efficient method for the preparation of 4-aminoisophthalaldehyde, in which DMSO, Cu(OTf)_2_, and Selectfluor were used as the formyl source, catalyst and radical initiator, respectively Scheme 2F.

## Materials and Methods

### General Information

The single-crystal data of compounds were collected by a Cu–/Mo–Kα rotating anode source, using a Supernova diffractometer with the ω-scan method. ESI-MS was performed using a Bruker Impact II quardrupole time-of-flight mass spectrometer. ^1^H NMR and ^13^C NMR spectra were recorded on a Bruker Avance III (400 MHz) and JNM ECS400S (400 MHz), respectively. Chemical shifts are expressed in δ ppm values with reference to tetramethylsilane (TMS) as the internal standard. NMR multiplicities are abbreviated as follows: s = singlet; d = doublet; m = multiplet. Coupling constants (J) are expressed in Hz. Volume reduction and drying steps were performed *in vacuo*. All the reagents were purchased from commercial sources and used as received. Dimethyl sulfoxide and dichloromethane were freshly distilled over CaH_2_. Tetrahydrofuran, diethyl ether, toluene, and n-hexane were distilled over sodium under N_2_. The starting material acetanilides were synthesized according to the procedure given by Ofori et al., (2021).

### General Procedure for the Synthesis of Starting Materials

A mixture of aniline derivatives (4.0 mmol) and triethylamine (4.4 mmol) in dry CH_2_Cl_2_ (50 mL) was added to acetyl chloride (4.4 mmol) dropwise while stirring. After addition, the mixture was allowed to stir for 4 h at room temperature. The reaction was quenched by adding water (20 mL). The organic layer was washed with water (2 × 20 mL), dried over Na_2_SO_4_, and concentrated *in vacuo* to yield products as solid.

### General Procedure for Compounds 3a–3x

A mixture of acetanilides **1** (0.2 mmol), Cu(OTf)_2_ (0.04 mmol), Selectfluor (0.5 mmol), K_2_CO_3_ (0.4 mmol), and H_2_O (50 μL) in DMSO (1 mL) was stirred at 120°C for 12 h in air. After cooling to room temperature, the reaction mixture was diluted with CH_2_Cl_2_ (20 mL) and filtered through a pad of silica gel. The organic solution was washed with brine (2 × 30 mL). The aqueous solution was extracted with CH_2_Cl_2_ (3 × 30 mL). The organic layers were combined and dried over anhydrous Na_2_SO_4_ overnight. The solvent was removed under reduced pressure. The crude product was purified on a silica gel column eluted with petroleum ether/ethyl acetate (3:1 to 2:1 v/v) to yield the products **3a–3x**.

### General Procedure for Mechanistic Exploration

1) A mixture of N-(4-formylphenyl)acetamide **1y** (0.2 mmol), Cu(OTf)_2_ (0.04 mmol), Selectfluor (0.5 mmol), K_2_CO_3_ (0.4 mmol), and H_2_O (50 μL) in DMSO (1 mL) was stirred at 120°C for 12 h in air. After cooling to room temperature, the reaction mixture was diluted with CH_2_Cl_2_ (20 mL) and filtered through a pad of silica gel. The organic solution was washed with brine (2 × 30 mL), and the aqueous solution was extracted with CH_2_Cl_2_ (3 × 30 mL). The organic layers were combined and dried over anhydrous Na_2_SO_4_ overnight. The solvent was removed under reduced pressure. The crude product was purified on a silica gel column eluted with petroleum ether/ethyl acetate (2:1 v/v) to afford the product **3a** in 81% yield (23.8 mg).
2) A mixture of N-(2-formylphenyl)acetamide **1z** (0.2 mmol), Cu(OTf)_2_ (0.04 mmol), Selectfluor (0.5 mmol), K_2_CO_3_ (0.4 mmol), and H_2_O (50 μL) in DMSO (1 mL) was stirred at 120°C for 12 h in air. After cooling to room temperature, the reaction mixture was diluted with CH_2_Cl_2_ (20 mL) and filtered through a pad of silica gel. No signal of the product **3a** was observed on the TLC plate.
3) A mixture of acetanilide **1a** (0.2 mmol), Cu(OTf)_2_ (0.04 mmol), Selectfluor (0.5 mmol), K_2_CO_3_ (0.4 mmol), and H_2_O (50 μL) in DMSO-d_6_ (1 mL) was stirred at 120°C for 12 h in air. After cooling to room temperature, the reaction mixture was diluted with CH_2_Cl_2_ (20 mL) and filtered through a pad of silica gel. The organic solution was washed with brine (2 × 30 mL), and the aqueous solution was extracted with CH_2_Cl_2_ (3 × 30 mL). The organic layers were combined and dried over anhydrous Na_2_SO_4_ overnight. The solvent was removed under reduced pressure. The crude product was purified on a silica gel column eluted with petroleum ether/ethyl acetate (2:1 v/v) to afford the product **3a′** (76%, 22.2 mg) with 49% and 45% deuteration of the two formyl groups, respectively.
4) A mixture of acetanilide **1a** (0.2 mmol), Cu(OTf)_2_ (0.04 mmol), Selectfluor (0.5 mmol), K_2_CO_3_ (0.4 mmol), and H_2_O (50 μL) in DMSO (1 mL) was stirred at 120°C for 12 h in N_2_. After cooling to room temperature, the reaction mixture was diluted in dichloromethane (20 mL) and filtered through a pad of silica gel. The organic solution was washed with brine (2 × 30 mL), and the aqueous solution was extracted with CH_2_Cl_2_ (3 × 30 mL). The organic layers were combined and dried over anhydrous Na_2_SO_4_ overnight. The solvent was removed under reduced pressure. The crude product was purified on a silica gel column eluted with petroleum ether/ethyl acetate (2:1 v/v) to afford the product **3a** in 79% yield (23.3 mg).
5) A mixture of acetanilide **1a** (0.2 mmol), Cu(OTf)_2_ (0.04 mmol), Selectfluor (0.5 mmol), K_2_CO_3_ (0.4 mmol), and H_2_^18^O (50 μL) in DMSO (1 mL) was stirred at 120°C for 12 h in air. After cooling to room temperature, the reaction mixture was diluted with dichloromethane (20 mL) and filtered through a pad of silica gel. The organic solution was washed with brine (2 × 30 mL), and the aqueous solution was extracted with CH_2_Cl_2_ (3 × 30 mL). The organic layers were combined and dried over anhydrous Na_2_SO_4_ overnight. The organic layers were combined, and the solvent was removed under reduced pressure. The crude product was purified on a silica gel column eluted with petroleum ether/ethyl acetate (2:1 v/v) to afford the product **3a** in 79% yield (23.5 mg) rather than **3a”** [(^18^O)-3a].
6) A mixture of acetanilide **1a** (0.2 mmol), Cu(OTf)_2_ (0.04 mmol), Selectfluor (0.5 mmol), K_2_CO_3_ (0.4 mmol), and H_2_O (50 μL) in DMSO (1 mL) was stirred at 120°C for 12 h in air. After cooling to room temperature, the reaction mixture was diluted with dichloromethane (20 mL) and filtered through a pad of silica gel. The organic solution was washed with brine (2 × 30 mL), and the aqueous solution was extracted with CH_2_Cl_2_ (3 × 30 mL). The organic layers were combined and dried over anhydrous Na_2_SO_4_ overnight. The solvent was removed under reduced pressure. The crude product was purified on a silica gel column eluted with petroleum ether/ethyl acetate (1:1 v/v) to afford the product **4a** in 78% yield (38.7 mg).
7) A mixture of acetanilide **1a** (0.2 mmol), Cu(OTf)_2_ (0.04 mmol), Selectfluor (0.5 mmol), K_2_CO_3_ (0.4 mmol), 2,2,6,6-tetramethylpiperidine-1-oxyl (TEMPO, 1 mmol) [or butylated hydroxytoluene (BHT, 1 mmol)], and H_2_O (50 μL) in DMSO (1 mL) was stirred at 120°C for 12 h in air. After cooling to room temperature, the reaction mixture was diluted with dichloromethane (20 mL) and filtered through a pad of silica gel. No signal of the product **3a** was observed on the TLC plate.

**NMR DATA** (Note: The NH_2_ protons of all the compounds could not be detected in ^1^H NMR spectra).

### 4-Aminoisophthalaldehyde (3a)

Yield, 79% (23.5 mg); yellow solid; melting point, 110–112°C; ^1^H NMR (400 MHz, CDCl_3_) δ 9.96 (s, 1H), 9.81 (s, 1H), 8.05 (d, J = 1.8 Hz, 1H), 7.86 (dd, J = 8.7, 1.8 Hz, 1H), and 6.76 (d, J = 8.7 Hz, 1H). ^13^C NMR (100 MHz, CDCl_3_) δ 193.4 (s, 1C), 189.3 (s, 1C), 154.2 (s, 1C), 140.5 (s, 1C), 134.8 (s, 1C), 126.2 (s, 1C), 117.9 (s, 1C), and 116.6 (s, 1C). HRMS m/z (ESI) [M + Na]^+^ calculated for C_8_H_7_NO_2_Na, 172.0374; found, 172.0369. IR neat: 3,434, 2,968, 2,926, 1,656, and 1,616 cm^−1^.

### 4-Amino-5-Methoxyisophthalaldehyde (3b)

Yield, 83% (29.7 mg); light yellow solid; melting point, 125–127°C; ^1^H NMR (400 MHz, CDCl_3_) δ 9.95 (s, 1H), 9.78 (s, 1H), 7.66 (d, J = 1.5 Hz, 1H), 7.38 (s, 1H), and 3.96 (s, 3H). ^13^C NMR (101 MHz, CDCl_3_) δ 193.0 (s, 1C), 189.6 (s, 1C), 147.0 (s, 1C), 146.3 (s, 1C), 134.4 (s, 1C), 125.3 (s, 1C), 116.6 (s, 1C), 109.2 (s, 1C), and 56.1 (s, 1C). HRMS m/z (ESI) [M + Na]^+^ calculated for C_9_H_9_NO_3_Na, 202.0480; found, 202.0475. IR neat: 3,406, 2,812, 2,749 1,631, and 1,593 cm^−1^.

### 4-Amino-5-(Methylthio)Isophthalaldehyde (3c)

Yield, 80%, (31.2 mg); light yellow solid melting point, 123–125°C, ^1^H NMR (400 MHz, CDCl_3_) δ 9.95 (s, 1H), 9.80 (s, 1H), 8.09 (d, J = 1.8 Hz, 1H), 7.98 (d, J = 1.8 Hz, 1H), and 2.41 (s, 3H). ^13^C NMR (101 MHz, CDCl_3_) δ 193.2 (s, 1C), 189.0 (s, 1C), 153.7 (s, 1C), 139.8 (s, 1C), 138.3 (s, 1C), 125.9 (s, 1C), 123.1 (s, 1C), 117.5 (s, 1C), and 17.9 (s, 1C). HRMS m/z (ESI) [M + Na]^+^ calculated for C_9_H_9_NO_2_SNa 218.0252; found, 218.0247. IR neat: 3,422, 2,933, 2,926, 1,654, and 1,590 cm^−1^.

### 4-Amino-5-Phenoxyisophthalaldehyde (3d)

Yield, 82% (39.5 mg); light yellow solid; melting point, 135–137°C; ^1^H NMR (400 MHz, CDCl_3_) δ 10.00 (s, 1H), 9.74 (s, 1H), 7.82 (d, J = 1.6 Hz, 1H), 7.43–7.36 (m, 3H), 7.20 (t, J = 7.4 Hz, 1H), and 7.10–7.03 (m, 1H). ^13^C NMR (101 MHz, CDCl_3_) δ 193.0 (s, 1C), 189.1 (s, 1C), 155.3 (s, 1C), 146.9 (s, 1C), 145.2 (s, 1C), 135.3 (s, 1C), 130.3 (s, 2C), 125.3 (s, 1C), 124.8 (s, 1C), 119.2 (s, 2C), 118.3 (s, 1C), and 118.2 (s, 1C). HRMS m/z (ESI) [M + Na]^+^ calculated for C_14_H_11_NO_3_Na, 264.0637; found, 264.0632. IR neat: 3,412, 2,915, 2,853, 1,628, and 1,588 cm^−1^.

### 4-Amino-5-(Benzyloxy)isophthalaldehyde (3e)

Yield, 81% (41.3 mg); yellow solid; melting point, 142–144°C; ^1^H NMR (400 MHz, CDCl_3_) δ 9.96 (s, 1H), 9.78 (s, 1H), 7.68 (d, J = 1.4 Hz, 1H), 7.47 (d, J = 1.0 Hz, 1H), 7.45–7.41 (m, 3H), 7.40 (t, J = 2.4 Hz, 1H), and 7.39–7.35 (m, 1H), 5.16 (s, 2H). ^13^C NMR (101 MHz, CDCl_3_) δ 193.0 (s, 1C), 189.5 (s, 1C), 146.4 (s, 1C), 146.1 (s, 1C), 135.6 (s, 1C), 134.5 (s, 1C), 128.9 (s, 2C), 128.7 (s, 1C), 128.0 (s, 2C), 125.3 (s, 1C), 116.8 (s, 1C), 110.6 (s, 1C), and 71.0 (s, 1C). HRMS m/z (ESI) [M + Na]^+^ calculated for C_15_H_13_NO_3_Na, 278.0793; found, 278.0788. IR neat 3,395, 2,979, 2,916, 1,656, and 1,593 cm^−1^.

### 4-Amino-5-Isopropylisophthalaldehyde (3f)

Yield, 81% (30.9 mg); yellow solid; melting point, 106–108°C, ^1^H NMR (400 MHz, CDCl_3_) δ 9.98 (s, 1H), 9.84 (s, 1H), 7.93 (s, 1H), 7.88 (s, 1H), 2.90 (dt, J = 13.5, 6.7 Hz, 1H), and 1.34 (d, J = 6.8 Hz, 6H). ^13^C NMR (101 MHz, CDCl_3_) δ 193.8 (s, 1C), 189.8 (s, 1C), 152.1 (s, 1C), 139.2 (s, 1C), 133.5 (s, 1C), 130.1 (s, 1C), 125.8 (s, 1C), 117.7 (s, 1C), 26.8 (s, 1C), and 21.7 (s, 2C). HRMS m/z (ESI) [M + Na]^+^ calculated for C_11_H_13_NO_2_Na, 214.0844; found, 214.0839. IR neat: 3,411, 2,965, 2,905, 1,646, and 1,599 cm^−1^.

### 4-Amino-5-Ethylisophthalaldehyde (3g)

Yield, 81% (28.3 mg); light yellow solid; melting point, 113–115°C, ^1^H NMR (400 MHz, CDCl_3_) δ 9.98 (s, 1H), 9.81 (s, 1H), 7.92 (d, J = 1.8 Hz, 1H), 7.80 (d, J = 0.8 Hz, 1H), 2.55 (q, J = 7.4 Hz, 2H), and 1.33 (t, J = 7.5 Hz, 3H). ^13^C NMR (101 MHz, CDCl_3_) δ 193.8 (s, 1C), 189.8 (s, 1C), 152.2 (s, 1C), 139.2 (s, 1C), 133.5 (s, 1C), 130.1 (s, 1C), 125.8 (s, 1C), 117.7 (s, 1C), 26.8 (s, 1C), and 21.7 (s, 1C). HRMS m/z (ESI) [M + Na]^+^ calculated for C_10_H_11_NO_2_Na, 200.0688; found, 200.0683. IR neat: 3,499, 2,988, 2,937, 1,656, and 1,593 cm^−1^.

### 4-Amino-5-Methylisophthalaldehyde (3h)

Yield, 81% (26.2 mg); light yellow solid; melting point, 100–102°C, ^1^H NMR (400 MHz, CDCl_3_) δ 9.97 (s, 1H), 9.81 (s, 1H), 7.94 (t, J = 3.3 Hz, 1H), 7.79 (d, J = 5.5 Hz, 1H), and 2.26 (s, 3H). ^13^C NMR (101 MHz, CDCl_3_) δ 193.6 (s, 1C), 189.6 (s, 1C), 153.1 (s, 1C), 139.1 (s, 1C), 134.5 (s, 1C), 125.7 (s, 1C), 123.4 (s, 1C), 117.3 (s, 1C), and 16.5 (s, 1C). HRMS m/z (ESI) [M + Na]^+^ calculated for C_9_H_9_NO_2_Na, 186.0531; found, 186.0526. IR neat: 3,396, 2,925, 2,835, 1,664, and 1,597 cm^−1^.

### 2-Amino-[1,1′-Biphenyl]-3,5-Dicarbaldehyde (3i)

Yield, 80% (36.1 mg); yellow solid; melting point, 136–138°C, ^1^H NMR (400 MHz, CDCl_3_) δ 10.03 (s, 1H), 9.87 (s, 1H), 8.08 (d, J = 1.9 Hz, 1H), 7.83 (d, J = 1.9 Hz, 1H), 7.54 (dd, J = 7.9, 6.5 Hz, 2H), 7.50–7.46 (m, 1H), and 7.46–7.40 (m, 2H). ^13^C NMR (101 MHz, CDCl_3_) δ 193.5 (s, 1C), 189.4 (s, 1C), 152.0 (s, 1C), 139.7 (s, 1C), 136.0 (s, 1C), 135.2 (s, 1C), 129.5 (s, 2C), 129.2 (s, 1C), 129.0 (s, 2C), 128.7 (s, 1C), 125.8 (s, 1C), and 118.0 (s, 1C). HRMS m/z (ESI) [M + Na]^+^ calculated for C_14_H_11_NO_2_Na, 248.0688; found, 248.0683. IR neat: 3,463, 2,833, 2,749, 1,664, and 1,597 cm^−1^.

### 4-Amino-5-Bromoisophthalaldehyde (3j)

Yield, 68% (31.0 mg); light yellow solid; melting point, 167–169°C, ^1^H NMR (400 MHz, CDCl_3_) δ 9.93 (s, 1H), 9.81 (s, 1H), 8.19 (d, J = 1.7 Hz, 1H), and 8.05 (d, J = 1.6 Hz, 1H). ^13^C NMR (101 MHz, CDCl_3_) δ 192.6 (s, 1C), 188.1 (s, 1C), 151.0 (s, 1C), 138.8 (s, 1C), 137.4 (s, 1C), 126.5 (s, 1C), 118.4 (s, 1C), and 110.9 (s, 1C). HRMS m/z (ESI) [M + Na]^+^ calculated for C_8_H_6_BrNO_2_Na, 249.9480; found, 249.9475. IR neat: 3,467, 2,915, 2,853 1,656, and 1,572 cm^−1^.

### 4-Amino-5-Chloroisophthalaldehyde (3k)

Yield, 63% (23.1 mg); light yellow solid; melting point, 144–146°C, ^1^H NMR (400 MHz, CDCl_3_) δ 9.98 (s, 1H), 9.82 (s, 1H), and 8.02 (s, 2H). ^13^C NMR (101 MHz, CDCl_3_) δ 192.7 (s, 1C), 188.3 (s, 1C), 150.1 (s, 1C), 138.1 (s, 1C), 133.8 (s, 1C), 125.9 (s, 1C), 120.7 (s, 1C), and 118.4 (s, 1C). HRMS m/z (ESI) [M + Na]^+^ calculated for C_8_H_6_ClNO_2_Na, 205.9985; found, 205.9980. IR neat: 3,436, 2,843, 2,770, 1,656, and 1,603 cm^−1^.

### 4-Amino-5-Fluoroisophthalaldehyde (3l)

Yield, 54% (18.1 mg); yellow solid; melting point, 152–154°C, ^1^H NMR (400 MHz, CDCl_3_) δ 10.00 (d, J = 1.8 Hz, 1H), 9.83 (d, J = 2.6 Hz, 1H), 7.91 (d, J = 1.2 Hz, 1H), and 7.71 (dd, J = 11.1, 1.6 Hz, 1H). ^13^C NMR (101 MHz, CDCl_3_) δ 192.7 (s, 1C), 188.6 (s, 1C), 150.8 (d, ^1^J_C-F_ = 245.4 Hz), 143.9 (d, ^2^J_C-F_ = 14.2 Hz), 135.5 (s), 125.2(s), 119.2 (s), and 117.1 (d, ^2^J_C-F_ = 18.2 Hz). HRMS m/z (ESI) [M + Na]^+^ calculated for C_8_H_6_FNO_2_Na, 190.0280; found, 190.0275. IR neat: 3,447, 2,874, 2,822, 1,635, and 1,583 cm^−1^.

### 4-Amino-6-Methoxyisophthalaldehyde (3m)

Yield, 78% (27.9 mg); yellow solid; melting point, 132–135°C, ^1^H NMR (400 MHz, CDCl_3_) δ 10.18 (s, 1H), 9.80 (s, 1H), 8.11 (s, 1H), 6.06 (s, 1H), and 3.96 (s, 3H). ^13^C NMR (101 MHz, CDCl_3_) δ 192.6 (s, 1C), 186.9 (s, 1C), 166.3 (s, 1C), 156.2 (s, 1C), 139.7 (s, 1C), 116.7 (s, 1C), 113.5 (s, 1C), 95.8 (s, 1C), and 55.9 (s, 1C). HRMS m/z (ESI) [M + Na]^+^ calculated for C_9_H_9_NO_3_Na, 202.0480; found, 202.0475. IR neat: 3,426, 1,656, and 1,603 cm^−1^.

### 4-Amino-6-Isopropylisophthalaldehyde (3n)

Yield, 76% (29.1 mg); yellow solid; melting point, 100–102°C, ^1^H NMR (400 MHz, CDCl_3_) δ 10.05 (s, 1H), 9.92 (s, 1H), 8.03 (s, 1H), 6.63 (s, 1H), 4.01 (dt, J = 13.6, 6.8 Hz, 1H), and 1.29 (d, J = 6.8 Hz, 6H). ^13^C NMR (101 MHz, CDCl_3_) δ 193.1 (s, 1C), 189.4 (s, 1C), 158.9 (s, 1C), 153.5 (s, 1C), 143.5 (s, 1C), 123.6 (s, 1C), 116.6 (s, 1C), 112.9 (s, 1C), 28.3 (s, 1C), and 23.2 (s, 2C). HRMS m/z (ESI) [M + Na]^+^ calcd for C_11_H_13_NO_2_Na, 214.0844; found, 214.0839. IR neat: 3,426, 2,968, 2,874, 1,656, and 1,614 cm^−1^.

### 4-Amino-6-Ethylisophthalaldehyde (3o)

Yield, 78% (27.6 mg); yellow solid; melting point, 129–131°C, ^1^H NMR (400 MHz, CDCl_3_) δ 9.96 (s, 1H), 9.88 (s, 1H), 7.99 (s, 1H), 6.47 (s, 1H), 3.00 (q, J = 4.8 Hz, 2H), and 1.24 (t, J = 4.8 Hz, 3H). ^13^C NMR (101 MHz, CDCl_3_) δ 193.1 (s, 1C), 189.5 (s, 1C), 154.4 (s, 1C), 153.4 (s, 1C), 143.4 (s, 1C), 124.1 (s, 1C), 116.9 (s, 1C), 116.5 (s, 1C), 26.5 (s, 1C), and 15.2 (s, 1C). HRMS m/z (ESI) [M + Na]^+^ calculated for C_10_H_11_NO_2_Na, 200.0688; found, 200.0683. IR neat: 3,411, 2,957, 2,926, 1,646, and 1,610 cm^−1^.

### 4-Amino-6-Methylisophthalaldehyde (3p)

Yield, 80% (26.4 mg); yellow solid; melting point, 134–136°C, ^1^H NMR (400 MHz, CDCl_3_) δ 10.00 (s, 1H), 9.91 (s, 1H), 7.99 (s, 1H), 6.48 (s, 1H), and 2.64 (s, 3H). ^13^C NMR (101 MHz, CDCl_3_) δ 193.0 (s, 1C), 189.8 (s, 1C), 153.1 (s, 1C), 148.2 (s, 1C), 143.1 (s, 1C), 124.8 (s, 1C), 118.1 (s, 1C), 116.8 (s, 1C), and 20.5 (s, 1C). HRMS m/z (ESI) [M + Na]^+^ calculated for C_9_H_9_NO_2_Na, 186.0531; found, 186.0526. IR neat: 3,426, 2,957, 2,915, 1,645, and 1,603 cm^−1^.

### 4-Amino-6-Bromoisophthalaldehyde (3q)

Yield, 52%, (23.7 mg); yellow solid; melting point, 152–154°C, ^1^H NMR (400 MHz, CDCl_3_) δ 10.08 (s, 1H), 9.89 (s, 1H), 8.18 (s, 1H), and 6.92 (s, 1H). ^13^C NMR (400 MHz, CDCl_3_) δ 192.4 (s, 1C), 188.3 (s, 1C), 137.9 (s, 1C), 133.3 (s, 1C), 123.9 (s, 1C), 122.2 (s, 1C), 118.7 (s, 1C), and 116.8 (s, 1C). HRMS m/z (ESI) [M + Na]^+^ calculated for C_8_H_6_BrNO_2_Na, 249.9480; found, 249.9475. IR neat: 3,436, 2,957, 2,915, 1,666, and 1,614 cm^−1^.

### 4-Amino-6-Chloroisophthalaldehyde (3r)

Yield, 52% (19.1 mg); yellow solid; melting point 140–142°C, ^1^H NMR (400 MHz, CDCl_3_) δ 10.20 (s, 1H), 9.89 (s, 1H), 8.17 (s, 1H), and 6.69 (s, 1H). ^13^C NMR (101 MHz, CDCl_3_) δ 1 92.2 (s, 1C), 186.2 (s, 1C), 152.5 (s, 1C), 143.1 (s, 1C), 137.9 (s, 1C), 133.5 (s, 1C), 121.3 (s, 1C), and 115.1 (s, 1C). HRMS m/z (ESI) [M + Na]^+^ calculated for C_8_H_6_ClNO_2_Na, 205.9985; found, 205.9980. IR neat: 3,436, 2,968, 2,926, 1,656, and 1,603 cm^−1^.

### 4-Amino-6-Fluoroisophthalaldehyde (3s)

Yield, 50% (16.7 mg); yellow solid; melting point, 121–123°C, ^1^H NMR (400 MHz, CDCl_3_) δ 10.08 (s), 9.88 (s), 8.15 (d, J = 7.6 Hz), and 6.34 (d, J = 12.4 Hz). ^13^C NMR (101 MHz, CDCl_3_) δ 192.8 (s, 1C), 184.6 (s, 1C), 155.8 (s, 1C), 143.3 (s, 1C), 139.8 (d, ^2^J_C-F_ = 8.6 Hz, 1C), 123.0 (d, ^1^J_C-F_ = 211.99 Hz, 1C), 116.2 (s, 1C), and 101.3 (d, ^2^J_C-F_ = 24.1 Hz, 1H). HRMS m/z (ESI) [M + Na]^+^ calculated for C_8_H_6_FNO_2_Na, 190.0280; found, 190.0275. IR neat: 3,430, 2,979, 2,926, 1,660, and 1,609 cm^−1^.

### 4-Amino-5,6-Dimethylisophthalaldehyde (3t)

Yield, 70% (24.8 mg); yellow solid; melting point, 134–136°C, ^1^H NMR (400 MHz, CDCl_3_) δ 10.03 (s, 1H), 9.89 (s, 1H), 7.89 (s, 1H), 2.67 (s, 3H), and 2.12 (s, 3H). ^13^C NMR (101 MHz, CDCl_3_) δ 192.4 (s, 1C), 189.5 (s, 1C), 150.9 (s, 1C), 144.4 (s, 1C), 140.4 (s, 1C), 123.7 (s, 1C), 120.5 (s, 1C), 115.0 (s, 1C), 14.9 (s, 1C), and 10.7 (s, 1C). HRMS m/z (ESI) [M + Na]^+^ calculated for C_10_H_11_NO_2_Na, 200.0688; found, 200.0683. IR neat: 3,412, 2,959, 2,911, 1,625, and 1,603 cm^−1^.

### 7-Amino-2,3-Dihydro-1H-Indene-4,6-Dicarbaldehyde (3u)

Yield, 65%, (24.5 mg); yellow solid; melting point 134–136°C; ^1^H NMR (400 MHz, CDCl_3_) δ 9.92 (s, 1H), 9.89 (s, 1H), 7.87 (s, 1H), 3.33 (t, J = 7.6 Hz, 2H), 2.71 (t, J = 7.5 Hz, 2H), and 2.27–2.19 (m, 2H).^13^C NMR (101 MHz, CDCl_3_) δ 193.2 (s, 1C), 189.9 (s, 1C), 153.2 (s, 1C), 149.9 (s, 1C), 141.6 (s, 1C), 129.9 (s, 1C), 123.1 (s, 1C), 117.2 (s, 1C), 33.2 (s, 1C), 28.0 (s, 1C), and 24.1 (s, 1C). HRMS m/z (ESI) [M + Na]^+^ calculated for C_14_H_11_NO_2_Na, 248.0687; found, 248.0682. IR neat: 3,446, 2,853, 2,824, 1,603, and 1,572 cm^−1^.

### 4-Aminonaphthalene-1,3-Dicarbaldehyde (3v)

Yield, 51% (20.3 mg); yellow solid; melting point, 192–194°C, ^1^H NMR (400 MHz, CDCl_3_) δ 10.00 (s, 1H), 9.93 (s, 1H), 9.35 (d, J = 8.6 Hz, 1H), 7.94 (s, 1H), 7.89 (d, J = 8.5 Hz, 1H), 7.76–7.71 (m, 1H), and 7.57–7.52 (m, 1H). ^13^C NMR (101 MHz, CDCl_3_) δ 192.8 (s, 1C), 191.1 (s, 1C), 153.2 (s, 1C), 148.5 (s, 1C), 145.0(s, 1C), 132.4 (s, 1C), 126.8 (s, 1C), 126.5 (s, 1C), 122.1 (s, 1C), 121.6 (s, 1C), 121.4 (s, 1C), and 111.4 (s, 1C). HRMS m/z (ESI) [M + Na]^+^ calculated for C_12_H_9_NO_2_Na, 222.0531; found, 222.0526. IR neat: 3,400, 2,843, 2,739 1,635, and 1,600 cm^−1^.

### 4-(Methylamino)Isophthalaldehyde (3w)

Yield, 79%, (25.8 mg); white solid; melting point, 107–109°C, ^1^H NMR (400 MHz, CDCl_3_) δ 9.91 (s, 1H), 9.81 (s, 1H), 8.94 (s, 1H), 8.03 (d, J = 2.0 Hz, 1H), 7.96 (dd, J = 8.9, 1.6 Hz, 1H), 6.80 (d, J = 8.9 Hz, 1H), and 3.06 (s, 3H). ^13^C NMR (101 MHz, CDCl_3_) δ 193.6 (s, 1C), 189.3 (s, 1C), 155.3 (s, 1C), 141.1 (s, 1C), 135.7 (s, 1C), 124.8 (s, 1C), 117.8 (s, 1C), 111.1 (s, 1C), and 29.4 (s, 1C). HRMS m/z (ESI) [M + Na]^+^ calculated for C_9_H_9_NO_2_Na, 186.0531; found, 186.0526. IR neat: 3,312, 2,926, 2,853, 1,656, and 1,614 cm^−1^.

### 2-Amino-5-Benzoylbenzaldehyde (3x)

Yield, 78%, (35.1 mg); yellow solid; melting point, 122–125°C, ^1^H NMR (400 MHz, CDCl_3_) δ 9.87 (s, 1H), 8.05 (d, J = 1.9 Hz, 1H), 7.91 (dd, J = 8.7, 2.0 Hz, 1H), 7.78–7.70 (m, 2H), 7.58 (t, J = 7.4 Hz, 1H), 7.49 (t, J = 7.5 Hz, 2H), and 6.72 (d, J = 8.7 Hz, 1H). ^13^C NMR (101 MHz, CDCl_3_) δ 194.3 (s, 1C), 193.8 (s, 1C), 153.0 (s, 1C), 140.1 (s, 1C), 138.2 (s, 1C), 136.8 (s, 1C), 131.9 (s, 1C), 129.5 (s, 2C), 128.4 (s, 2C), 125.9 (s, 1C), 117.5 (s, 1C), and 115.9 (s, 1C). HRMS m/z (ESI) [M + Na]^+^ calculated for C_11_H_11_NO_2_Na 212.0687; found, 212.0682. IR neat: 3,436, 2,957, 2,926, 1,656, and 1,593 cm^−1^.

### Methyl[(Methylsulfinyl)Methyl]Sulfane (4a)

Yield, 78%, (38.7 mg) transparent liquid; ^1^H NMR (400 MHz, CDCl_3_) δ 3.84 (s, 2H), 3.05 (s, 3H), and 2.45 (s, 3H). ^13^C NMR (101 MHz, CDCl_3_) δ 56.2 (s), 37.8 (s), and 16.8 (s). HRMS m/z (ESI) [M + Na]^+^ calculated for C_3_H_8_OS_2_Na, 146.9909; found, 146.9913.

## Results and Discussion

We commenced the study by choosing acetanilide (**1a**) as the model substrate and optimized the reaction conditions through catalyst, oxidants/radical initiators, and temperature (Table 1). Based on the yields obtained, it was found that the reaction of acetanilide (**1a**, 0.2 mmol), Selectfluor (0.5 mmol), copper (II) trifluoromethanesulfonate (0.04 mmol), water (50 μL), and potassium carbonate (0.4 mmol) in dimethyl sulfoxide (1 mL) at 120°C for 12 h provided the best result, yielding the product 4-aminobenzene-1,3-dicarbaldehyde (**3a**) in 78% based on **1a** (entry 1). The use of other copper (II) salts such as Cu(NO_3_)_2_·6H_2_O, CuSO_4_·5H_2_O, and CuCl_2_·H_2_O as the catalysts would lead to the generation of **3a** in lower yields 13%–45% (entries 2–4). It seems that the process of reaction was related to Cu^2+^ ions rather than anions. The decrease of yields was probably due to the strength of the coordination bonds between Cu^2+^ and their anions. This result was consistent with the finding that the use of other metal salts such as Ni(OTf)_2_, Zn(OTf)_2_, and CoSO_4_·H_2_O as the catalysts did not provide the corresponding product at all (entries 5–7). Selectfluor worked as a highly efficient radical initiator in this reaction. When Selectfluor was replaced with K_2_S_2_O_8_ or TBHP under the same conditions, no product could be obtained (entries 8 and 9). A temperature range of 120°C–140°C was found necessary for the formation of **3a**. Lower temperature would decrease the yield dramatically down to trace (100°C), and higher temperature did not bring about additional yield (76%, 140°C).

**TABLE 1:** Optimization of the formation of 4-aminobenzene-1,3-dicarbaldehyde (**3a**)^a^.

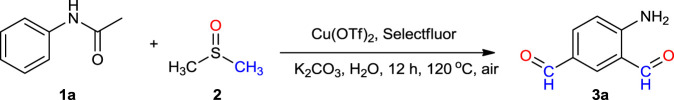				
En	Catalysts	Oxidants/Radical initiators	Temp. (°C)	Yields^b^
1	Cu(OTf)_2_	Selectfluor	120	78%
2	Cu(NO_3_)_2_·6H_2_O	Selectfluor	120	45%
3	CuSO_4_·5H_2_O	Selectfluor	120	43%
4	CuCl_2_·2H_2_O	Selectfluor	120	13%
5	Ni(OTf)_2_	Selectfluor	120	None
6	Zn(OTf)_2_	Selectfluor	120	None
7	CoSO_4_·H_2_O	Selectfluor	120	None
8	Cu(OTf)_2_	K_2_S_2_O_8_	120	None
9	Cu(OTf)_2_	TBHP	120	Trace
10	Cu(OTf)_2_	Selectfluor	100	Trace
11	Cu(OTf)_2_	Selectfluor	140	76%

a Reaction conditions: **1a** (0.2 mmol), **2** (1 mL), catalyst (0.04 mmol), Oxidants/Radical initiators (0.5 mmol), K_2_CO_3_ (0.4 mmol) and H_2_O (50 μL).

b Isolated yield.

**SCHEME 3:**
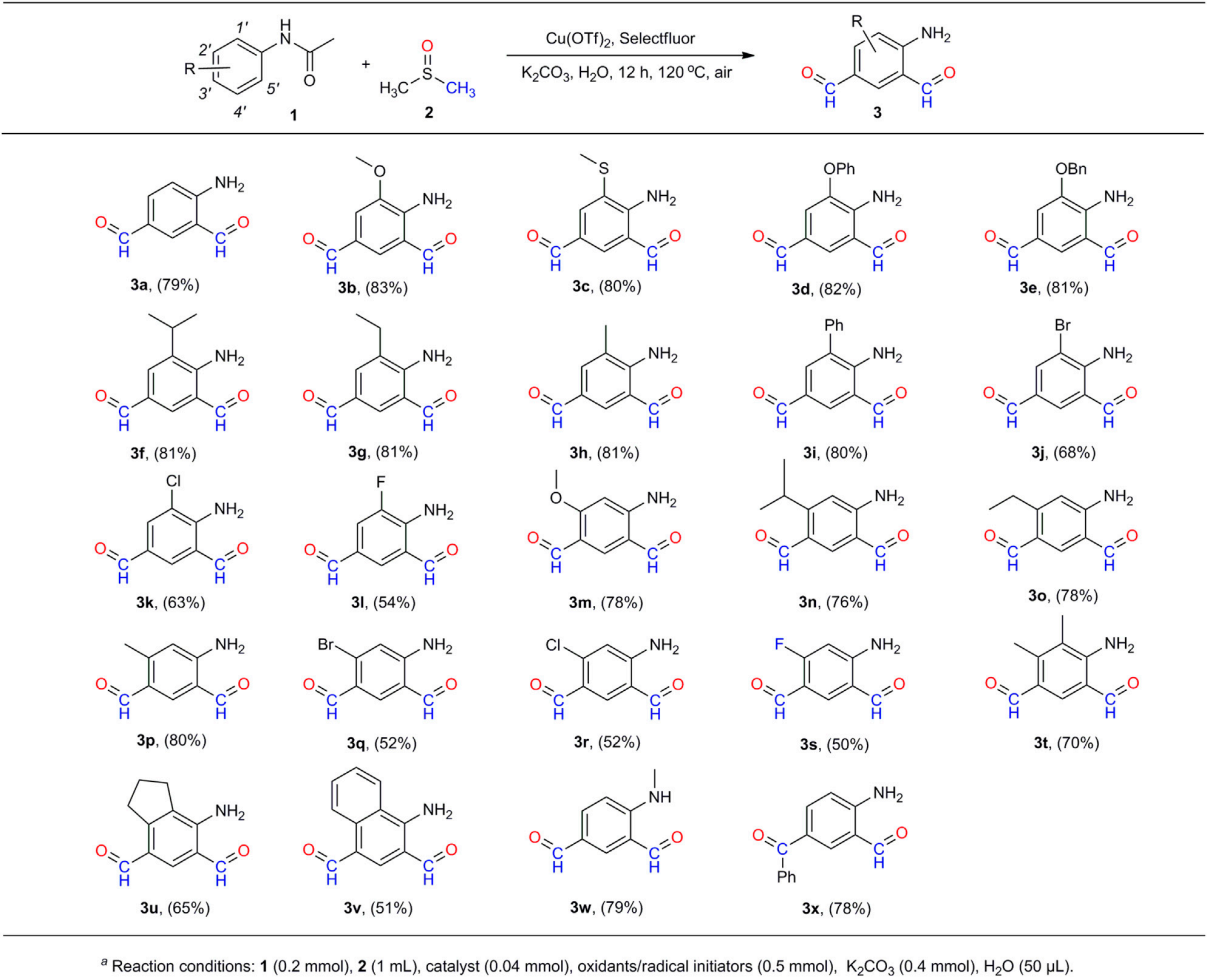
Scope of acetanilides with respect to 4-aminobenzene-1,3-dicarbaldehydes (**3**)^a^.

As the optimal reaction conditions were determined, we set out to explore the scope of **1** for the generation of compounds **3** (Scheme 3). The yields and production efficiency were discussed in terms of the electronic effect, steric effect, and synergic effect of the functional groups on the substrates. First, experimental results showed that the substitution of electron donating groups (EDGs) at the ortho-position of the phenyl rings [-OMe (**3b**), -SMe (**3c**), -OPh (**3d**), -OBn (**3e**), -iPr (**3f**), -Et (**3g**), -Me (**3h**), and -Ph (**3i**)] would be advantageous to the formation of compounds **3**, providing the corresponding products at similar yields (80%–83%). On the contrary, the substitution of electron withdrawing groups (EWGs) at the same position would have a negative effect on the proceeding of reaction. The stronger the electron-withdrawing ability, the poorer the production efficiency (**3j**–**3l**). No products could be obtained when acetanilides were substituted with the strong EWGs such as –CF_3_, –CN and –NO_2_. It is thought that the strong EWGs at the ortho-position of N-acetyl made the C–H bond inactive at the para-position of N-acetyl, which would stop the formylation at the para-position (*3*′-position) and thereby the ortho-position (*5*′-position) of the N-acetyl group. Similar results were obtained with the substitution of the functional groups at the meta-position (*2*′-position) of N-acetyl (**3m**–**3s**).

**SCHEME 4:**
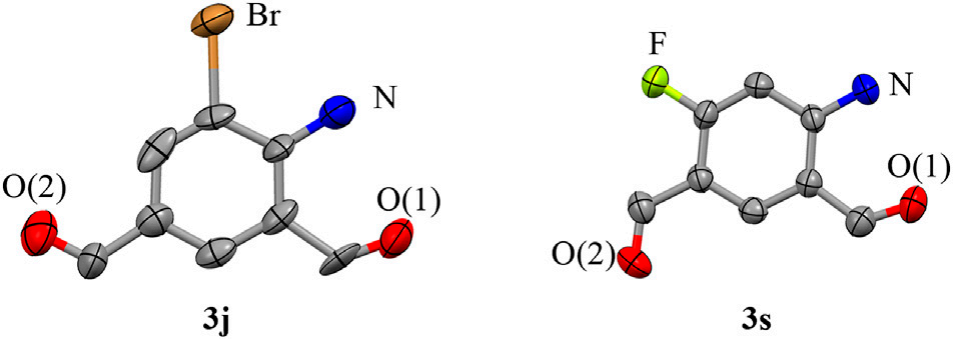
Crystal structures of compounds **3j** (CCDC 2153612) and **3s** (CCDC 2156314) with all non-hydrogen atoms shown as 50% probability ellipsoids.

Second, the steric hindrance of the functional groups on the substrates to the reactions was studied by the use of methoxyl- (**3b**, **3m**), isopropyl- (**3f**, **3n**), ethyl- (**3g**, **3o**), methyl- (**3h 3p**), bromo- (**3j**, **3q**), chloro- (**3k**, **3r**), and fluoro- (**3l**, **3s**) groups at the ortho- (*1*′-position) and meta-positions (*2*′-position) of the N-acetyl group. It is found that substitution of the functional groups at either of these two positions had a relatively weak impact on the production of the target compounds. Added to this, substitution of both these positions (both *1*′- and *2*′-positions) have certain unfavorable effects on the formation of **3**, with the decline of yields by about 15% (**3t**–**3v**). It is notable that the reaction of the disubstituted acetanilides at the *1*′, *4*′-positions (*5*′, *2*′-positions) or *2*′, *4*′-positions did not generate the desired compounds. The presence of functional groups at the *4*′-position would push back the substrates from the dinuclear copper (II) core and stop the reaction (Scheme 6). Moreover, our method is also compatible with the substrates on which the N-acetyl group is substituted with functional groups. The reaction of N-methyl–substituted acetanilide afforded the corresponding compound **3w** in 79% yield. In addition, the reaction of p-benzoyl-acetanilide provided the mono-aldehyde compound **3x** in a similar yield (78%). The structures of compound **3j** and **3s** were determined by X-ray crystallography (Scheme 4).

**SCHEME 5:**
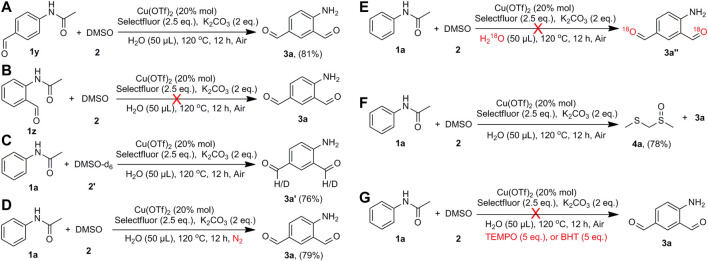
Mechanistic exploration of the formylation reaction.

With the progress of C_Ar_-H bond formylation, several experiments were carried out for the investigation of the reaction mechanism (Scheme 5). The reaction of N-(4-formylphenyl)acetamide (**1y**) with DMSO under the standard conditions afforded the compound **3a** in 81% yield (Scheme 5A). By comparison, the reaction of N-(2-formylphenyl)acetamide (**1z**) with DMSO at the same conditions did not generate **3a** (Scheme 5B), from which the starting material was recovered. It indicates that the formylation reaction took place in two consecutive steps. First, acetanilide was formylated at the para-position to form compound **1y**, and then **1y** was formylated at the ortho-position to produce the target compound **3a**. The reaction of acetanilide with DMSO-d_6_ afforded the compound **3a′** in 76% yield with 49% and 45% deuteration of the two formyl groups (Scheme 5C; Supplementary Figure S1). It suggests that the carbon atoms of the generated formyl groups likely came from DMSO. On the other hand, compound **3a** was obtained in similar yield when the experiment was conducted under nitrogen atmosphere (Scheme 5D). The use of H_2_^18^O as the additive to the reaction yielded the compound **3a** again (Scheme 5E; Supplementary Figure S2). Methyl [(methylsulfinyl)methyl]sulfane (**4a**) was isolated as a by-product in 78% yield in the reaction to form compound **3a** (Scheme 5F; Supplementary Figure S3). For these reasons, we proposed that the oxygen atom could come from DMSO rather than O_2_ and/or H_2_O. In addition, the formylation reaction was repeated under the standard conditions with the addition of either of the radical scavengers, 2,2,6,6-tetramethylpiperidinooxy (TEMPO) or butylated hydroxytoluene (BHT). No product was obtained with the addition of TEMPO or BHT, which indicated the generation of radicals in the process of the formylation of acetanilide (Scheme 5G). The attempt to detect the organic radical signals was unsuccessful since the presence of copper(II) compound in the solution would disturb the characteristic signals.

**SCHEME 6:**
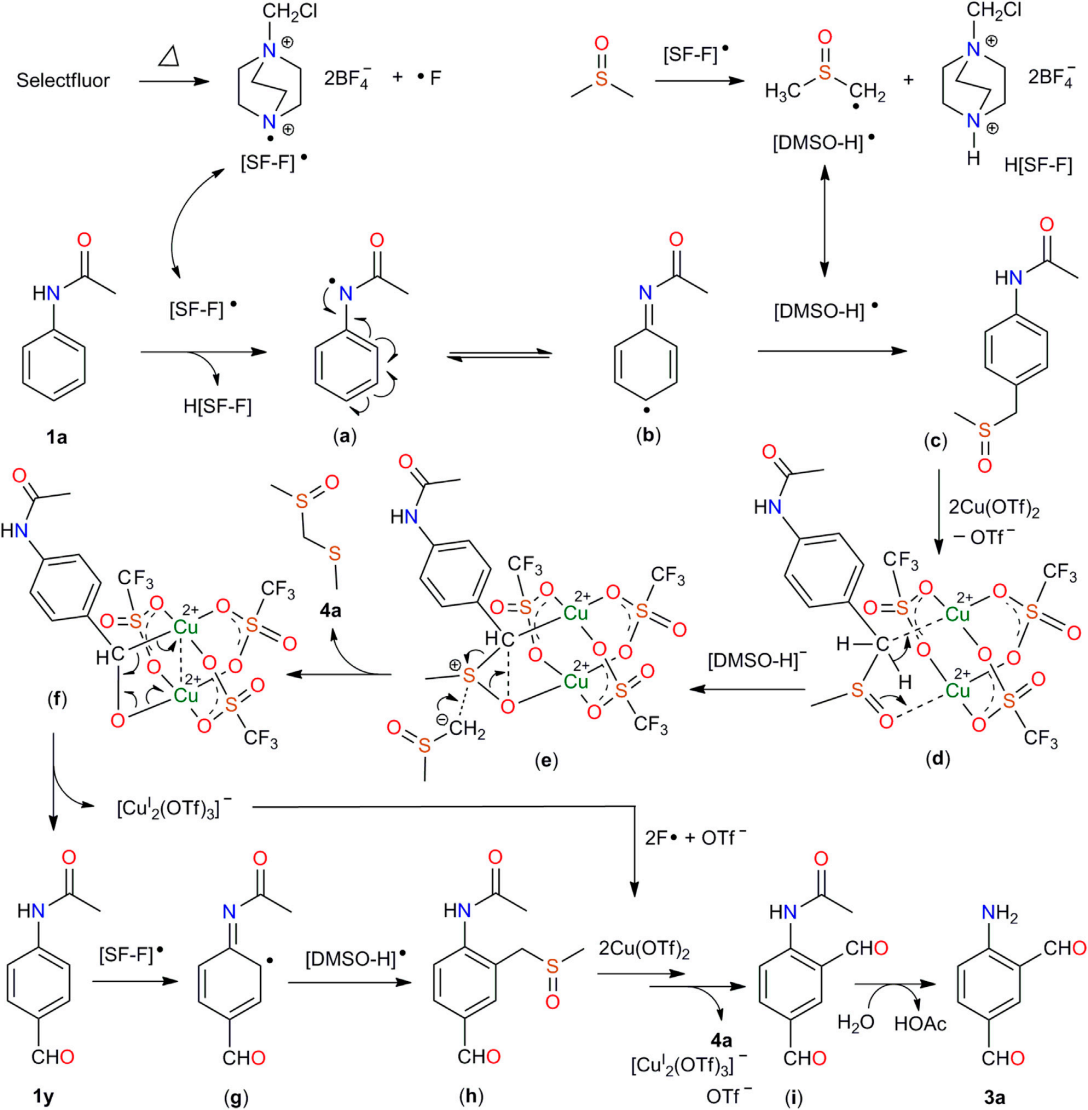
Proposed mechanism for the generation of compound **3a**.

The aforementioned results clearly indicated that first, a radical process was involved in this formylation; second, the carbon and oxygen atoms of the generated formyl groups come from DMSO rather than others; third, compound **1y** should be an intermediate in the reaction. On the basis of the experimental results and literature research (Gao et al., 2018; Sun et al., 2018; Niu et al., 2019; Yang et al., 2020), a possible mechanism was proposed in Scheme 6. The heating of Selectfluor generated the nitrogen radical [SF-F]•. The [SF-F]• attack DMSO and acetanilide (**1a**) to generate [DMSO-H]• and an N-acetyl radical (a), respectively. The latter undergoes intramolecular charge transfer to form 1,4-cyclohexadiene radical (b). Intermediate (b) reacts with [DMSO-H]• to yield (c). Addition of (c) to the dinuclear copper (II) core, followed by the attack of the deprotonated DMSO anion [(DMSO-H)^−^] results in (f), accompanied with the release of **4a** into the solution. Reduction of Cu(II) to Cu(I) leads to the decomposition of (f), by which N-(4-formylphenyl)acetamide (**1y**) is formed. Compound **1y** was attacked by [SF-F]• and then [DMSO-H]• to produce (h). Intermediate (h) is added to the dinuclear copper (II) core, followed by attack of [DMSO-H]^-^ and the reduction of Cu(II) to Cu(I) again to produce the final product **3a**.

## Conclusion

In summary, we have developed a simple and efficient method for the synthesis of 4-aminobenzene-1,3-dicarbaldehydes by formylation of acetanilides. The scope and versatility of the method have been successfully demonstrated with 24 examples. Experimental and mechanistic studies revealed a heat-induced radical reaction mechanism, in which copper(II) salt works as the catalyst; Selectfluor works as a radical initiator; DMSO works as a solvent and as the formyl source. In comparison with the description in the literature, our method is superior because of its simple work-up procedure, low-cost commercial available materials, air atmosphere, and one-step reaction. This work might provide a clue to synthesize valuable aminobenzaldehydes for further catalytic investigation.

## Data Availability

The original contributions presented in the study are included in the article/Supplementary Material, further inquiries can be directed to the corresponding author.
