# Supplementary material for: Copper(II)-Catalyzed Selective CAr-H Bond Formylation: Synthesis of Dialdehyde Aniline
Source: Front Chem. 2022 May 24;10:891858. doi: 10.3389/fchem.2022.891858 (PMC9171048; doi:10.3389/fchem.2022.891858)
Supplement: Supplementary file 1 [file DataSheet1.docx]

Supplementary Material

| **Table of contents:** |  |
| --- | --- |
| 1. ^1^H NMR, ^13^C NMR and HR-MS spectra of compounds shown in Scheme 5················· | 2 |
| 2. Crystallographic data of compounds································································ | 4 |
| 3. Characterization data of the starting materials····················································· | 5 |
| 4. ^1^H NMR and ^13^C NMR spectra of the starting materials········································· | 7 |
| 5. ^1^H NMR and ^13^C NMR spectra of products························································ | 18 |
| 6. IR spectrum of compound **3a**········································································ | 42 |

**1. ^1^H NMR, ^13^C NMR and HR-MS spectra of compounds shown in Scheme 5**


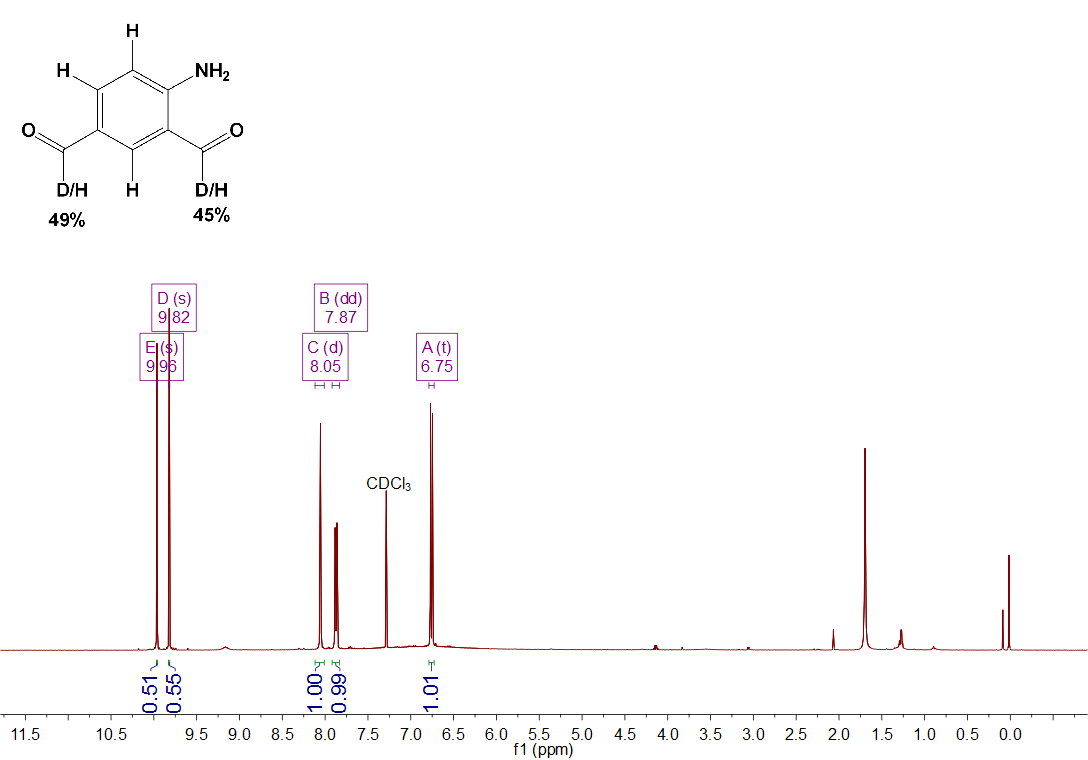


**Figure S1** ^1^H NMR (400 MHz, CDCl_3_) spectrum of compound **3a'** obtained from the DMSO-d_6_ isotope labeling experiment (Scheme 5C).

**
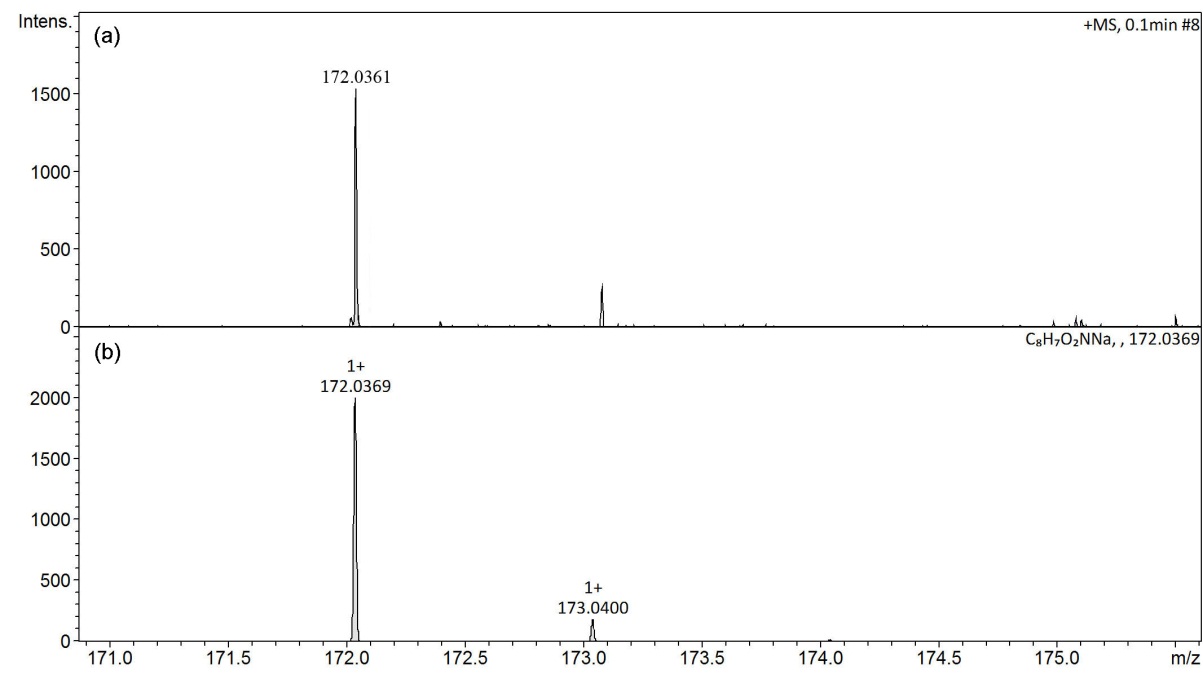
**

**Figure S2** (a) HR-MS spectrum of the product shown in Scheme 5E; (b) The theoretical MS signal of ([^16^O]-**3a** + Na^+^ ) given by the GC-MS database.

**
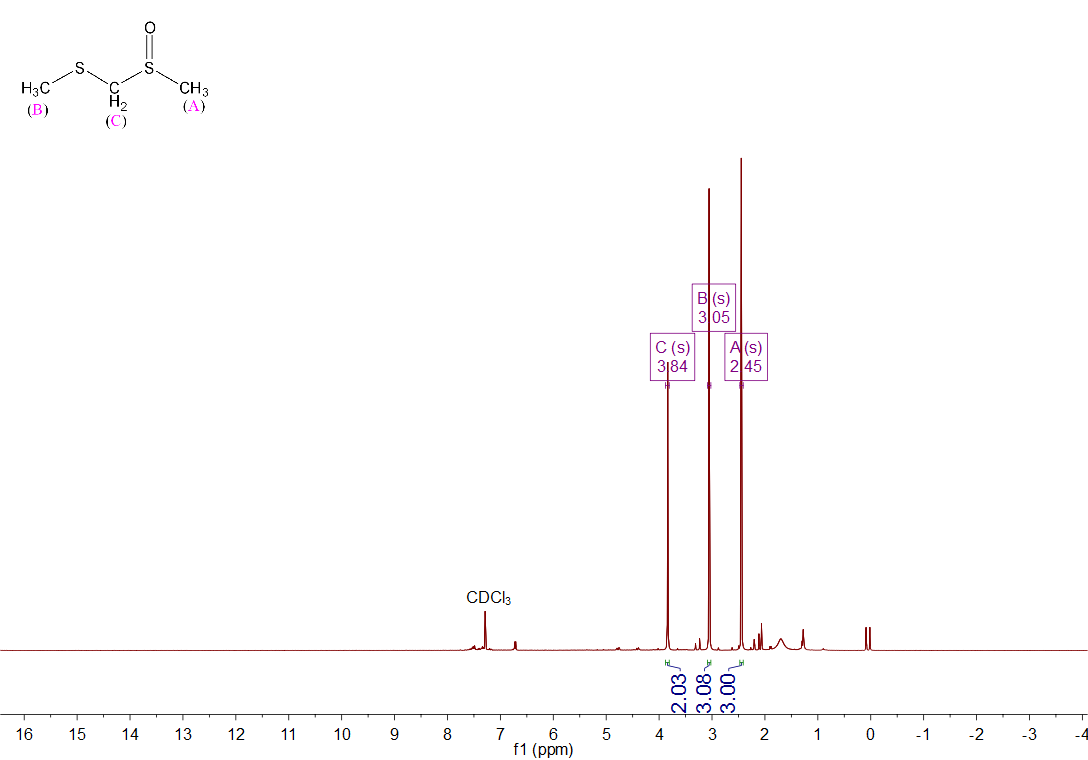

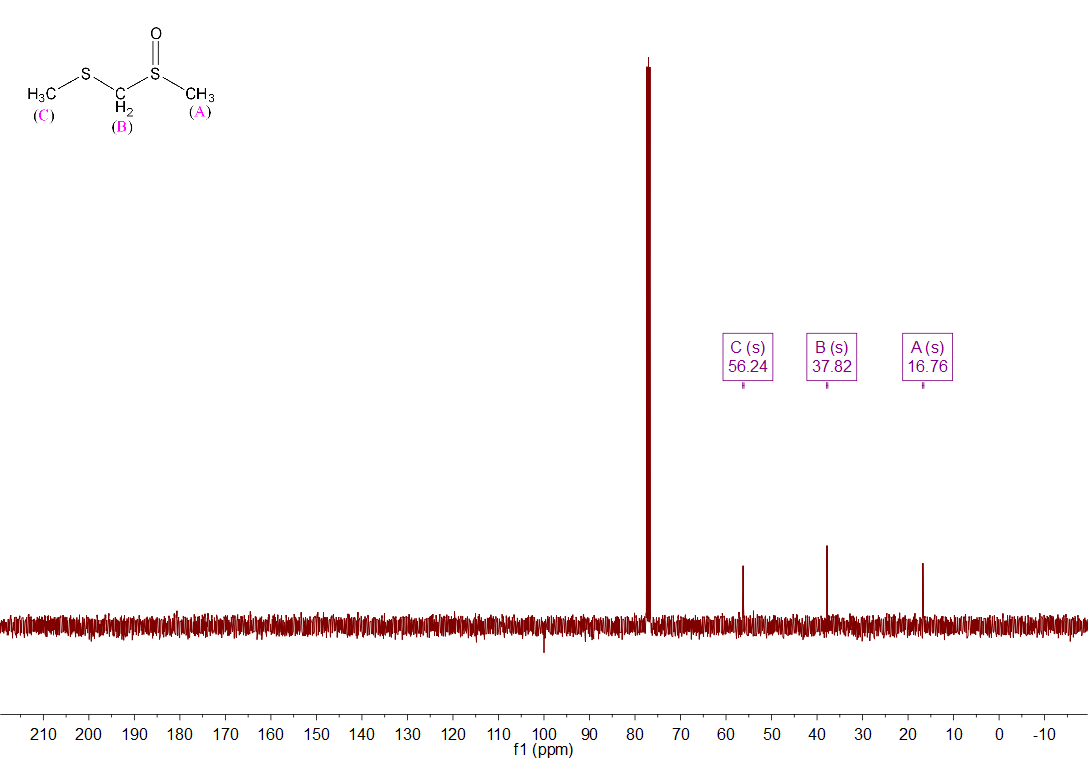
**

**Figure S3** ^1^H NMR (400 MHz, CDCl_3_) and ^13^C NMR (101 MHz, CDCl_3_) spectra of methyl((methylsulfinyl)methyl)sulfane (**4a**)

**2. Crystallographic data of compounds**

**Table S1** Crystallographic data*^a^* for compounds **3j** and **3s**

|  | *^a^***3j** | *^b^***3s** |
| --- | --- | --- |
| formula | C_8_H_6_BrNO_2_ | C_8_H_6_FNO_2_ |
| *M* | 228.05 | 167.14 |
| crystal system | monoclinic | monoclinic |
| space group | P 2_1_ | P 2_1_ |
| *a*, Å | 4.449(2) | 4.8326(5) |
| *b*, Å | 8.908(2) | 6.4023(4) |
| *c*, Å | 10.460(4) | 11.8362(9) |
| *α*, deg | 90.00 | 90 |
| *β*, deg | 95.48(4) | 99.004(8) |
| γ, deg | 90.00 | 90 |
| *V*, Å^3^ | 412.6(3) | 361.70(5) |
| Z | 2 | 2 |
| *μ*, mm^-1^ | 6.449 | 0.128 |
| independent data | 1478 | 1431 |
| refined parameters | 111 | 109 |
| *R_1_^c^, wR_2_^d^* (*I* > 2*σ*(*I*)) | 0.0922, 0.2728 | 0.0456, 0.0939 |
| *R_1_,wR_2_* (all data) | 0.1057, 0.2899 | 0.0645, 0.1054 |

*^a^*T = 273(2) K, Cu Kα radiation (*λ* = 1.54184 Å). *^b^*T = 288 (12) K, Mo Kα radiation (*λ* = 0.71073 Å). *^c^R_1_* = Σ||*F_o_*| – |*F_c_*||/Σ|*F_o_*|. *^d^wR_2_* = {Σ[w(*Fo*^2^ – *F_c_*^2^)^2^/(*F_o_*^2^)^2^]}^1/2^.

**3. Characterization data of the starting materials**

**N-(2-methoxyphenyl)acetamide (1b)**

^1^H NMR (400 MHz, CDCl_3_) δ 8.35 (dd, *J* = 7.9, 1.2 Hz, 1H), 7.77 (S, 1H), 7.03 (td, *J* = 7.9, 1.4 Hz, 1H), 6.95 (td, *J* = 7.8, 1.1 Hz, 1H), 6.87 (d, *J* = 8.1 Hz, 1H), 3.88 (s, 3H), 2.20 (s, 3H). ^13^C NMR (101 MHz, CDCl_3_) δ 168.2 (s, 1C), 147.6 (s, 1C), 127.7 (s, 1C), 123.6 (s, 1C), 121.1 (s, 1C), 119.8 (s, 1C), 109.9 (s, 1C), 55.7 (s, 1C), 25.0 (s, 1C).

**N-(2-(methylthio)phenyl)acetamide (1c)**

^1^H NMR (600 MHz, CDCl_3_) δ 8.25 (t, *J* = 11.1 Hz, 2H), 7.45 (dd, *J* = 7.7, 0.8 Hz, 1H), 7.27 (t, *J* = 7.8 Hz, 1H), 7.06 (t, *J* = 7.8 Hz, 1H), 2.36 (s, 3H), 2.23 (s, 3H). ^13^C NMR (151 MHz, CDCl_3_) δ 168.7 (s, 1C), 138.2 (s, 1C), 132.7 (s, 1C), 128.6 (s, 1C), 125.4 (s, 1C), 124.6 (s, 1C), 120.9 (s, 1C), 24.9 (s, 1C), 18.9 (s, 1C).

**N-(2-phenoxyphenyl)acetamide (1d)**

^1^H NMR (400 MHz, CDCl_3_) δ 8.23 (d, *J* = 8.2 Hz, 1H), 7.48 (t, *J* = 7.3 Hz, 2H), 7.42 (d, *J* = 7.2 Hz, 1H), 7.37 (M, 3H), 7.24 (d, *J* = 6.6 Hz, 1H), 7.22 – 7.13 (m, 2H), 2.01 (s, 3H). ^13^C NMR (101 MHz, CDCl_3_) δ 168.5 (s, 1C), 138.2 (s, 1C), 134.6 (s, 1C), 132.4 (s, 1C), 130.1 (s, 1C), 129.3 (s, 2C), 129.1 (s, 2C), 128.4 (s, 1C), 128.0 (s,1C), 124.5 (s, 1C), 121.9 (s, 1C), 24.6 (s, 1C).

**N-(2-ethylphenyl)acetamide (1g)**

^1^H NMR (400 MHz, CDCl_3_) δ 7.71 (d, *J* = 7.9 Hz, 1H), 7.28 (s, 1H), 7.22 (t, *J* = 6.9 Hz, 2H), 7.16 (d, *J* = 7.5 Hz, 1H), 2.62 (q, *J* = 7.6 Hz, 2H), 2.21 (s, 3H), 1.25 (t, *J* = 7.6 Hz, 3H). ^13^C NMR (101 MHz, CDCl_3_) δ 168.7 (s, 1C), 135.5 (s, 1C), 134.9 (s, 1C), 128.6 (s, 1C), 126.7 (s, 1C), 125.8 (s, 1C), 124.3 (s, 1C), 24.2 (s, 1C), 14.0 (s, 1C).

**Acetamidobiphenyl (1i)**

^1^H NMR (400 MHz, CDCl_3_) δ 8.43 (d, *J* = 8.1 Hz, 1H), 7.76 (s, 1H), 7.36 (t, *J* = 7.8 Hz, 2H), 7.15 (t, *J* = 7.5 Hz, 1H), 7.10 (d, *J* = 7.9 Hz, 1H), 7.00 (dd, *J* = 13.1, 7.8 Hz, 3H), 6.83 (d, *J* = 8.1 Hz, 1H), 2.17 (s, 3H). ^13^C NMR (101 MHz, CDCl_3_) δ 168.4 (s, 1C), 156.4 (s, 1C), 145.5 (s, 1C), 130.0 (s, 2C), 129.8 (s, 1C), 124.0 (s, 1C), 123.99 (s, 1C), 123.96 (s, 1H), 120.9 (s, 1C), 118.7 (s, 2C), 117.7 (s, 1C), 25.0 (s, 1C).

**N-(3-isopropylphenyl)acetamide (1n)**

^1^H NMR (400 MHz, CDCl_3_) δ 8.17 (s, 1H), 7.36 (d, *J* = 8.9 Hz, 2H), 7.20 (t, *J* = 7.7 Hz, 1H), 6.96 (d, *J* = 7.6 Hz, 1H), 2.84 (dt, *J* = 13.5, 6.7 Hz, 1H), 2.14 (s, 3H), 1.20 (d, *J* = 6.9 Hz, 6H). ^13^C NMR (101 MHz, CDCl_3_) δ 169.2 (s, 1C), 149.8 (s, 1C), 138.1 (s, 1C), 128.8 (s, 1C), 122.4 (s, 1C), 118.4 (s, 1C), 117.8 (s, 1C), 34.1 (s, 1C), 24.4 (s, 1C), 23.9 (s, 2C)

**N-(3-ethylphenyl)acetamide (1o)**

^1^H NMR (400 MHz, CDCl_3_) δ 7.46 (s, 1H), 7.36 (s, 1H), 7.31 (d, *J* = 8.2 Hz, 1H), 7.22 (t, *J* = 7.8 Hz, 1H), 6.95 (d, *J* = 7.5 Hz, 1H), 2.62 (q, *J* = 7.6 Hz, 2H), 2.16 (s, 3H), 1.22 (t, *J* = 7.6 Hz, 3H). ^13^C NMR (101 MHz, CDCl_3_) δ 168.5 (s, 1C), 145.3 (s, 1C), 137.9 (s, 1C), 128.9 (s, 1C), 123.9 (s, 1C), 119.4 (s, 1C), 117.3 (s, 1C), 28.9 (s, 1C), 24.6 (s, 1C), 15.5 (s, 1C).

**3-Chloroacetanlide (1r)**

^1^H NMR (400 MHz, CDCl_3_) δ 7.81 (s, 1H), 7.63 (s, 1H), 7.34 (d, *J* = 8.1 Hz, 1H), 7.21 (t, *J* = 8.1 Hz, 1H), 7.06 (d, *J* = 7.9 Hz, 1H), 2.17 (s, 3H). ^13^C NMR (101 MHz, CDCl_3_) δ 168.9 (s, 1C), 139.1 (s, 1C), 134.6 (s, 1C), 130.0 (s, 1C), 124.4 (s, 1C), 120.1 (s, 1C), 117.9 (s, 1C), 24.6 (s, 1C).

**N-(2,3-dimethylphenyl)acetamide (1t)**

^1^H NMR (400 MHz, CDCl_3_) δ 7.36 (d, *J* = 7.3 Hz, 1H), 7.16 (s, 1H), 7.08 (t, *J* = 7.7 Hz, 1H), 7.01 (d, *J* = 7.4 Hz, 1H), 2.28 (s, 3H), 2.18 (s, 3H), 2.12 (s, 3H). ^13^C NMR (101 MHz, CDCl_3_) δ 168.8 (s, 1C), 137.5 (s, 1C), 135.2 (s, 1C), 130.1 (s, 1C), 127.7 (s, 1C), 125.9 (s, 1C), 122.7 (s, 1C), 24.0 (s, 1C), 20.6 (s, 1C), 13.9 (s, 1C).

**N-indan-4-ylacetamide (1u)**

^1^H NMR (400 MHz, CDCl_3_) δ 7.72 (s, 1H), 7.55 (d, *J* = 5.2 Hz, 1H), 7.09 (t, *J* = 5.1 Hz, 1H), 7.01 (d, *J* = 4.9 Hz, 1H), 2.90 (t, *J* = 5.0 Hz, 2H), 2.78 (t, *J* = 4.9 Hz, 2H), 2.15 (s, 3H), 2.07 – 2.02 (m, 2H). ^13^C NMR (101 MHz, CDCl_3_) δ 169.2 (s, 1C), 145.5 (s, 1C), 135.3 (s, 1C), 133.6 (s, 1C), 127.1 (s, 1C), 121.3 (s, 1C), 119.9 (s, 1C), 33.2 (s, 1C), 30.3 (s, 1C), 24.9 (s, 1C), 24.1 (s, 1C).

**N-(4-benzoylphenyl)acetamide (1x)**

^1^H NMR (600 MHz, CDCl_3_) δ 8.34 (s, 1H), 7.77 (d, *J* = 8.7 Hz, 2H), 7.75 – 7.72 (m, 2H), 7.65 (d, *J* = 8.6 Hz, 2H), 7.56 (t, 1H), 7.45 (dd, *J* = 10.8, 4.7 Hz, 2H), 2.19 (s, 3H). ^13^C NMR (151 MHz, CDCl_3_) δ 196.1 (s, 1C), 169.2 (s, 1C), 142.3 (s, 1C), 137.8 (s, 1C), 132.8 (s, 1C), 132.5 (s, 1C), 131.7 (s, 1C), 130.0 (s, 1C), 128.4 (s, 1C), 118.9 (s, 1C), 24.8 (s, 1C).

**4. ^1^H NMR and ^13^C NMR spectra of the starting materials**

**^1^H NMR (400 MHz, CDCl_3_) and ^13^C NMR (101 MHz, CDCl_3_) of N-(2-methoxyphenyl)-acetamide (1b)**

**^
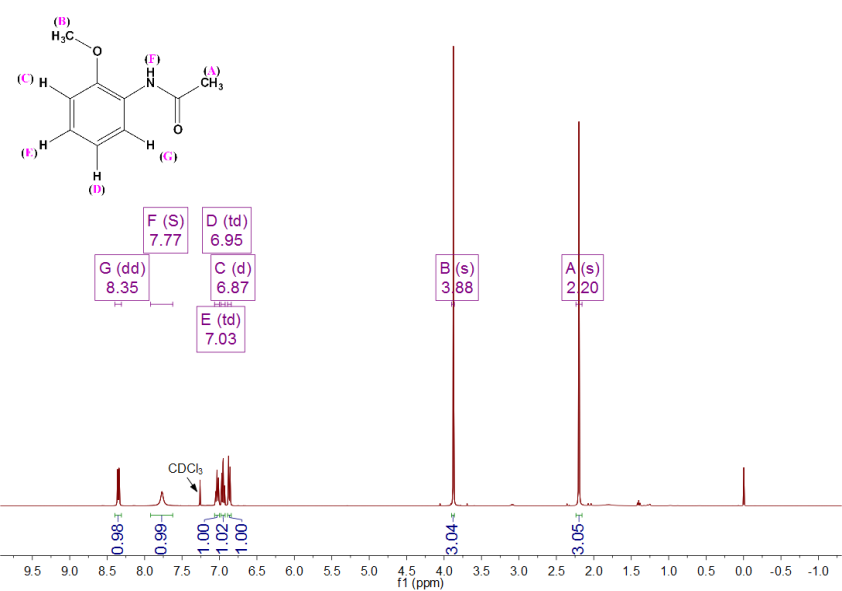

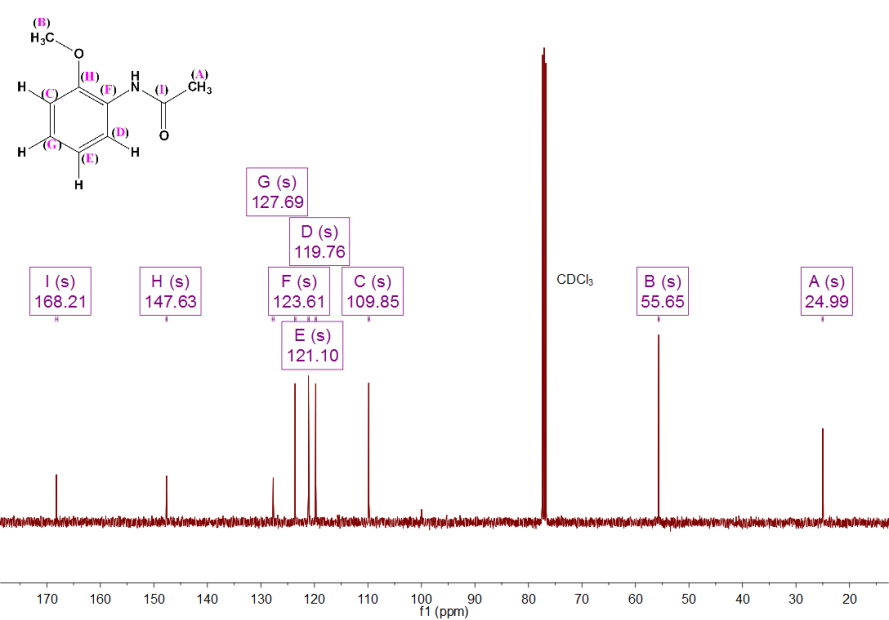
^**

**^1^H NMR (400 MHz, CDCl_3_) and ^13^C NMR (101 MHz, CDCl_3_) of N-(2-(methylthio)phenyl)-acetamide (1c)**

**
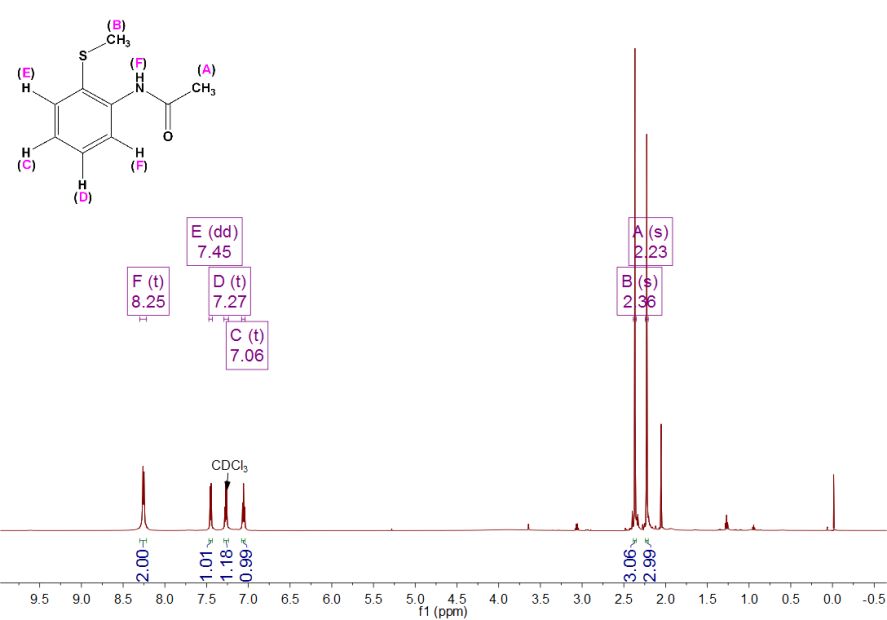
**
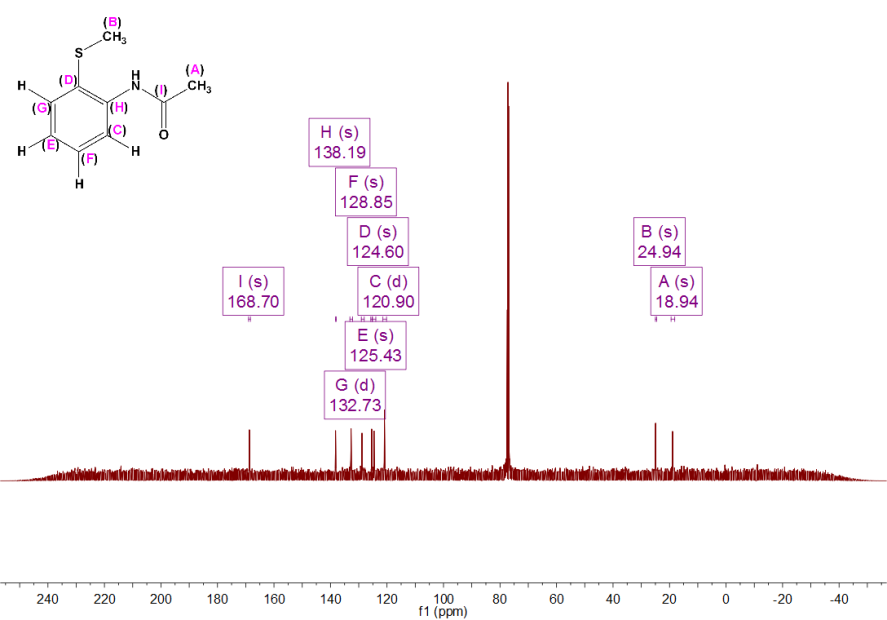


^1^H NMR (400 MHz, CDCl_3_) and ^13^C NMR (101 MHz, CDCl_3_) of N-(2-phenoxyphenyl)-acetamide (1d)^
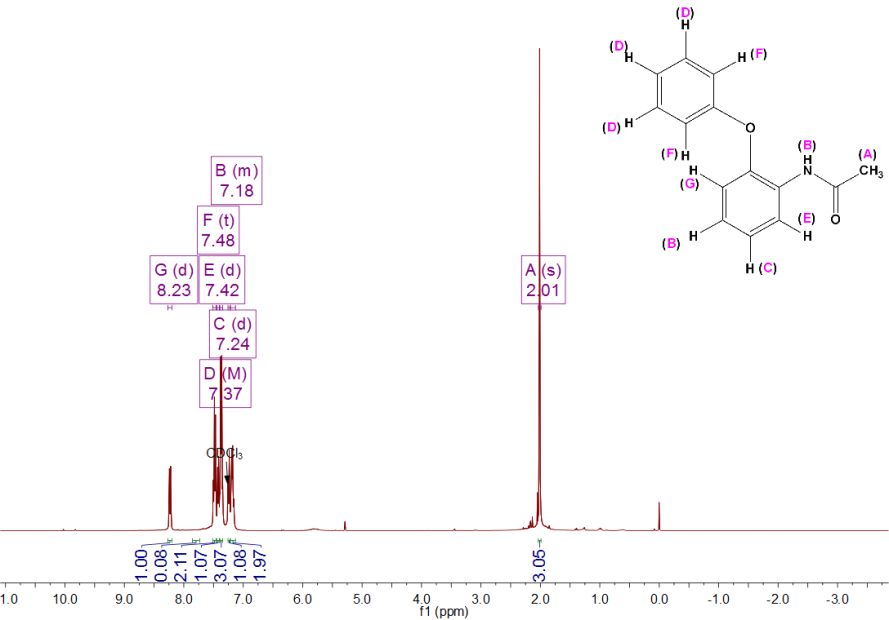

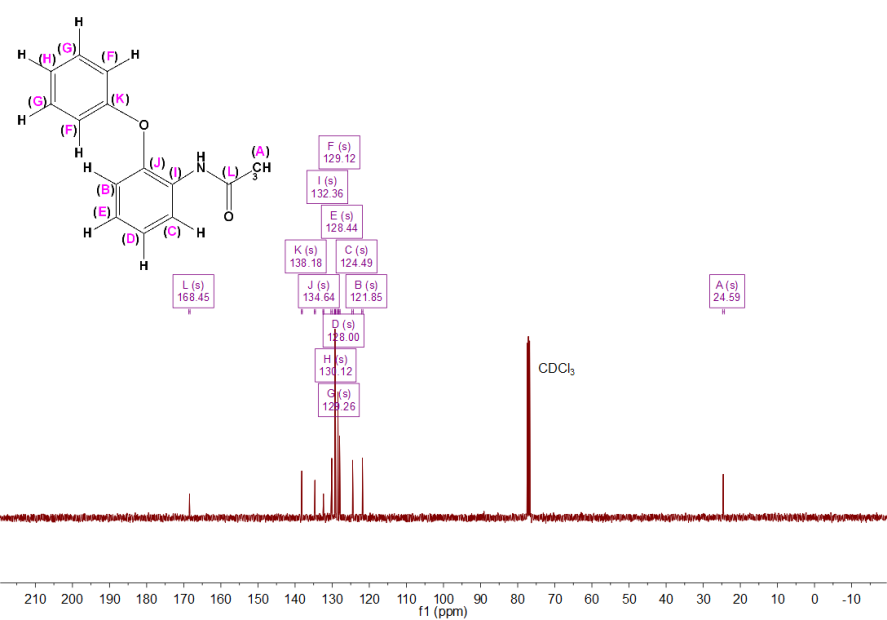
^

**^1^H NMR (400 MHz, CDCl_3_) and ^13^C NMR (101 MHz, CDCl_3_) of N-(2-ethylphenyl)acetamide (1g)**

**^
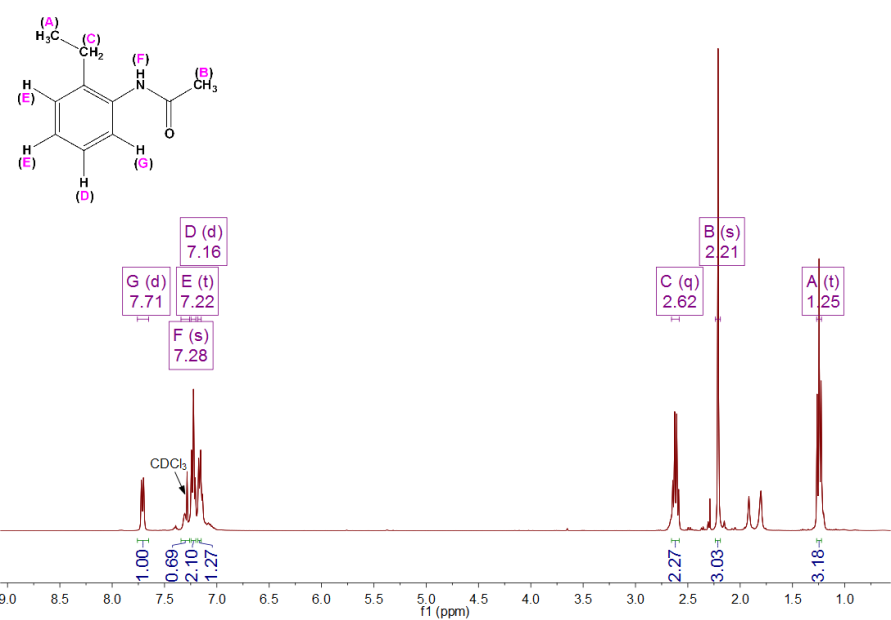

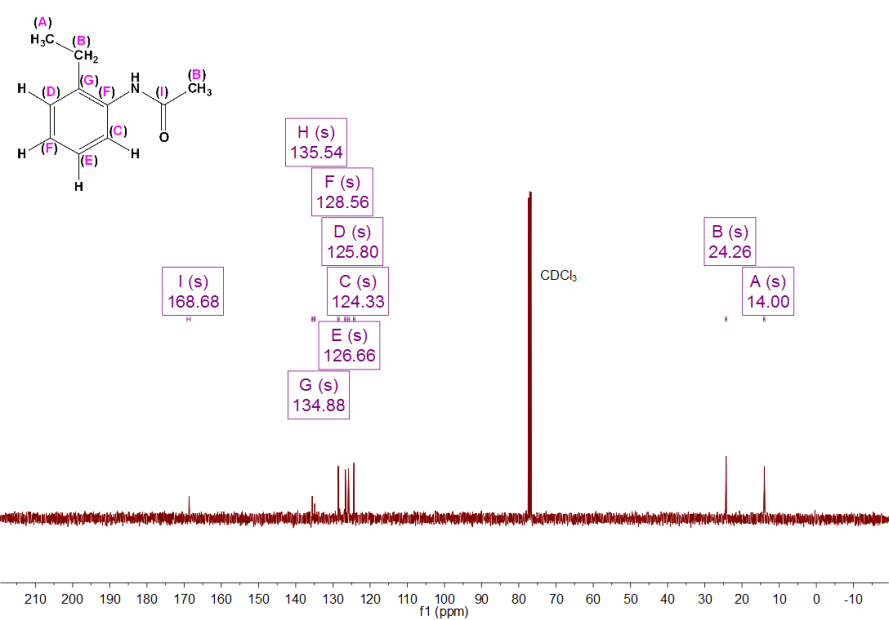
^**

**^1^H NMR (400 MHz, CDCl_3_) and ^13^C NMR (101 MHz, CDCl_3_) of acetamidobiphenyl (1i)**

**^
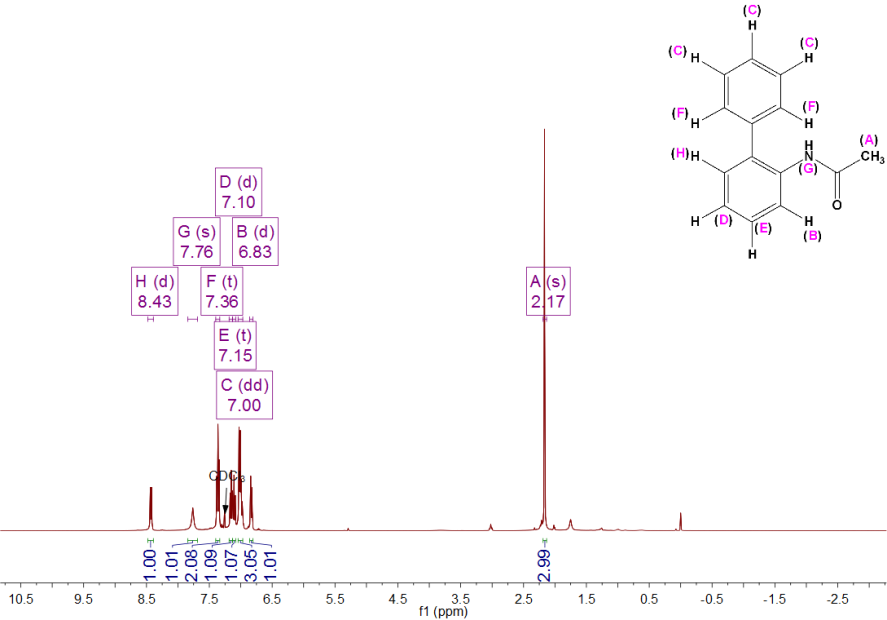

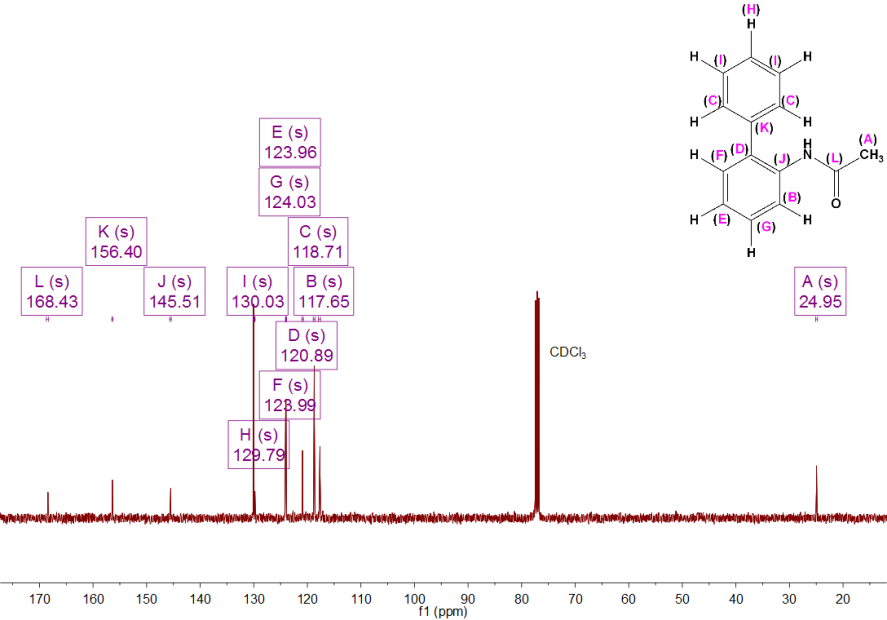
^**

**^1^H NMR (400 MHz, CDCl_3_) and ^13^C NMR (101 MHz, CDCl_3_) of N-(3-isopropylphenyl)-acetamide (1n)**

**^
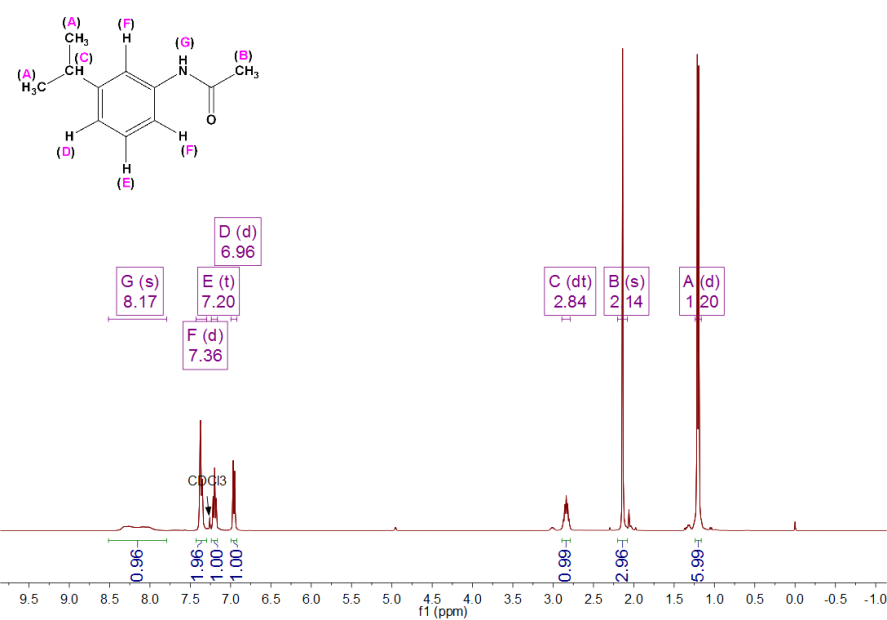

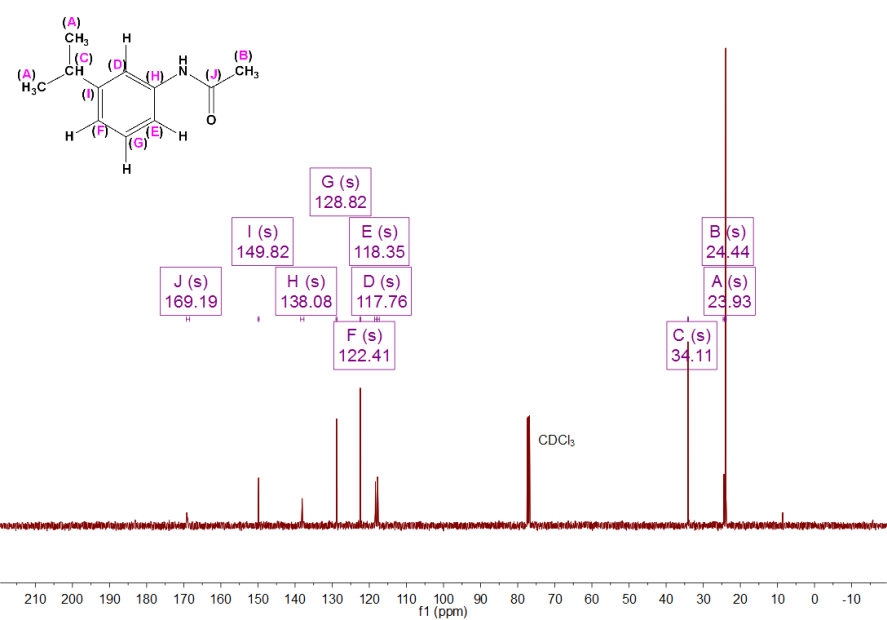
^**

**^1^H NMR (400 MHz, CDCl_3_) and ^13^C NMR (101 MHz, CDCl_3_) of N-(3-ethylphenyl)acetamide (1o)**

**^
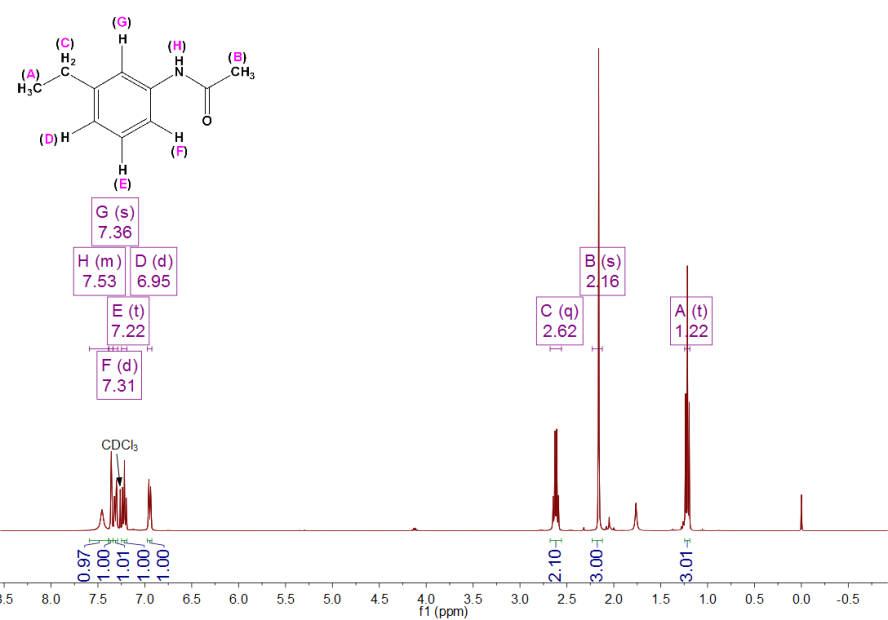

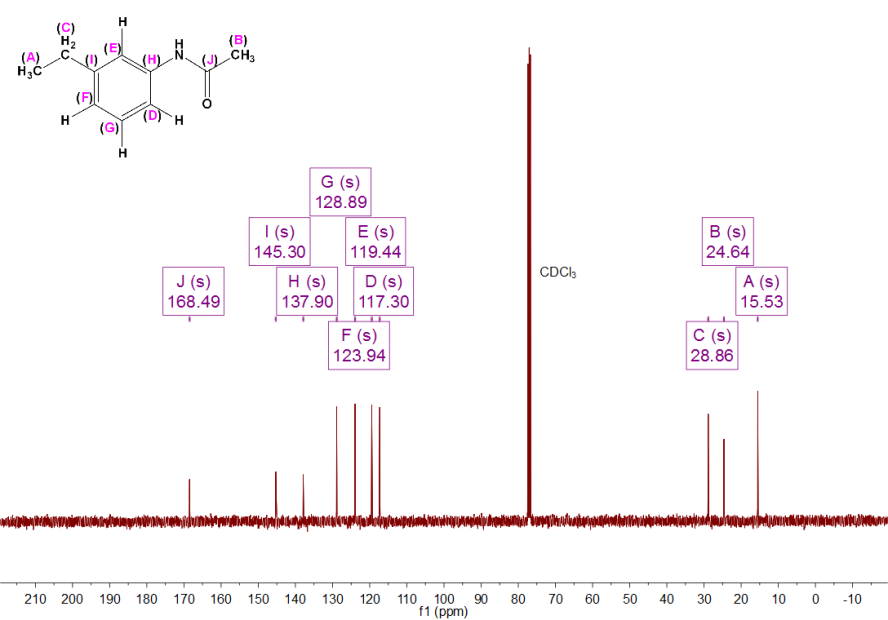
^**

**^1^H NMR (400 MHz, CDCl_3_) and ^13^C NMR (101 MHz, CDCl_3_) of 3-Chloroacetanlide (1r)**

**^
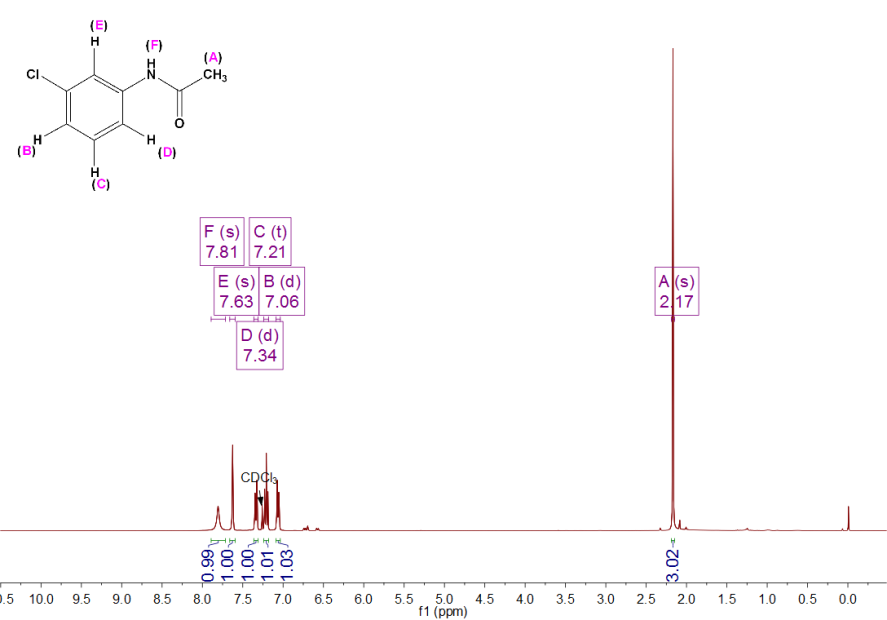

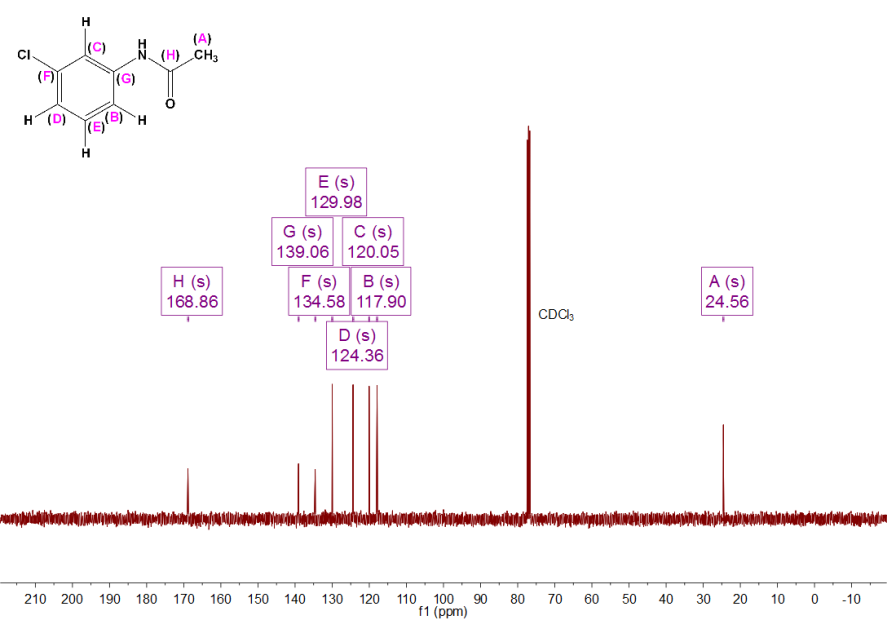
^**

**^1^H NMR (400 MHz, CDCl_3_) and ^13^C NMR (101 MHz, CDCl_3_) of N-(2,3-dimethylphenyl)-acetamide (1t)^
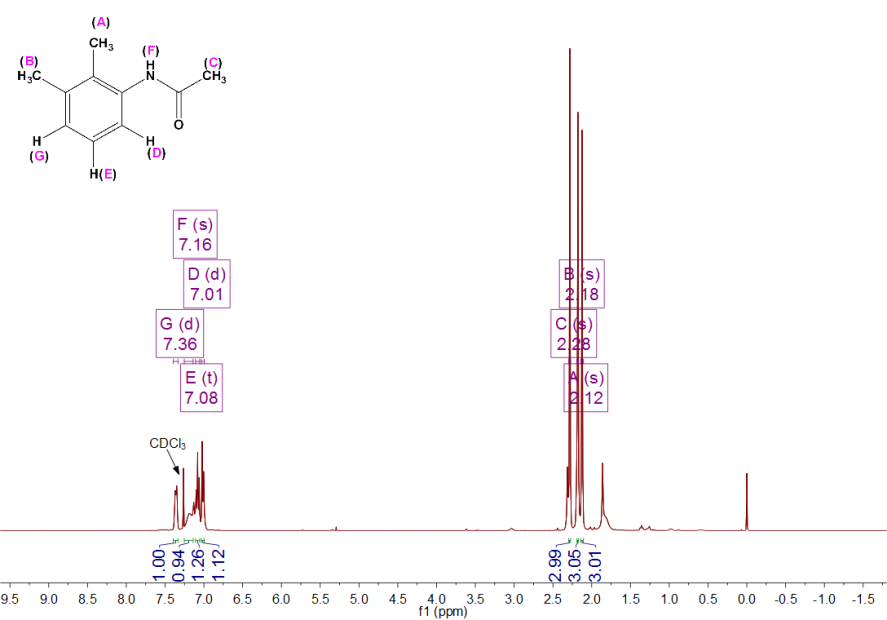

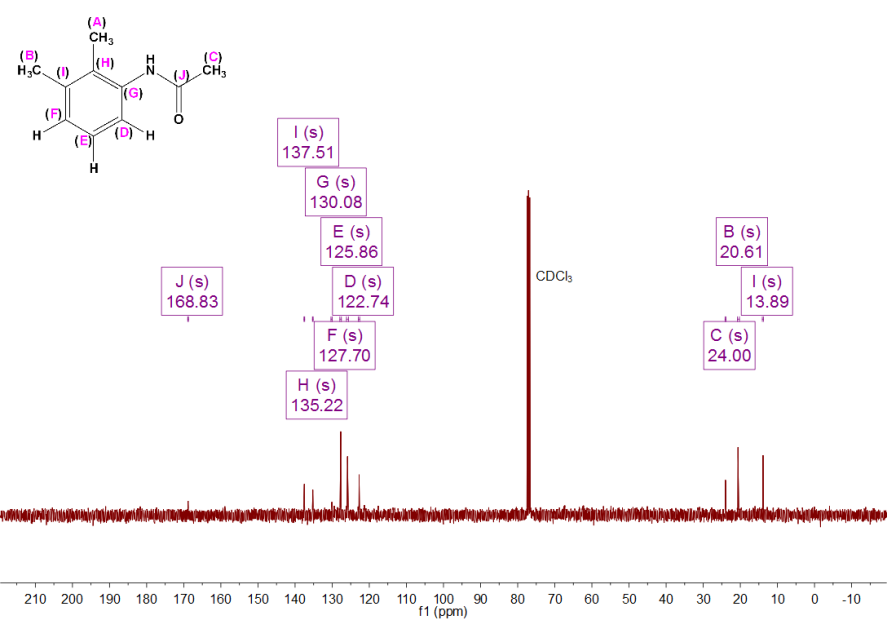
^**

**^1^H NMR (400 MHz, CDCl_3_) and ^13^C NMR (101 MHz, CDCl_3_) of N-(2,3-dihydro-1H-inden-4-yl)acetamide** **(1u)**^
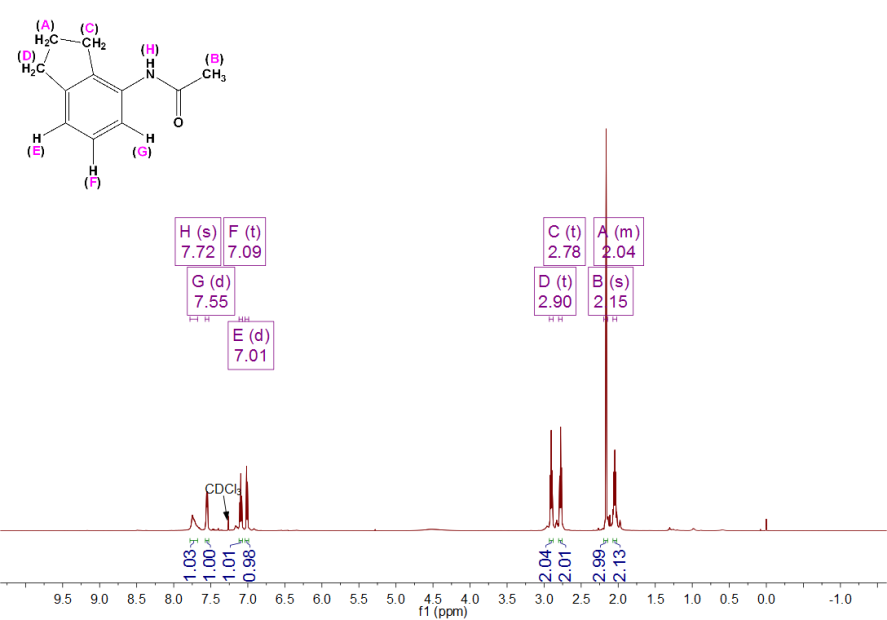

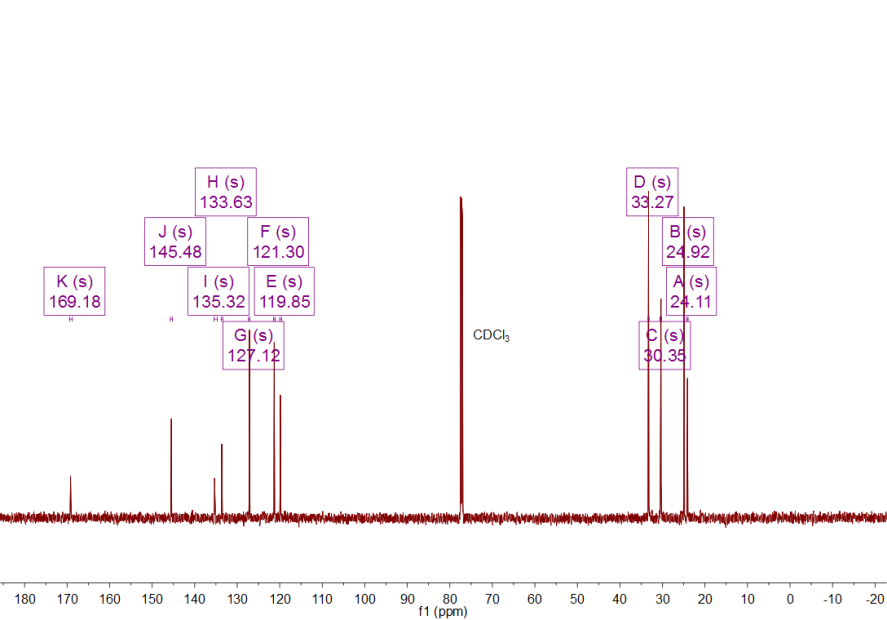
^

**^1^H NMR (400 MHz, CDCl_3_) and ^13^C NMR (101 MHz, CDCl_3_) of N-(4-benzoylphenyl)acetamide (1x)**

**^
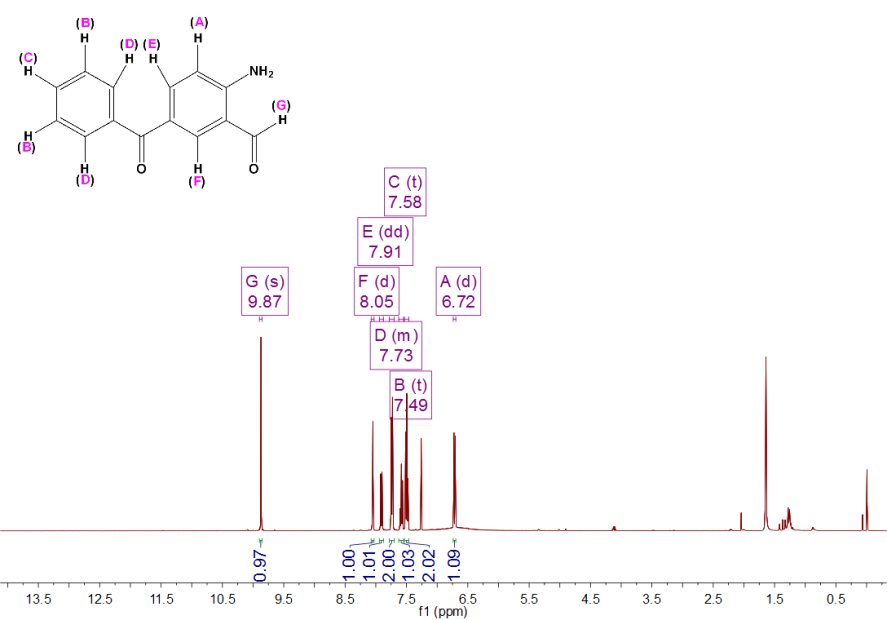

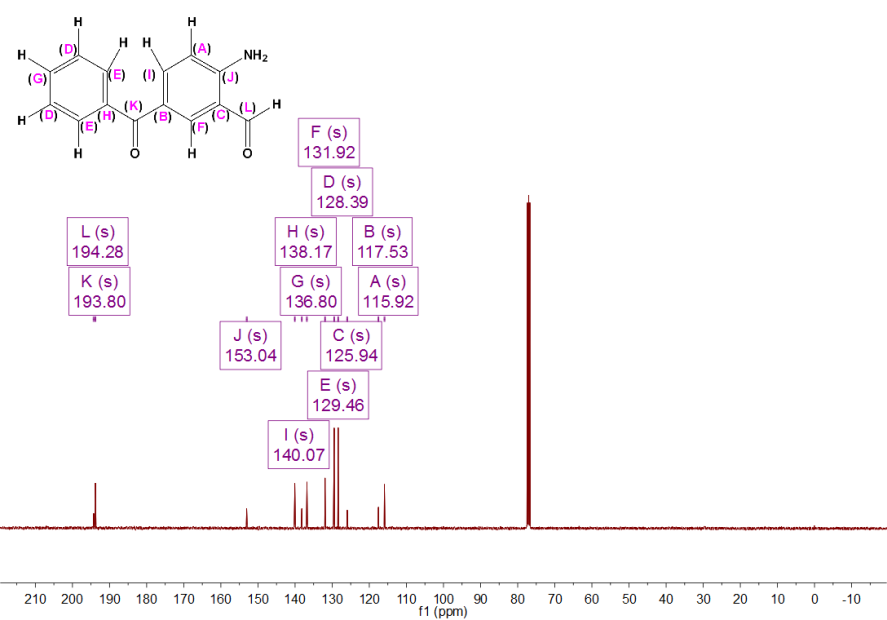
^**

**5. ^1^H NMR and ^13^C NMR spectra of products**

**^1^H NMR (400 MHz, CDCl_3_) and ^13^C NMR (101 MHz, CDCl_3_) of 4-aminoisophthalaldehyde (3a)**
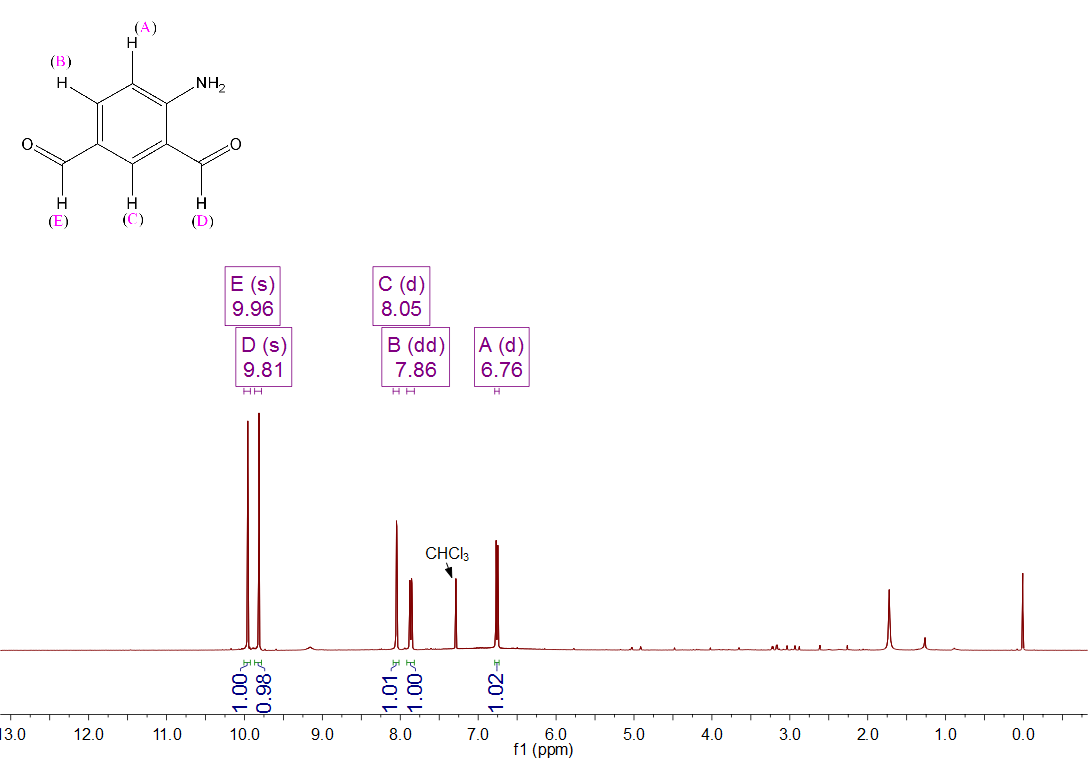

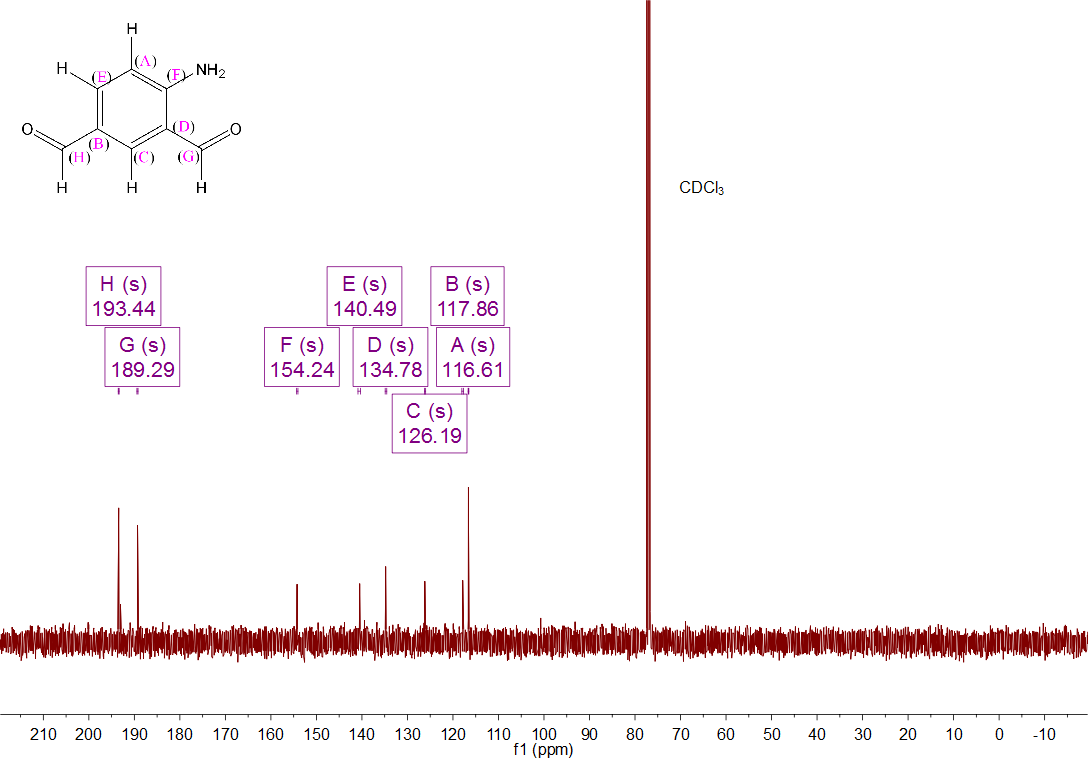


**^1^H NMR (400 MHz, CDCl_3_) and ^13^C NMR (101 MHz, CDCl_3_) of 4-amino-5-methoxyisophthal-aldehyde (3b)**


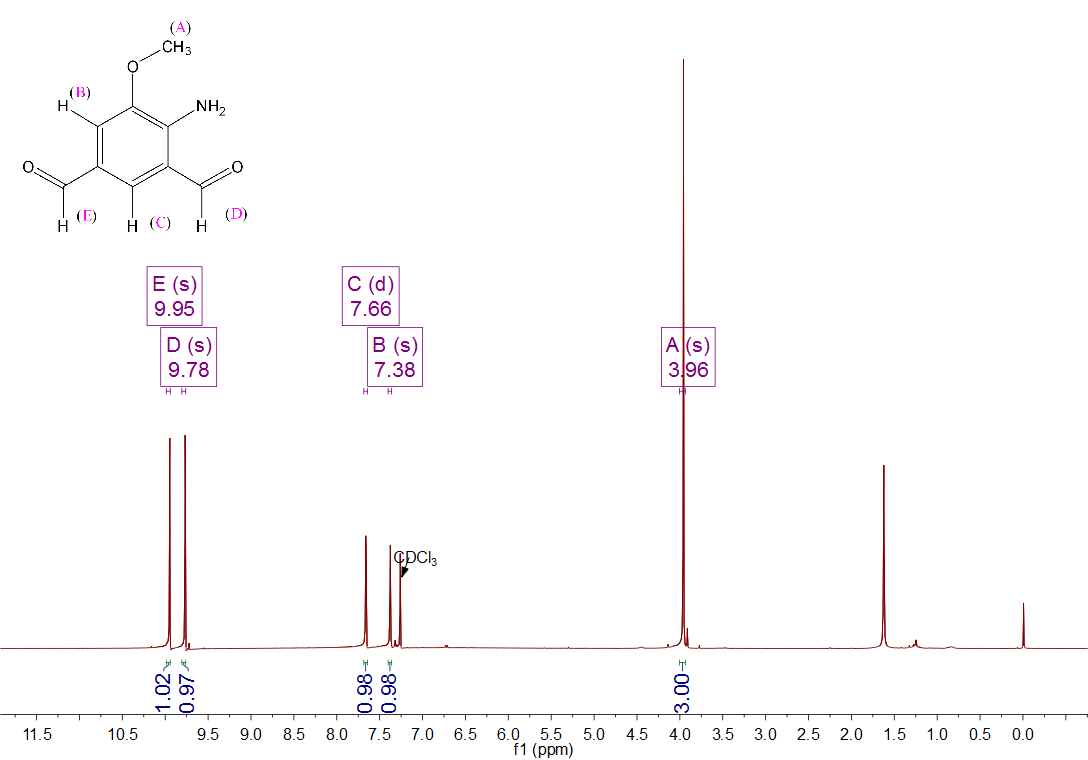


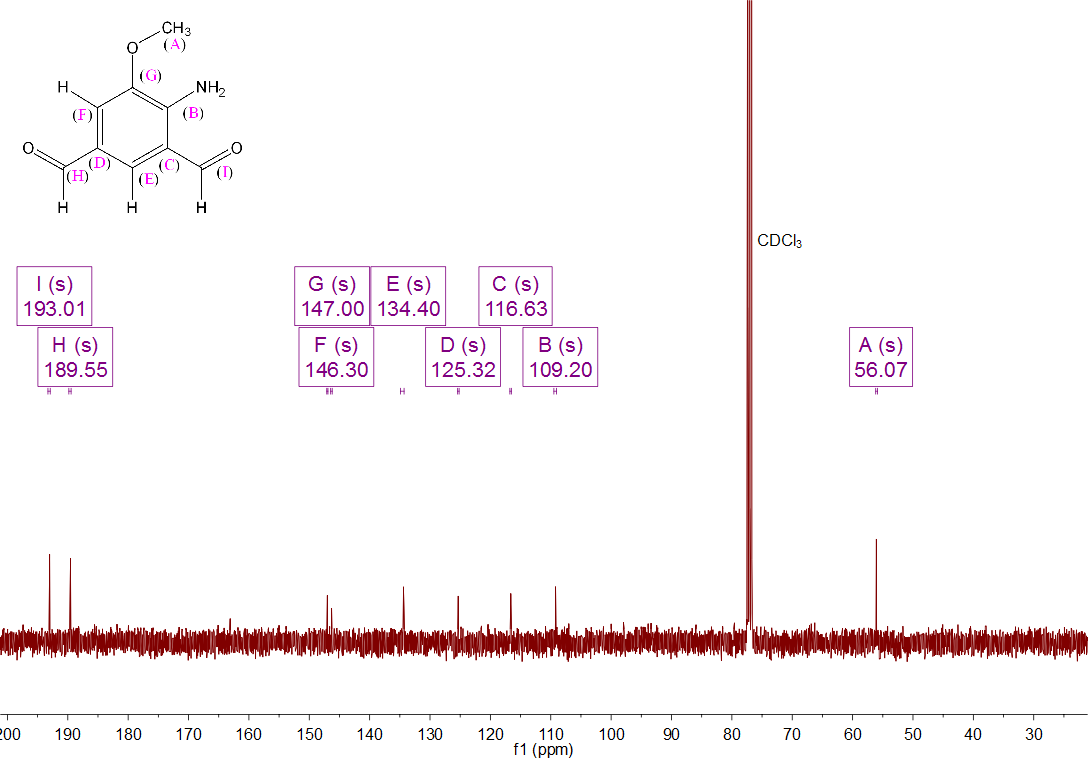


**^1^H NMR (400 MHz, CDCl_3_) and ^13^C NMR (101 MHz, CDCl_3_) of 4-amino-5-(methylthio)-isophthalaldehyde (3c)**


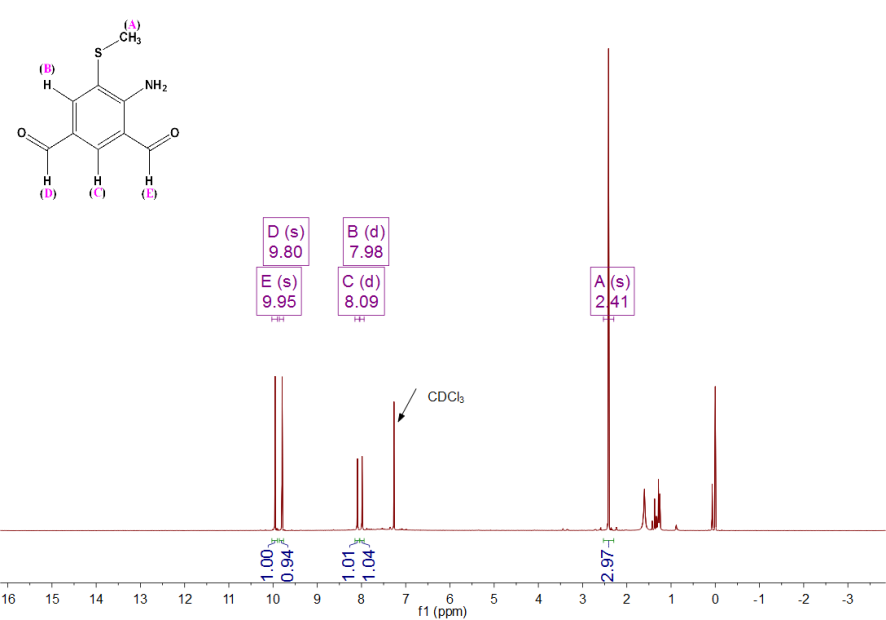

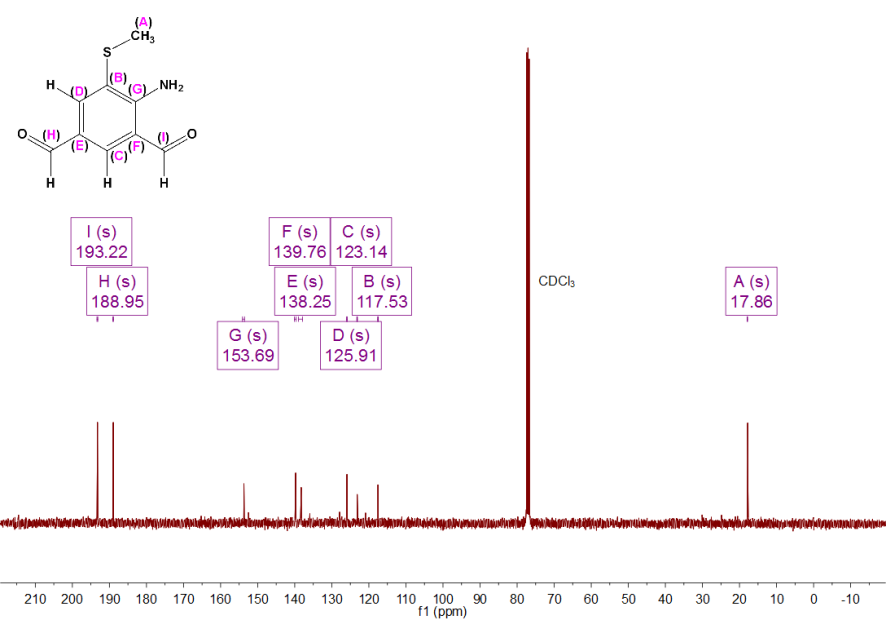


**^1^H NMR (400 MHz, CDCl_3_) and ^13^C NMR (101 MHz, CDCl_3_) of 4-amino-5-phenoxyisophthal-aldehyde (3d)**


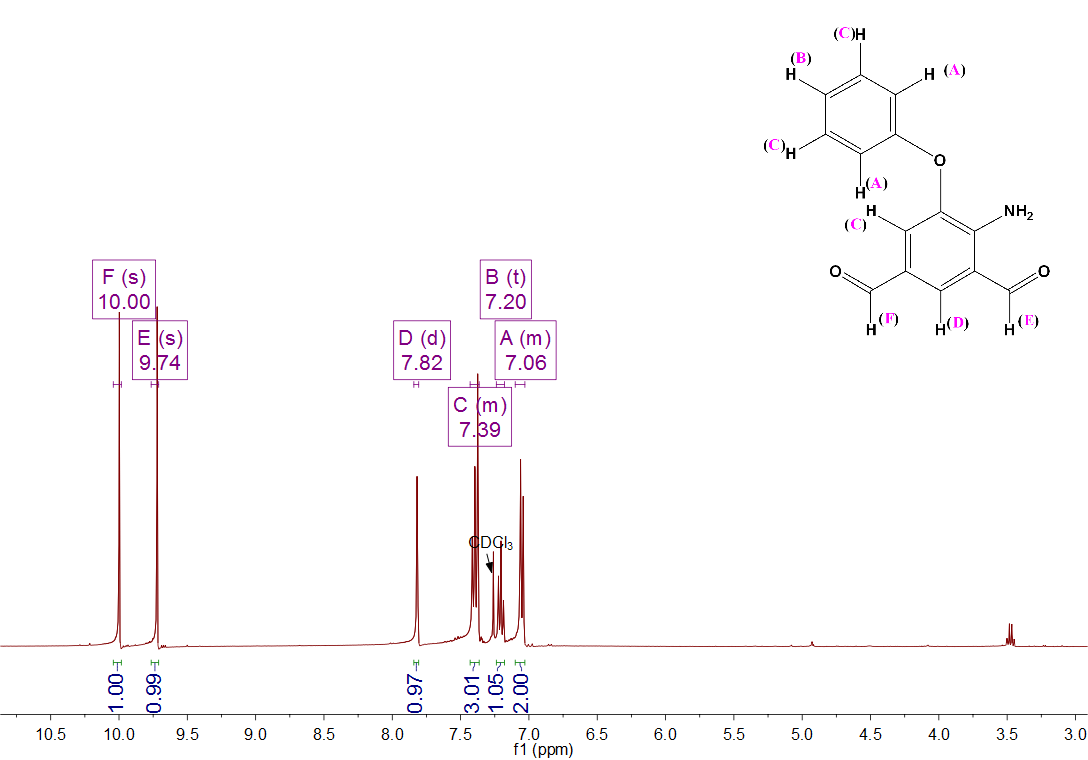


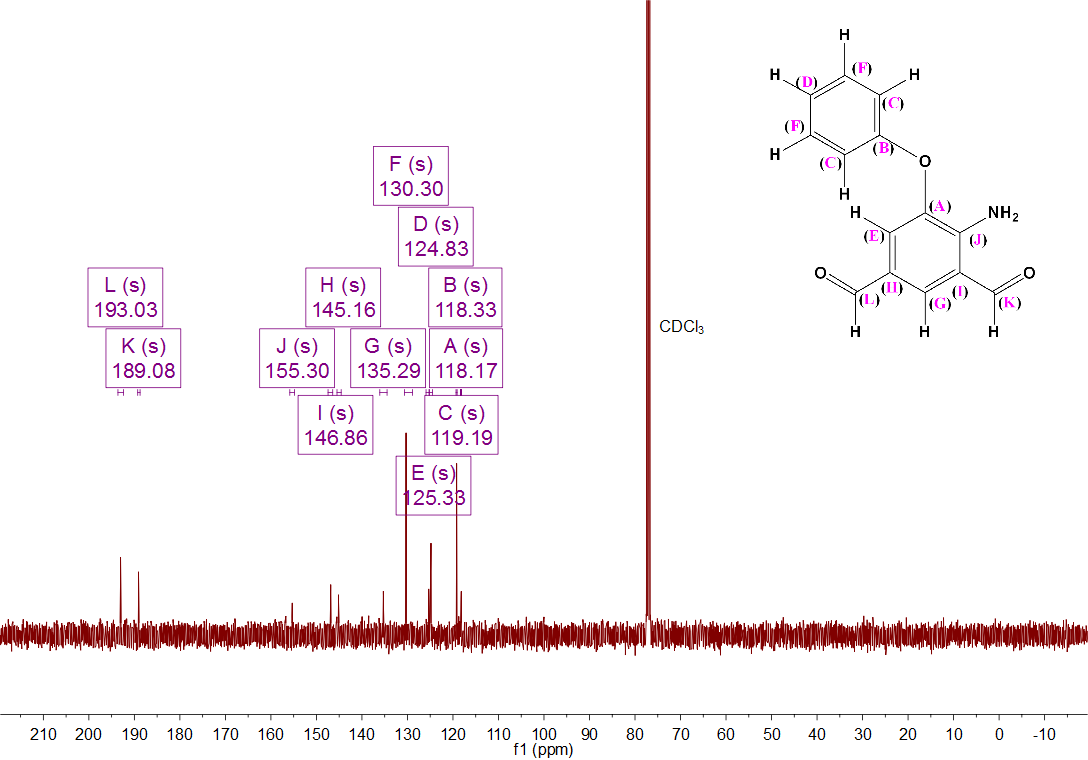


**^1^H NMR (400 MHz, CDCl_3_) and ^13^C NMR (101 MHz, CDCl_3_) of 4-amino-5-(benzyloxy)-isophthalaldehyde (3e)**


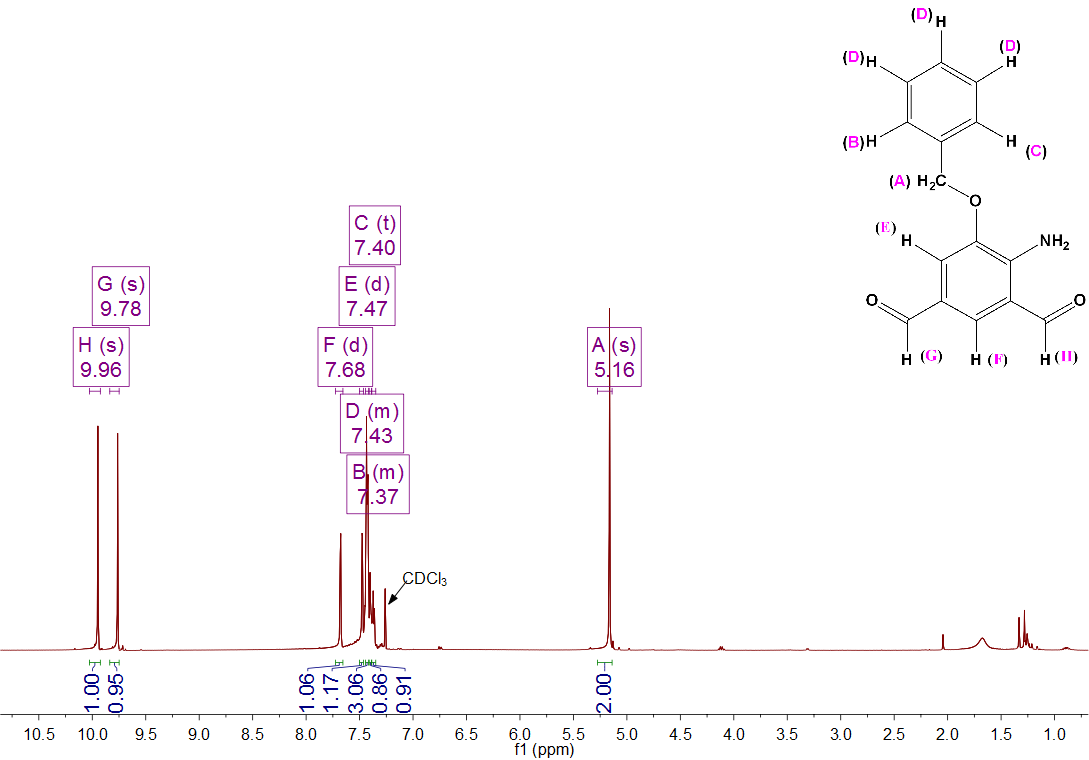


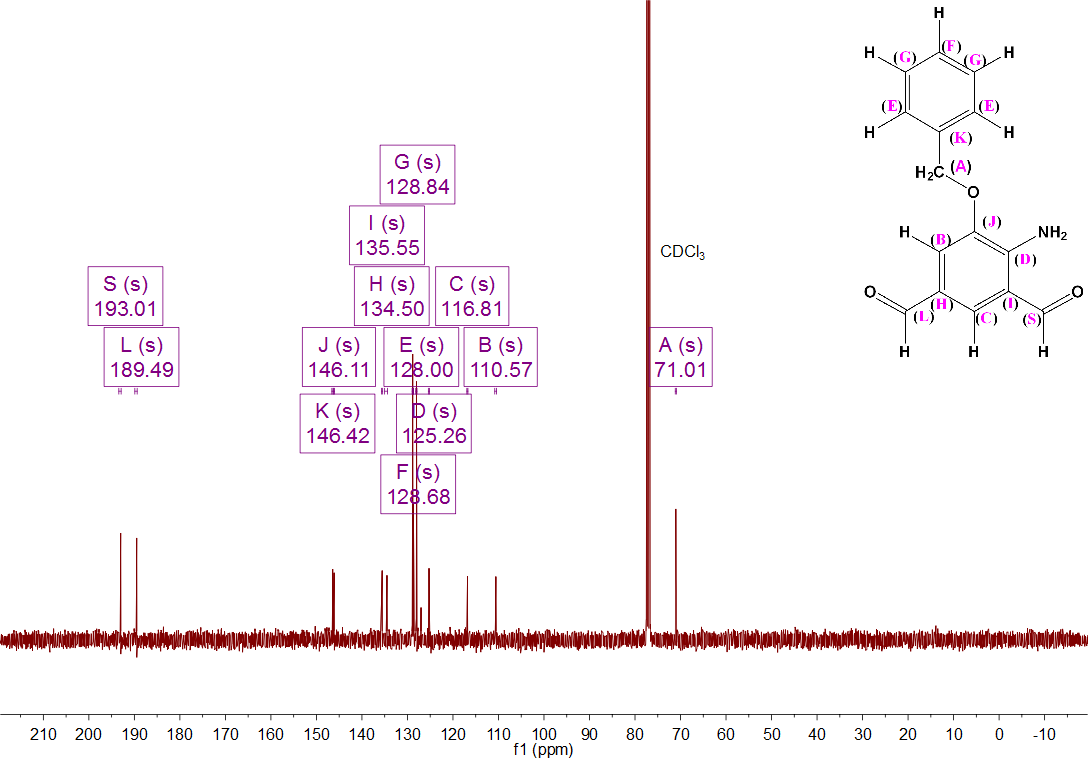


**^1^H NMR (400 MHz, CDCl_3_) and ^13^C NMR (101 MHz, CDCl_3_) of 4-amino-5-isopropylisophthal-aldehyde (3f)**


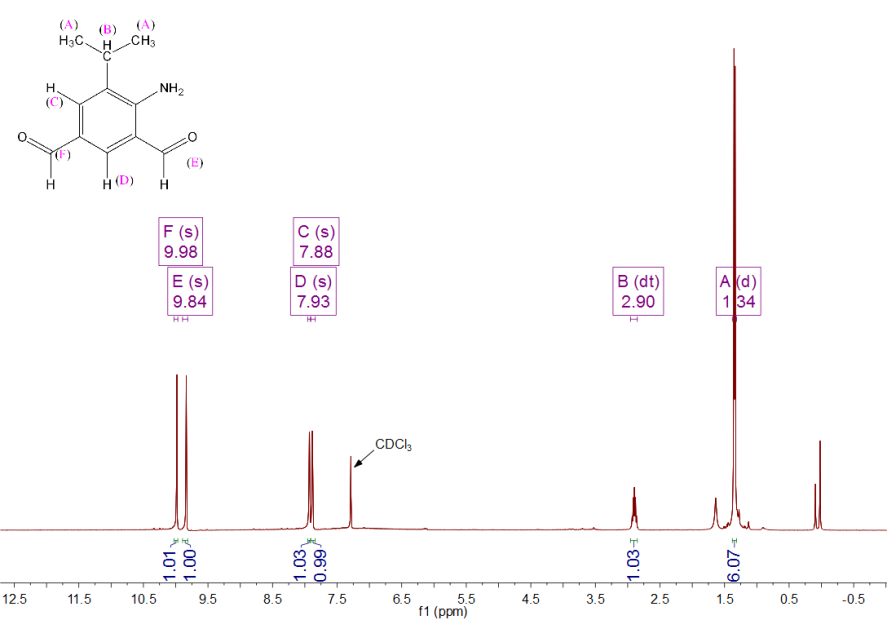


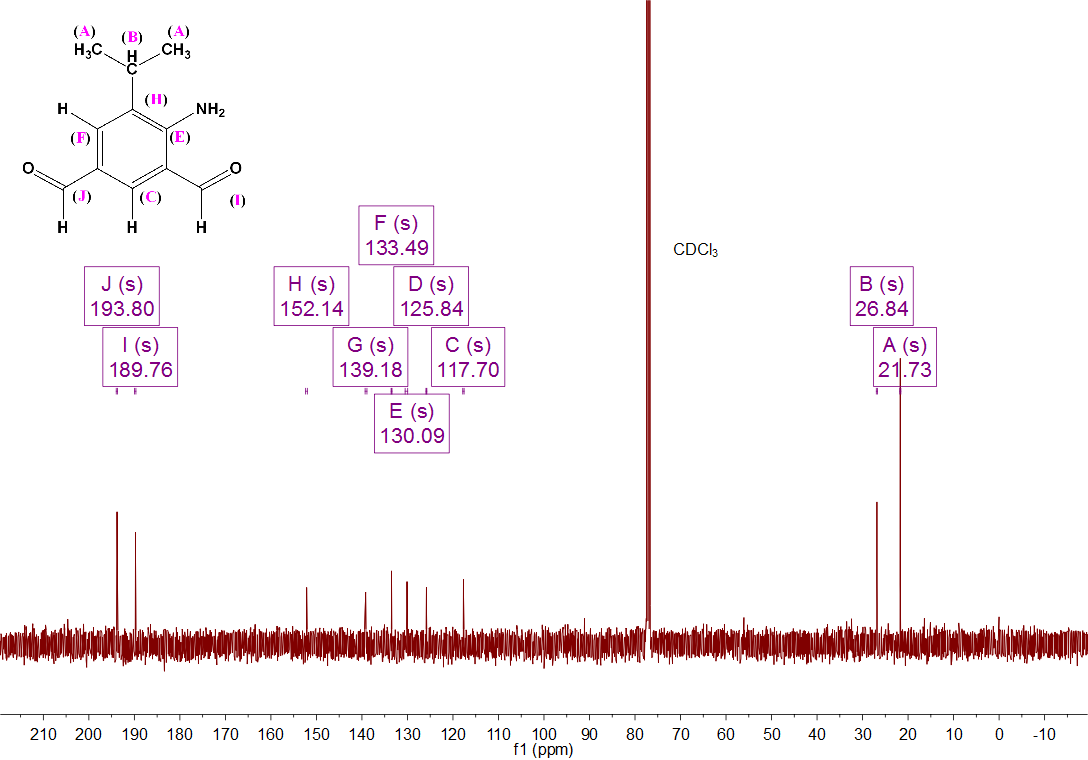


**^1^H NMR (400 MHz, CDCl_3_) and ^13^C NMR (101 MHz, CDCl_3_) of 4-amino-5-ethylisophthal-aldehyde (3g)**


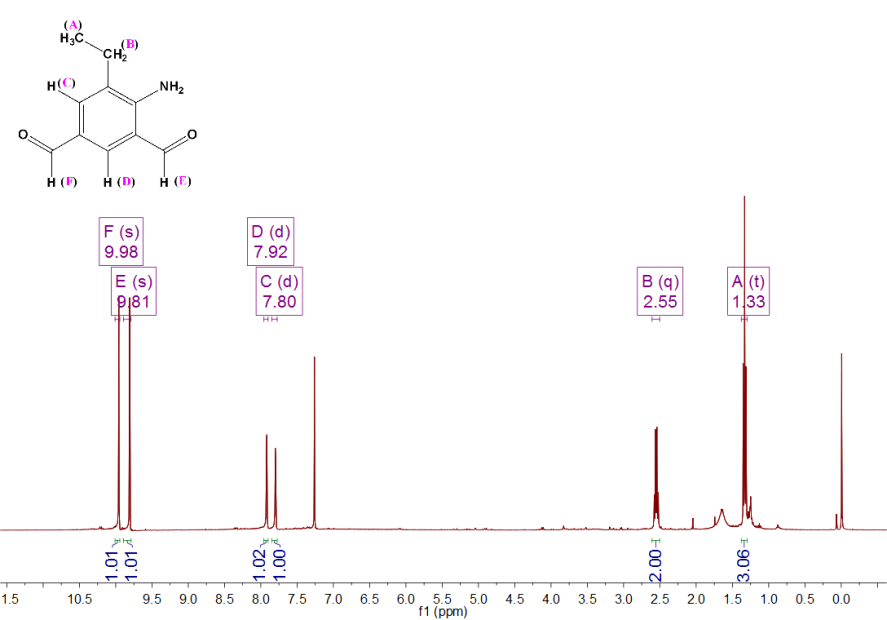


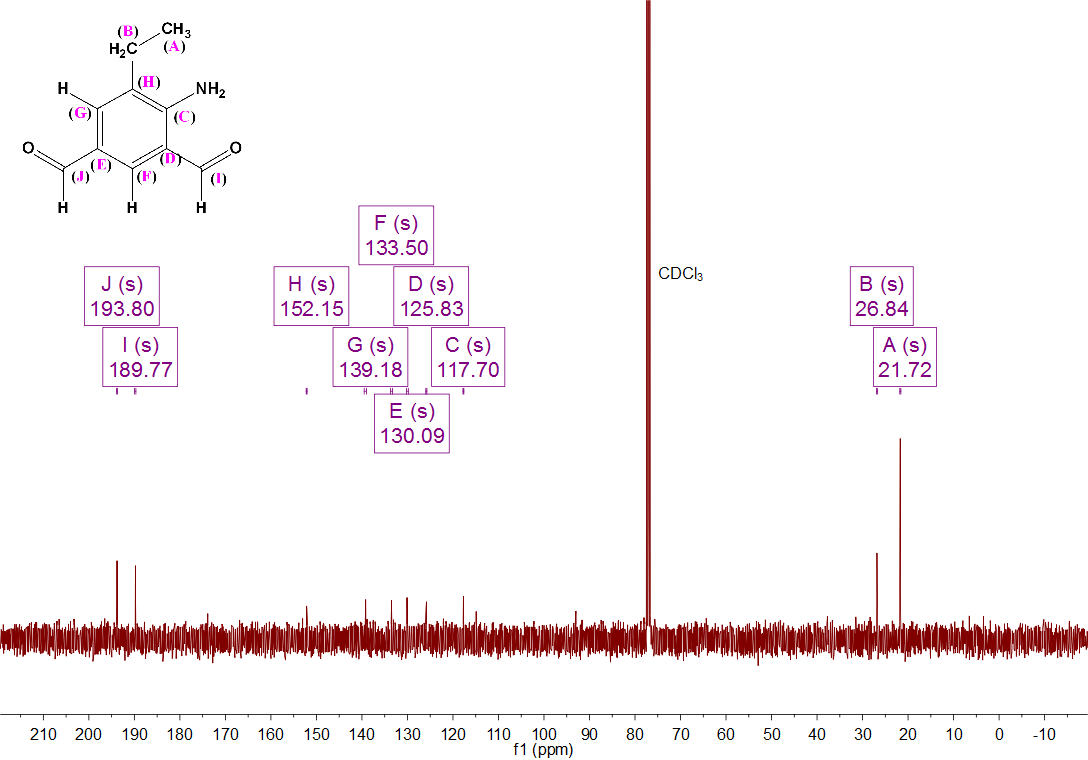


**^1^H NMR (400 MHz, CDCl_3_) and ^13^C NMR (101 MHz, CDCl_3_) of 4-amino-5-methylisophthal-aldehyde (3h)**


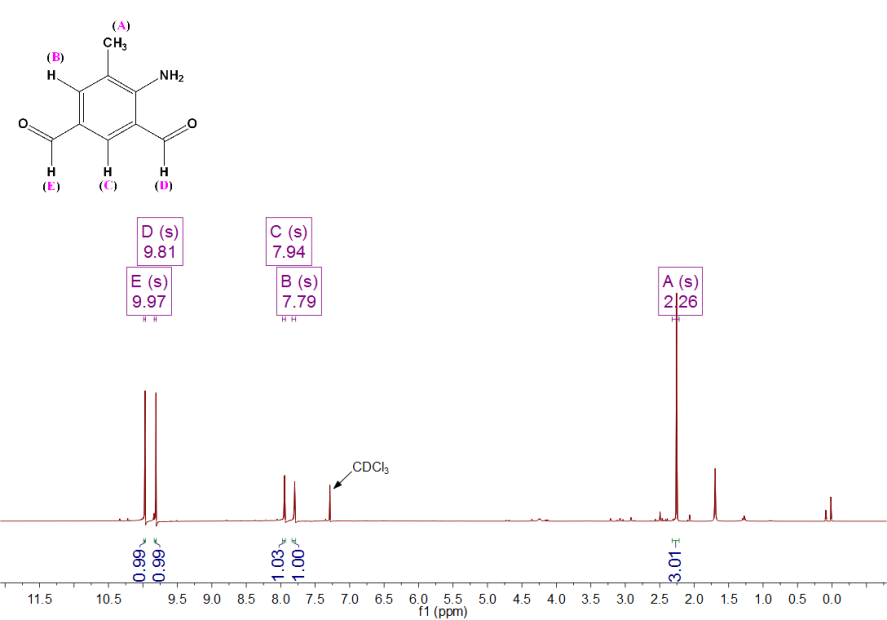


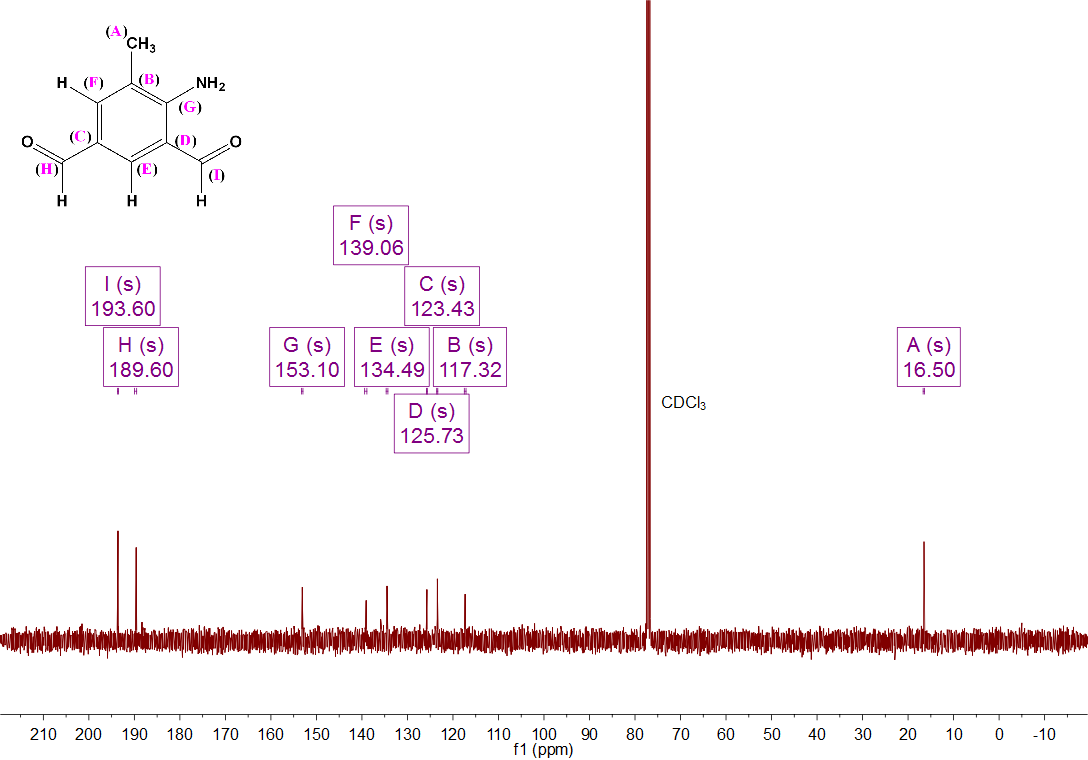


**^1^H NMR (400 MHz, CDCl_3_) and ^13^C NMR (101 MHz, CDCl_3_) of 2-amino-[1,1'-biphenyl]-3,5-dicarbaldehyde (3i)**


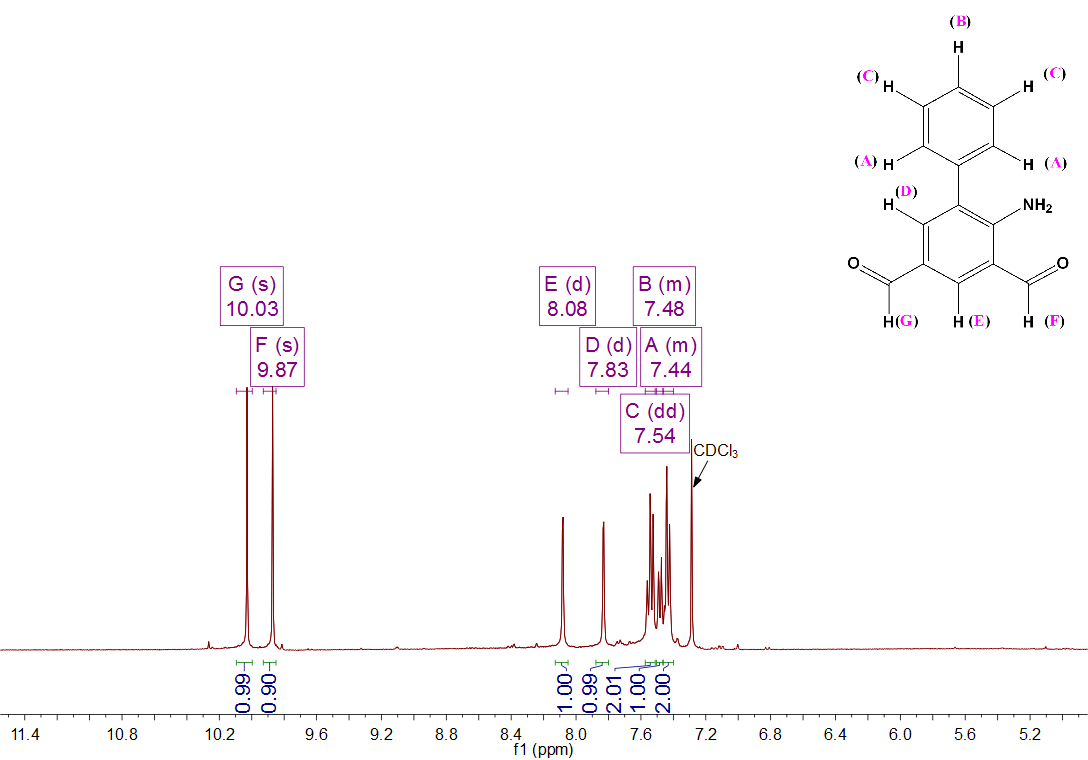


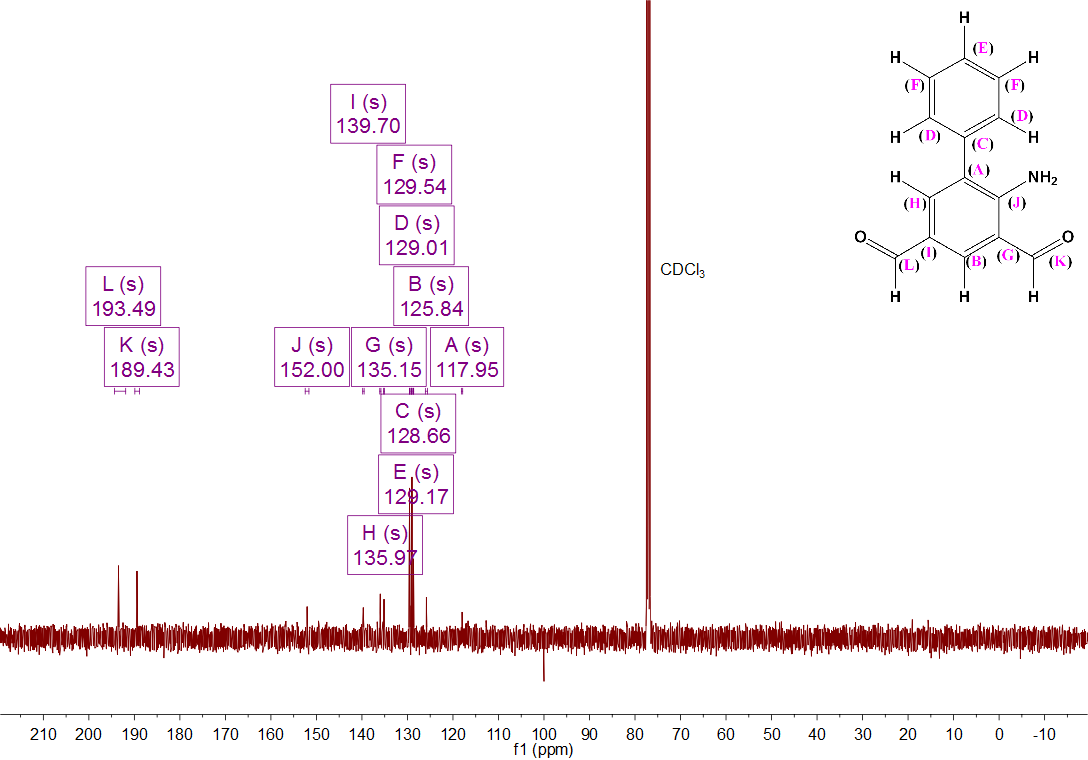


**^1^H NMR (400 MHz, CDCl_3_) and ^13^C NMR (101 MHz, CDCl_3_) of 4-amino-5-bromoisophthal-aldehyde (3j)**


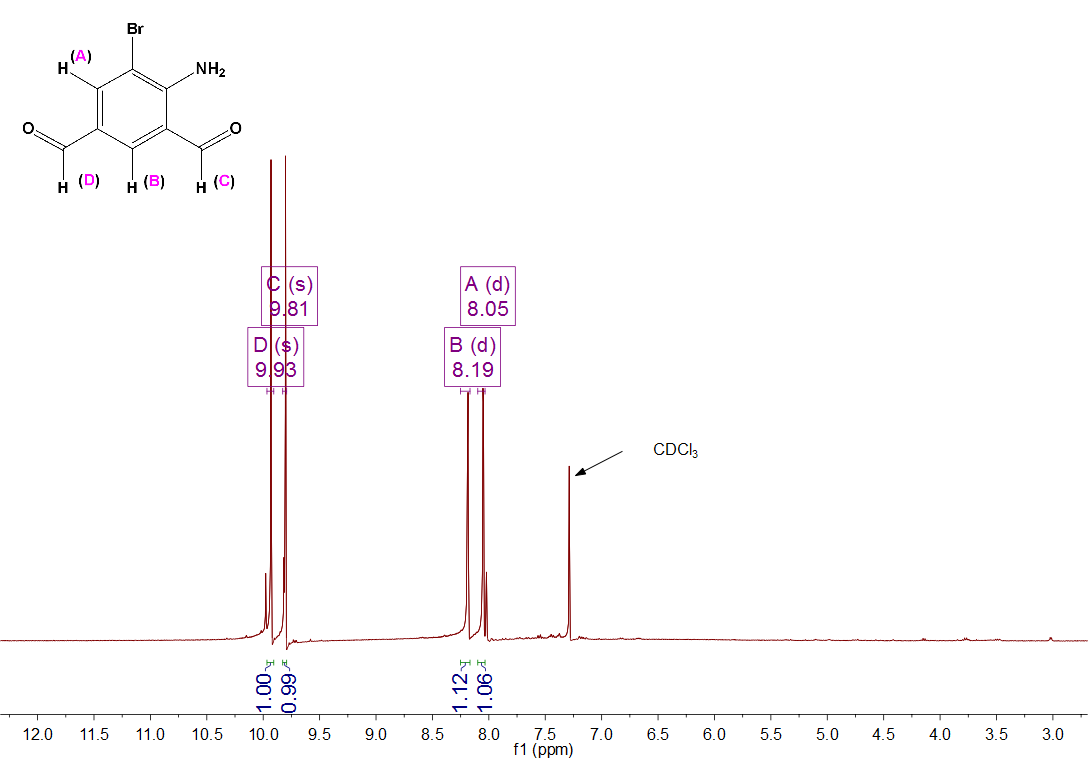


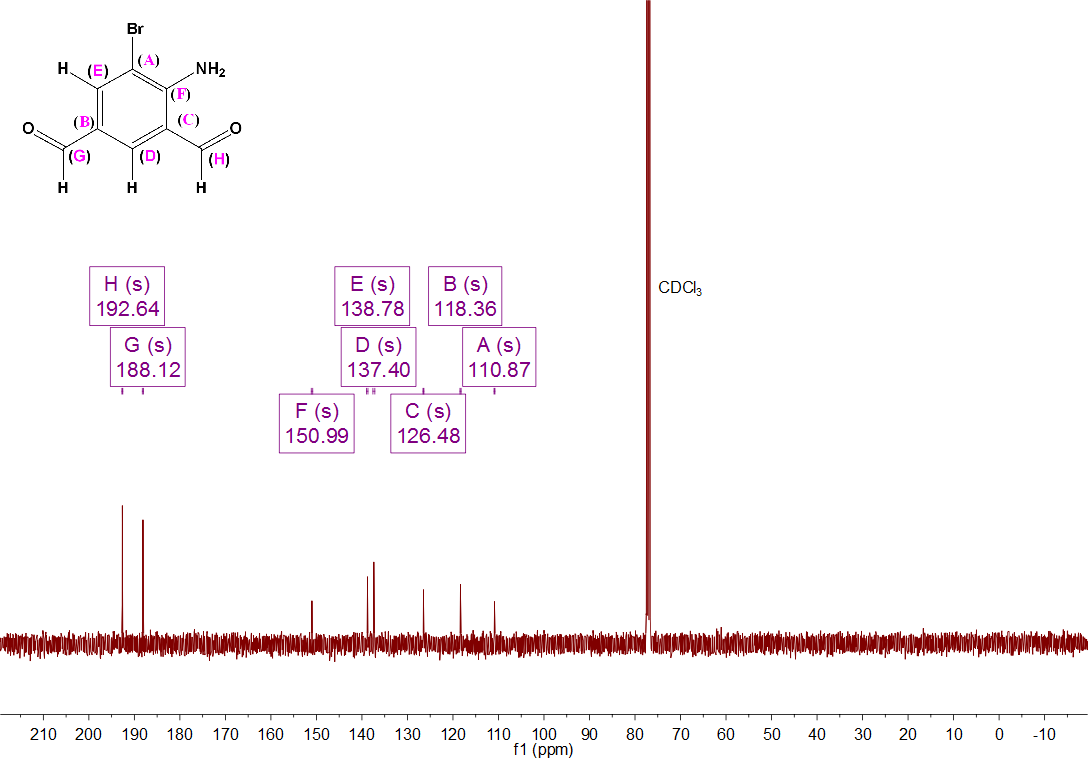


**^1^H NMR (400 MHz, CDCl_3_) and ^13^C NMR (101 MHz, CDCl_3_) of 4-amino-5-chloroisophthal-aldehyde (3k)**


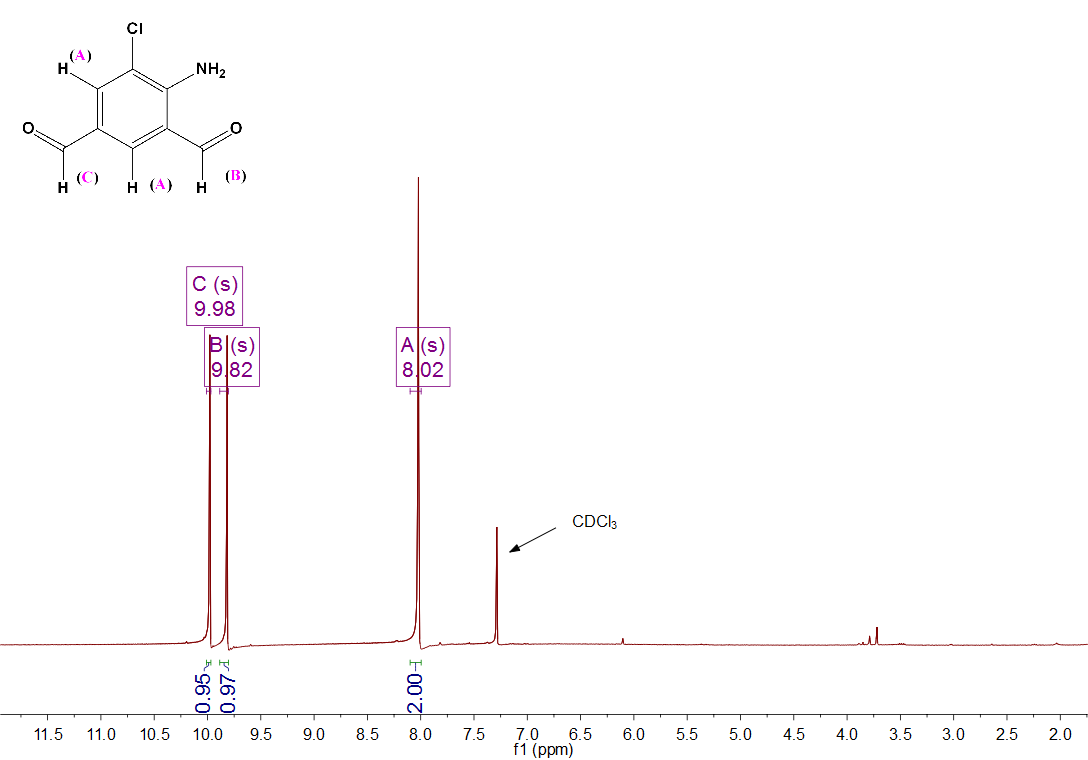


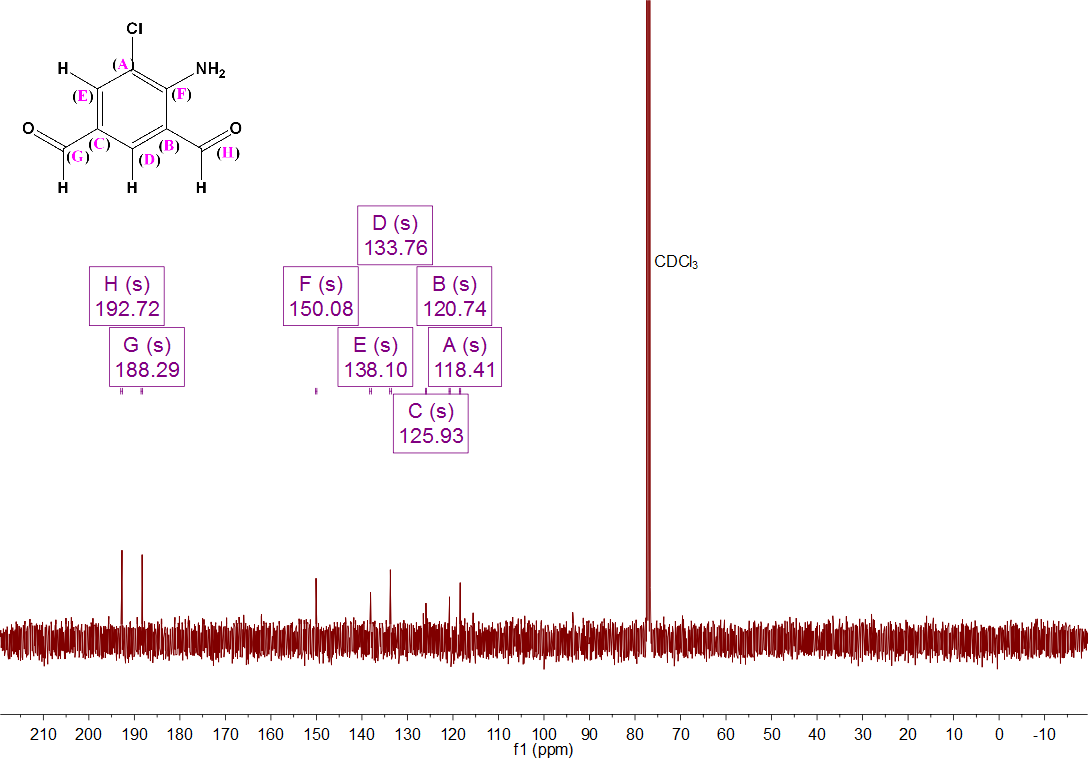


**^1^H NMR (400 MHz, CDCl_3_) and ^13^C NMR (101 MHz, CDCl_3_) of 4-amino-5-fluoroisophthal-aldehyde (3l)**


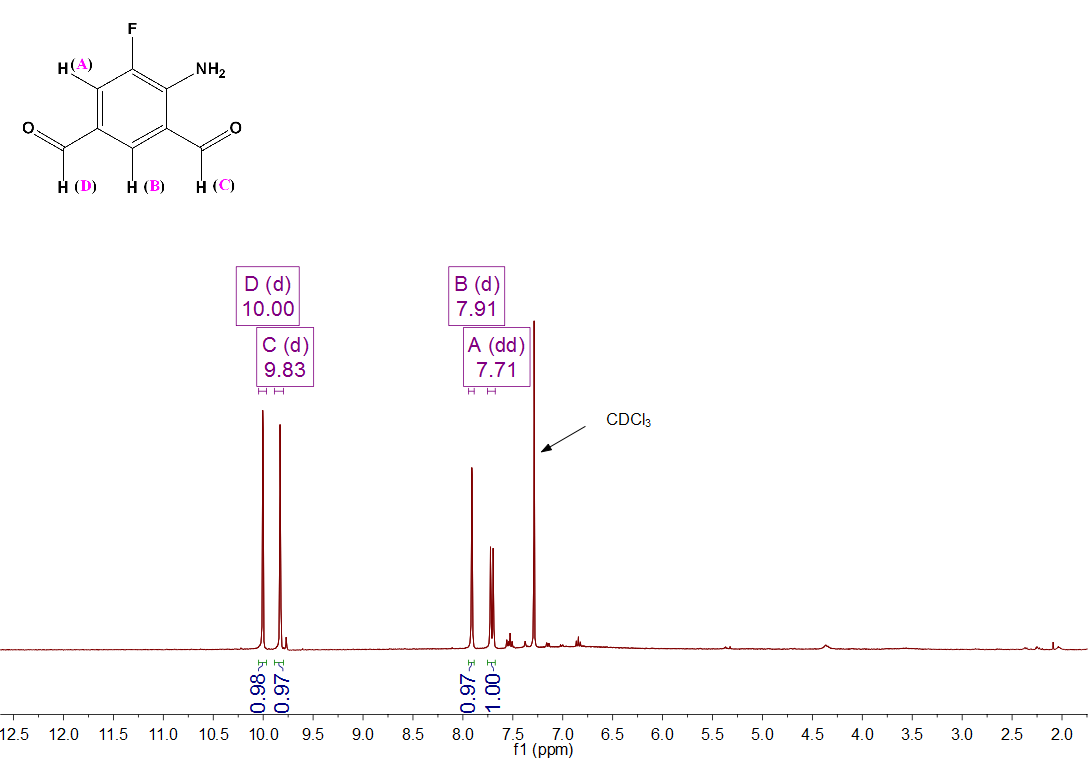


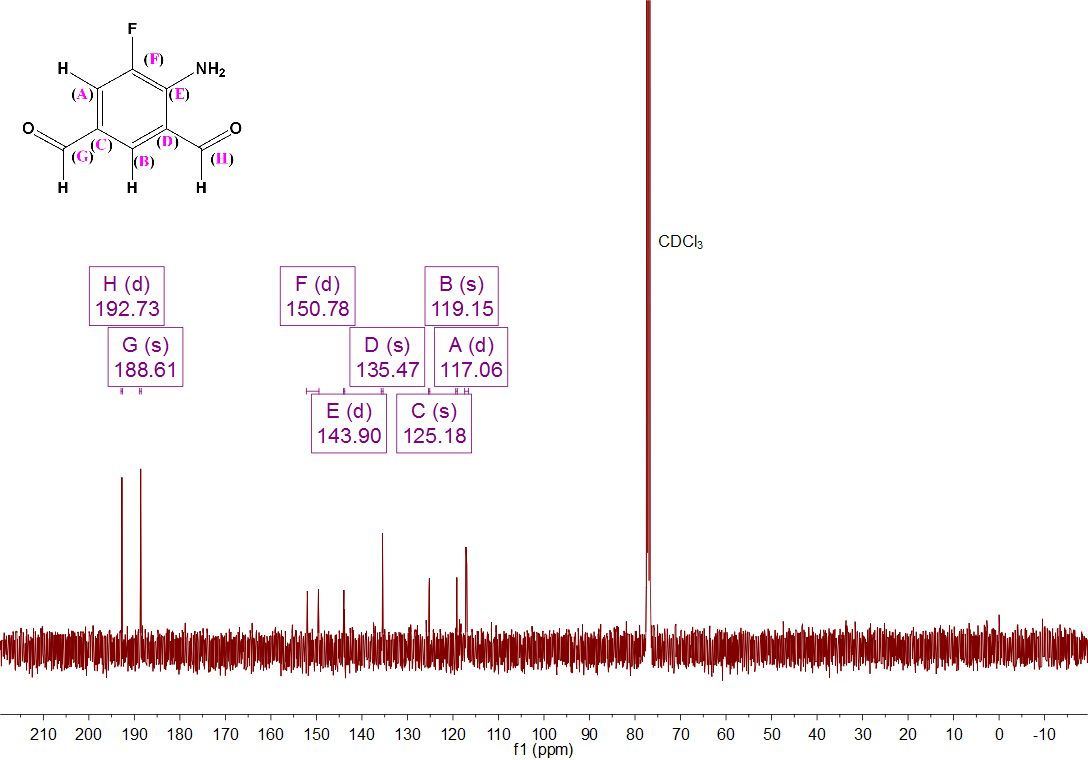


**^1^H NMR (400 MHz, CDCl_3_) and ^13^C NMR (101 MHz, CDCl_3_) of 4-methoxy-6-(methylamino)-isophthalaldehyde (3m)**


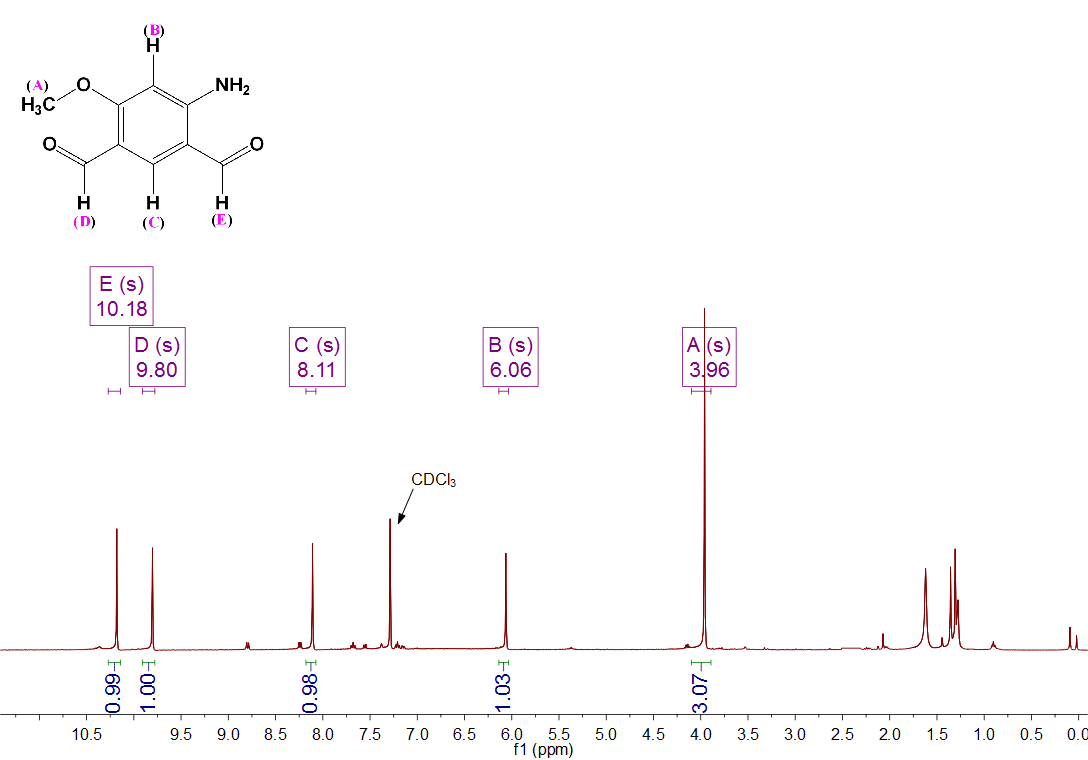


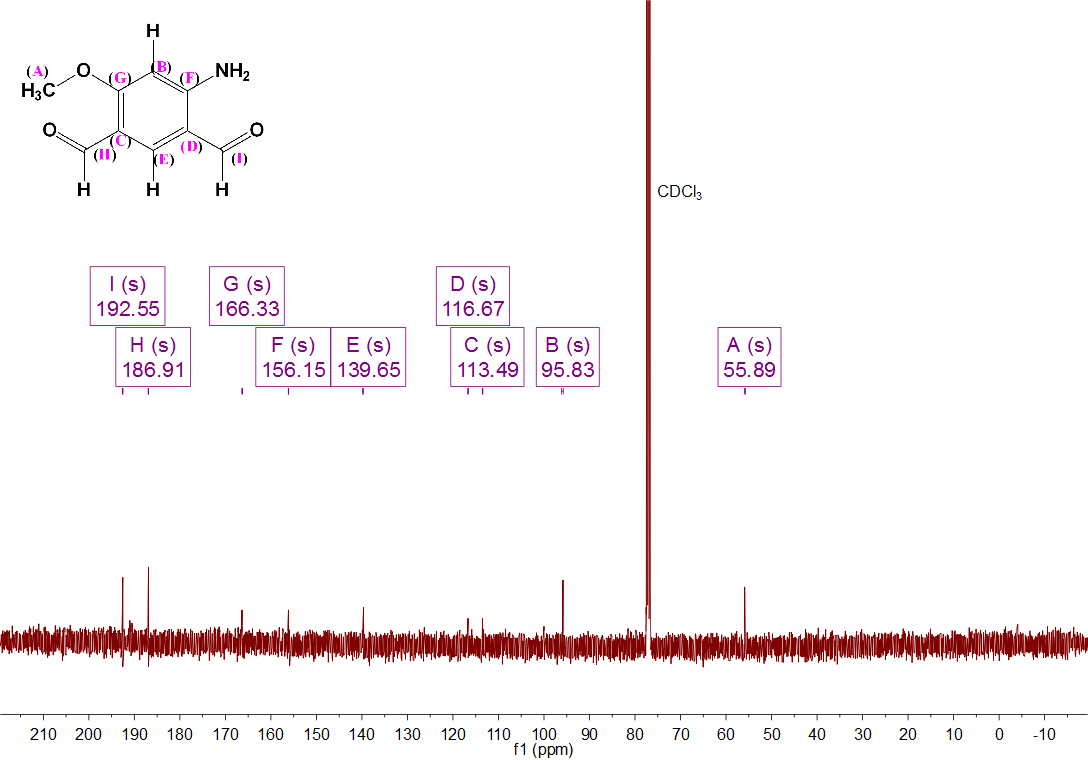


**^1^H NMR (400 MHz, CDCl_3_) and ^13^C NMR (101 MHz, CDCl_3_) of 4-amino-6-isopropylisophthal-aldehyde (3n)**


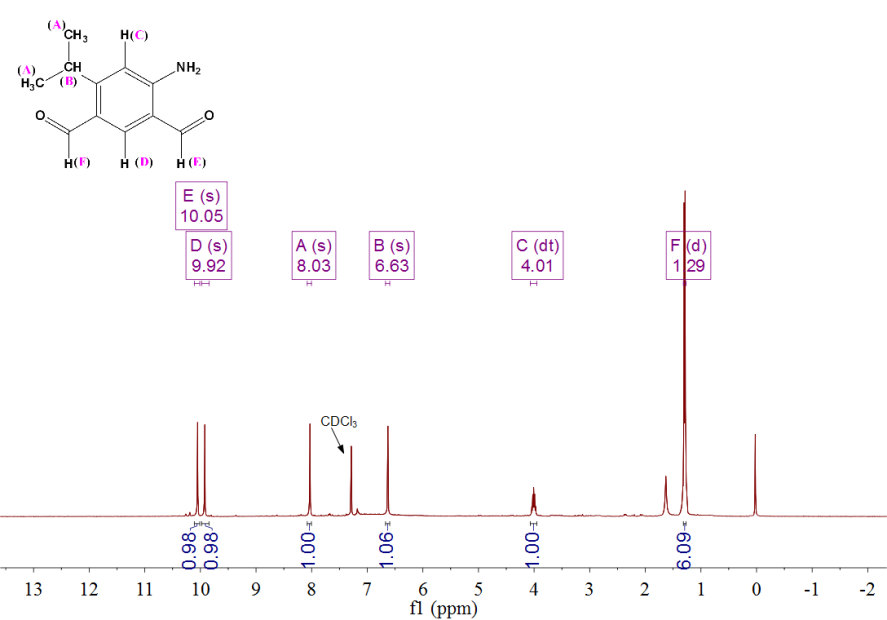


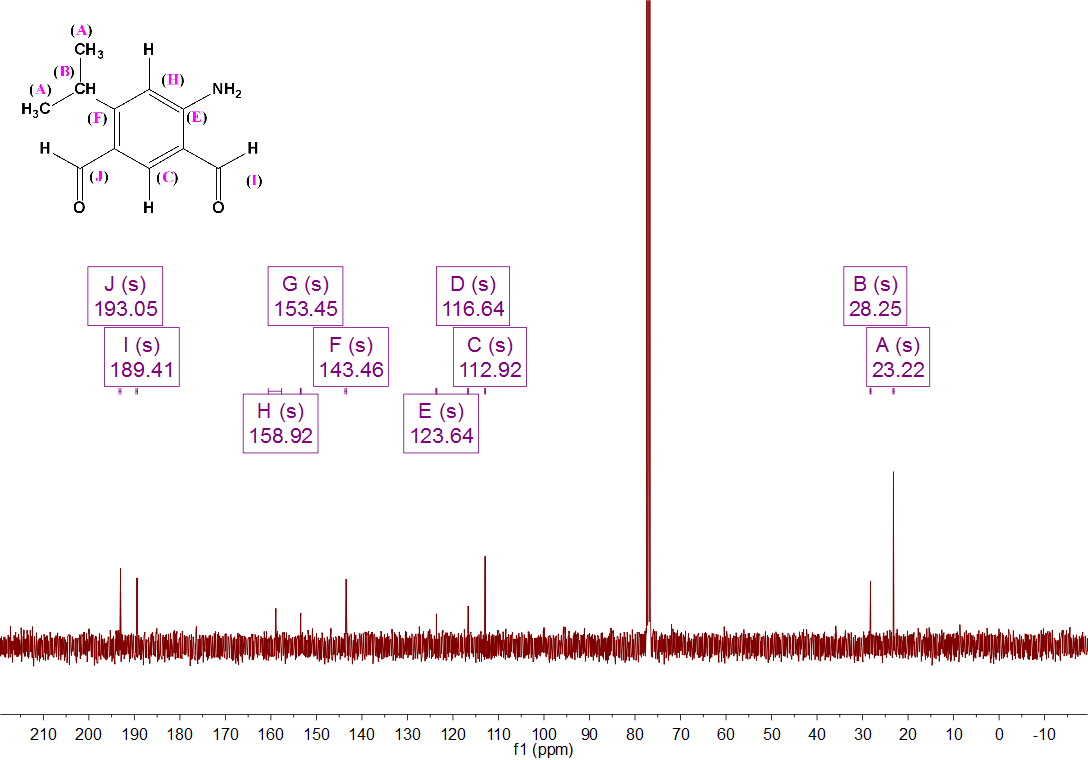


**^1^H NMR (400 MHz, CDCl_3_) and ^13^C NMR (101 MHz, CDCl_3_) of 4-amino-5-ethylisophthal-aldehyde (3o)**


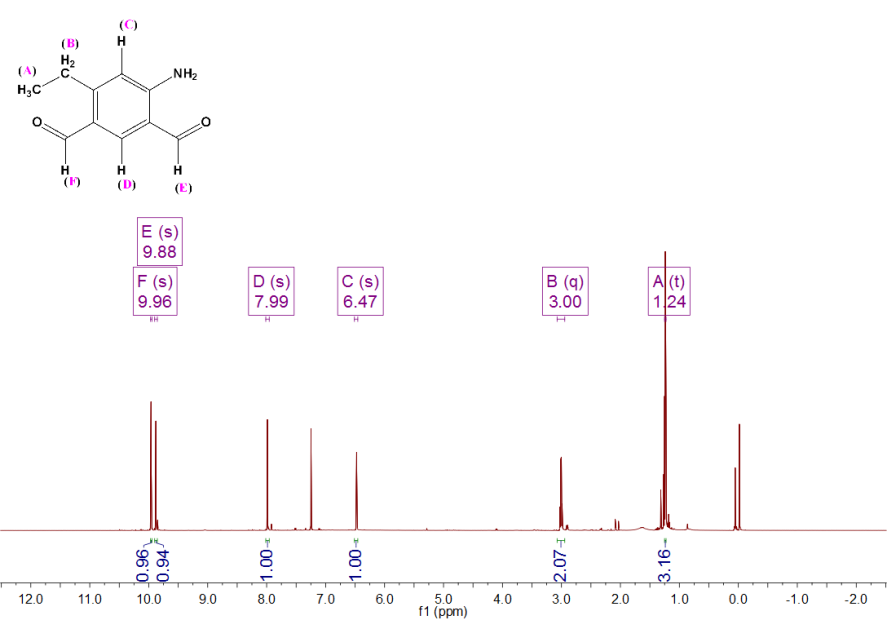


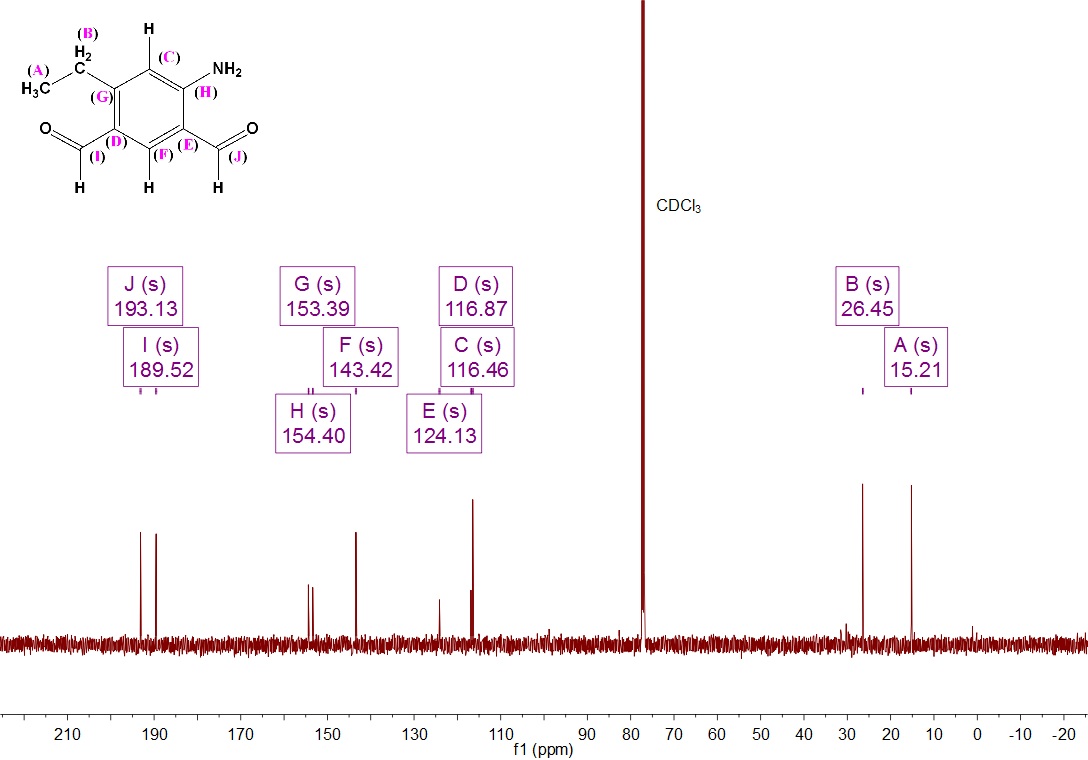


**^1^H NMR (400 MHz, CDCl_3_) and ^13^C NMR (101 MHz, CDCl_3_) of 4-amino-6-methylisophthal-aldehyde (3p)**


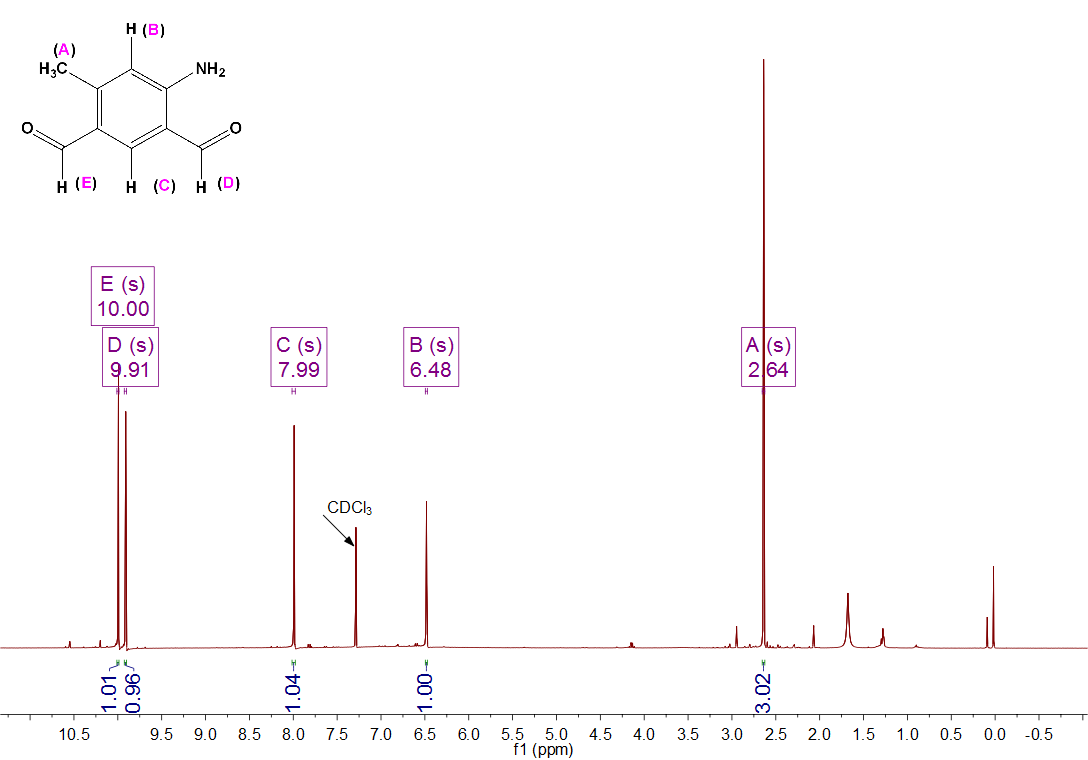


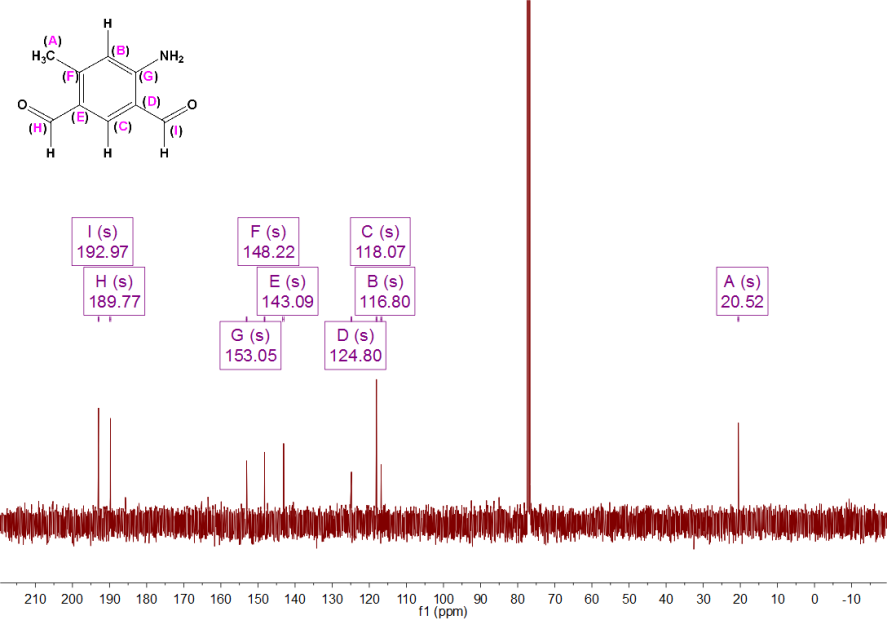


**^1^H NMR (400 MHz, CDCl_3_) and ^13^C NMR (101 MHz, CDCl_3_) of 4-amino-6-bromoisophthal-aldehyde (3q) ^
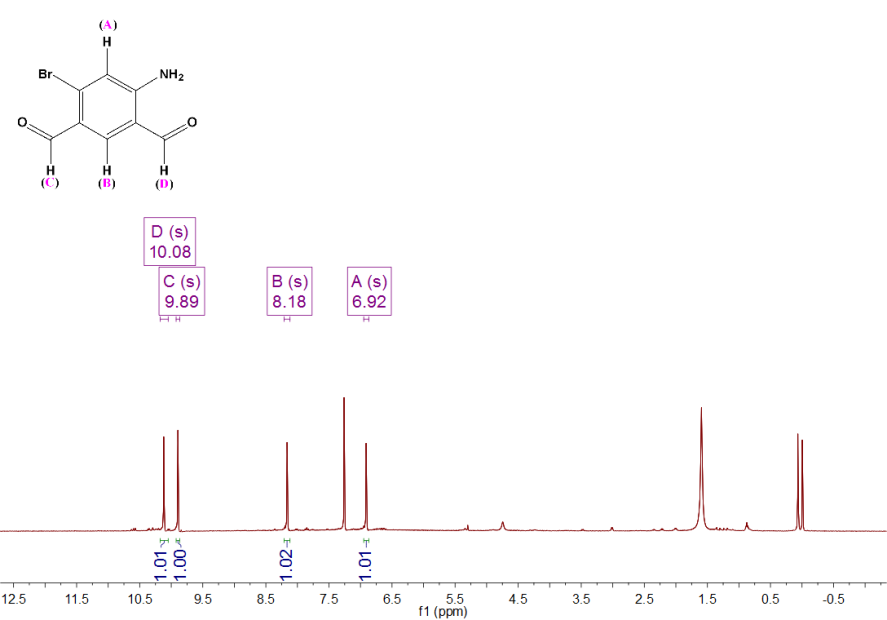
^**


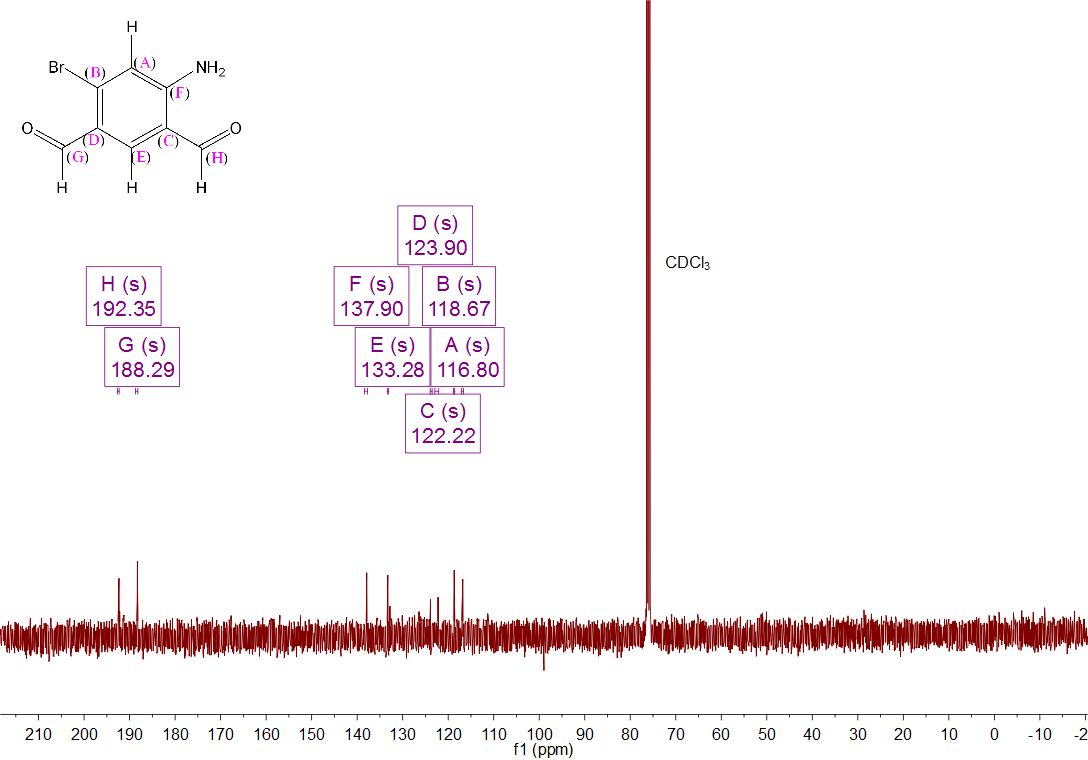


**^1^H NMR (400 MHz, CDCl_3_) and ^13^C NMR (101 MHz, CDCl_3_) of 4-amino-6-chloroisophthal-aldehyde (3r)**


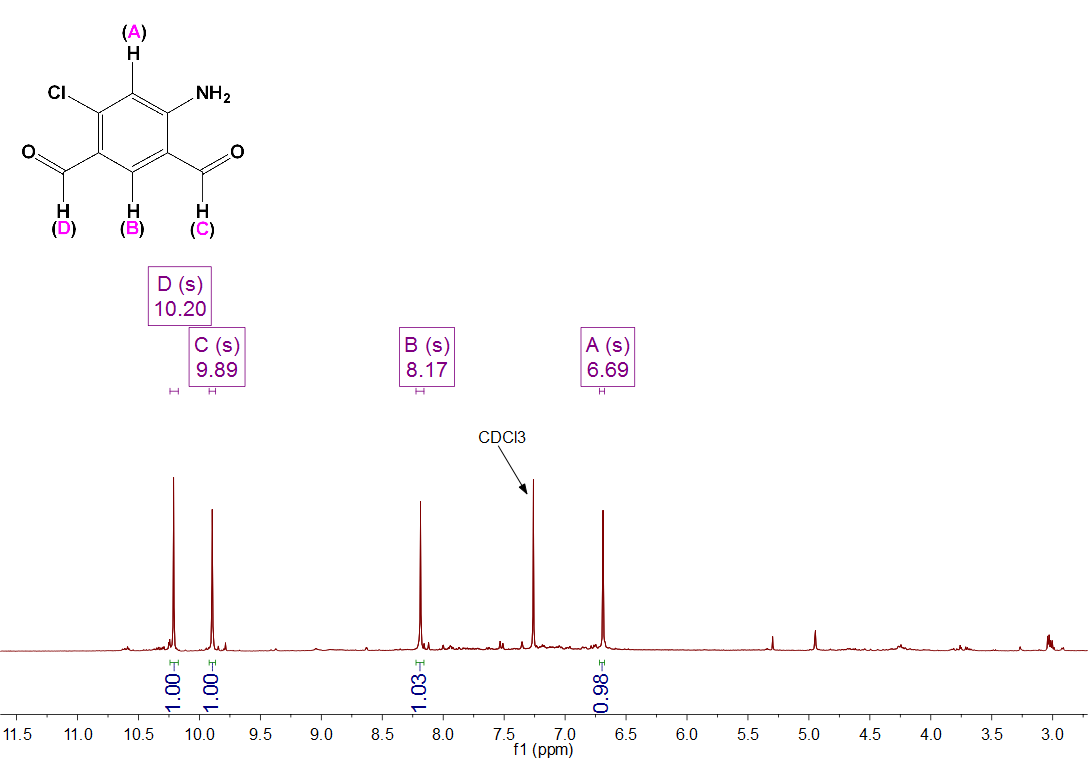


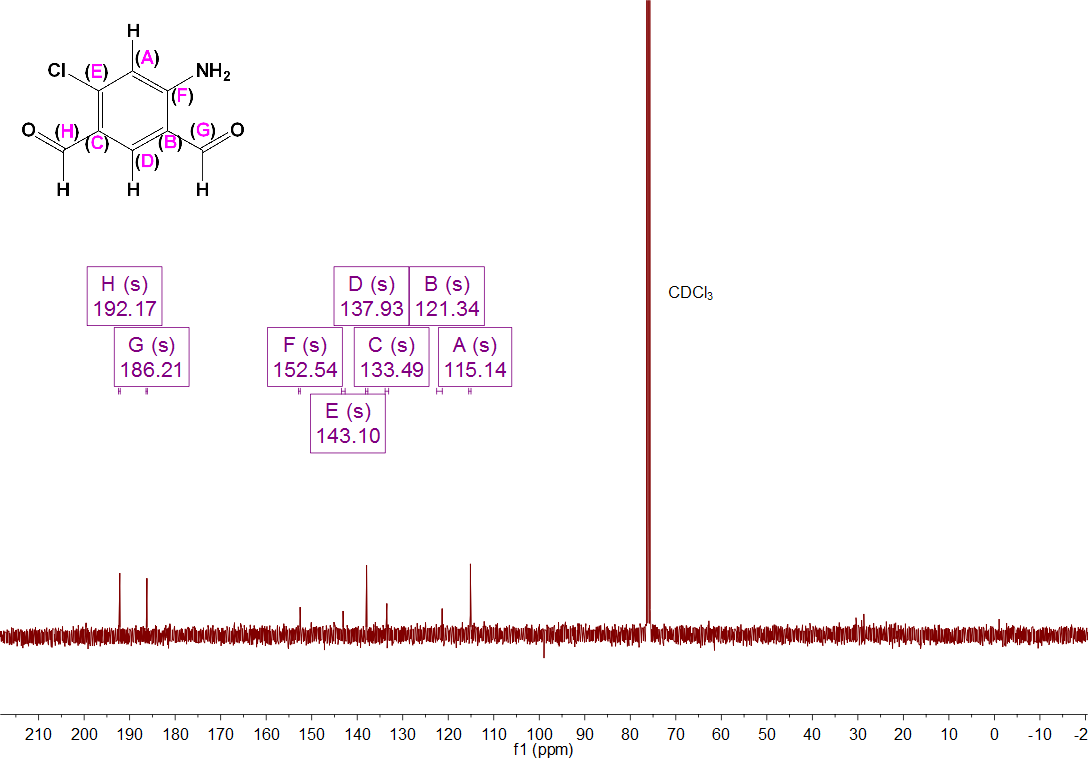


**^1^H NMR (400 MHz, CDCl_3_) and ^13^C NMR (101 MHz, CDCl_3_) of 4-amino-6-fluoroisophthal-aldehyde (3s)**


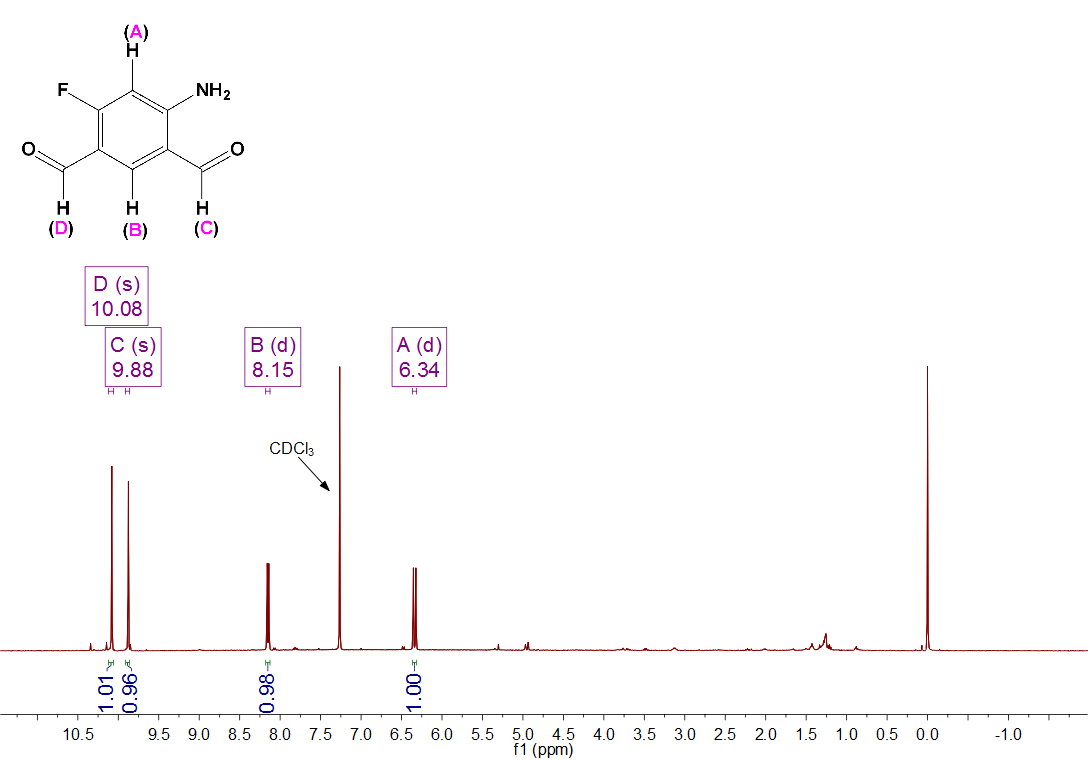

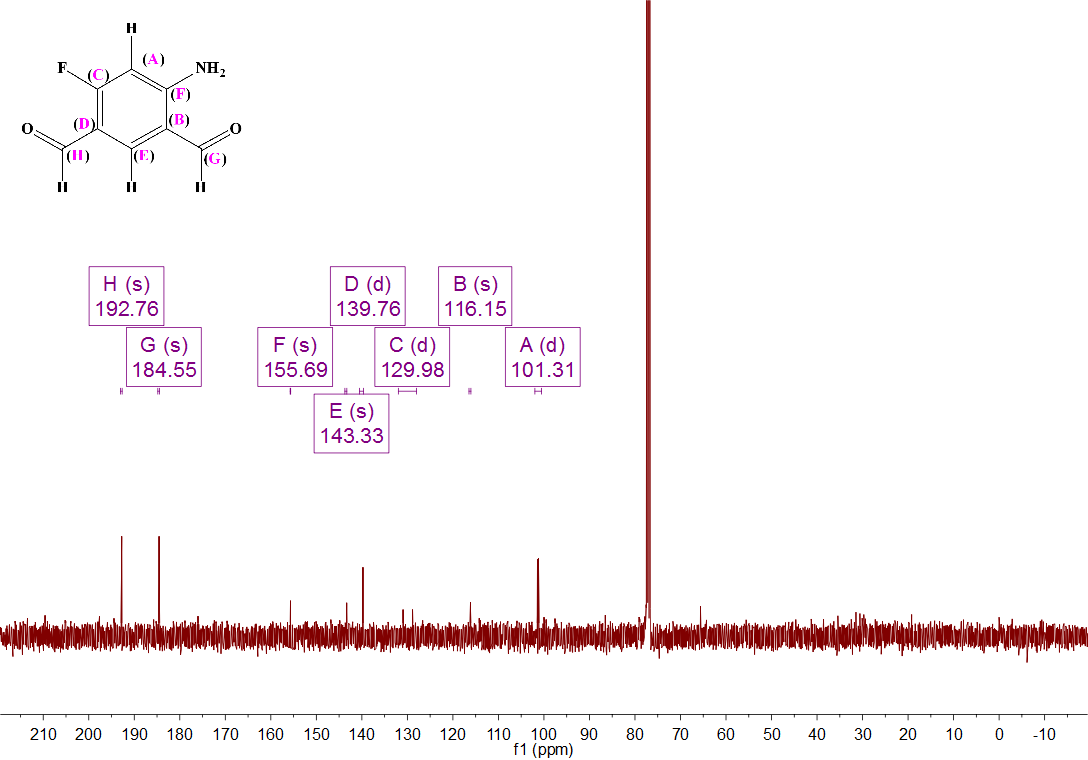


**^1^H NMR (400 MHz, CDCl_3_) and ^13^C NMR (101 MHz, CDCl_3_) of 4-amino-5, 6-dimethyl-isophthalaldehyde (3t)**

**
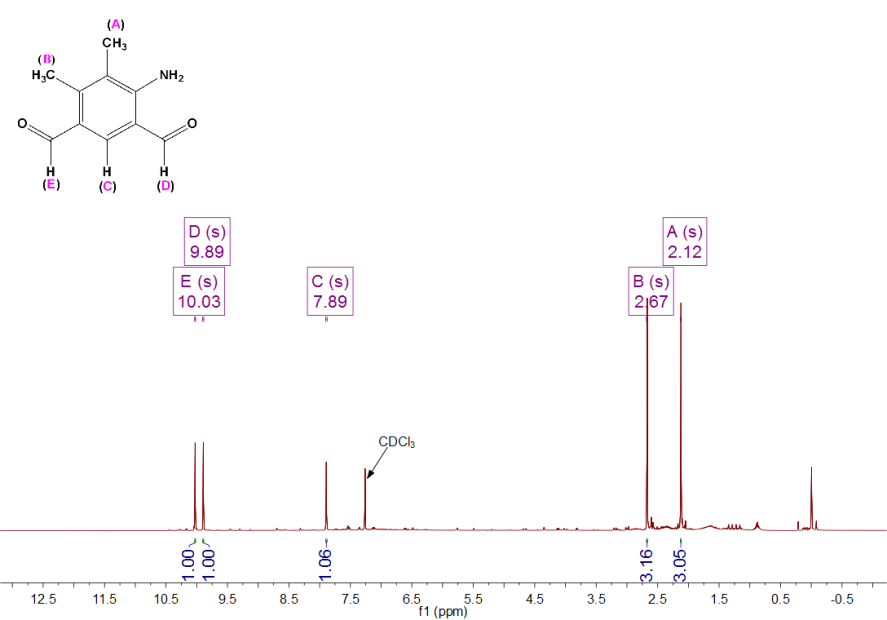
**

**
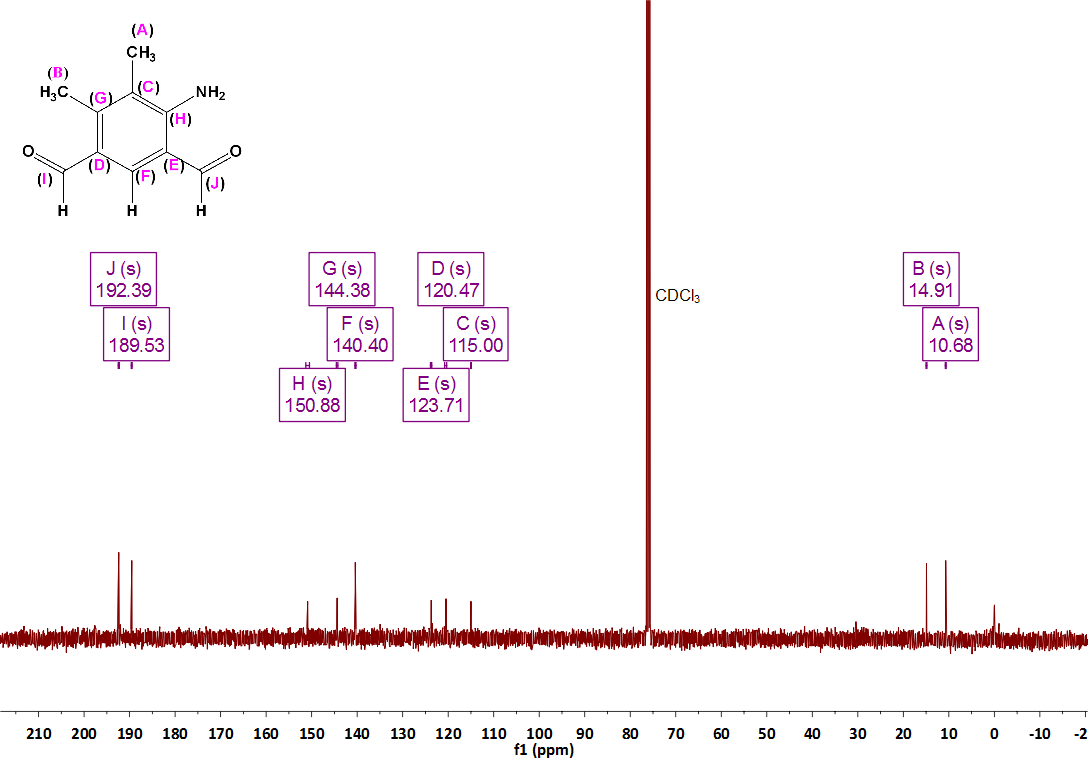
**

**^1^H NMR (400 MHz, CDCl_3_) and ^13^C NMR (101 MHz, CDCl_3_) of 7-amino-2,3-dihydro-1H-indene-4,6-dicarbaldehyde (3u)
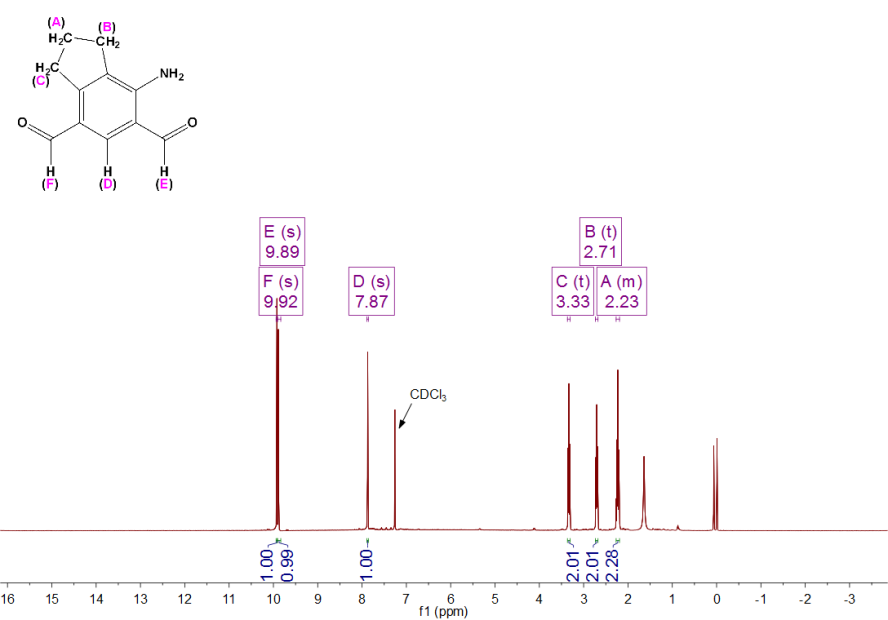

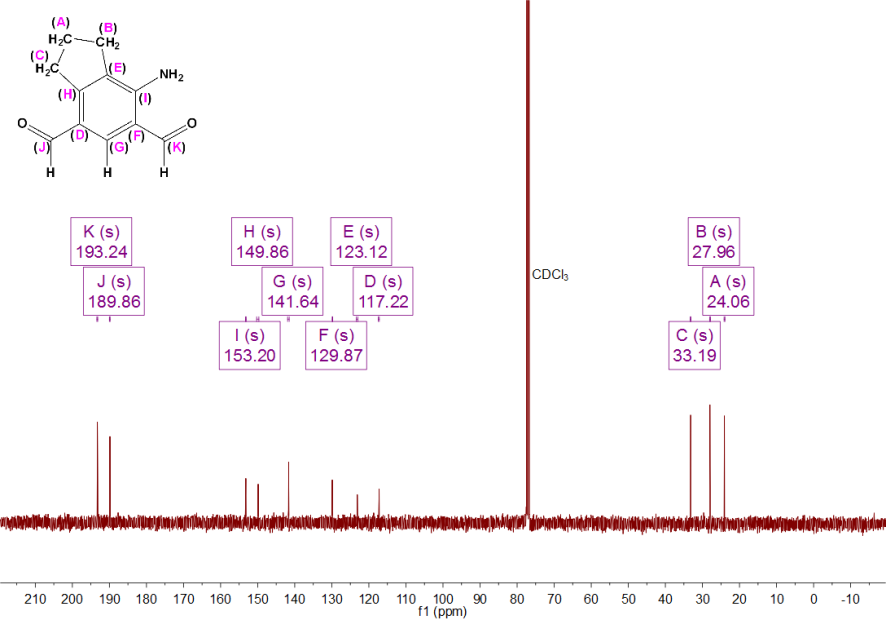
**

**^1^H NMR (400 MHz, CDCl_3_) and ^13^C NMR (101 MHz, CDCl_3_) of 4-aminonaphthalene-1,3-dicarbaldehyde (3v)**

**
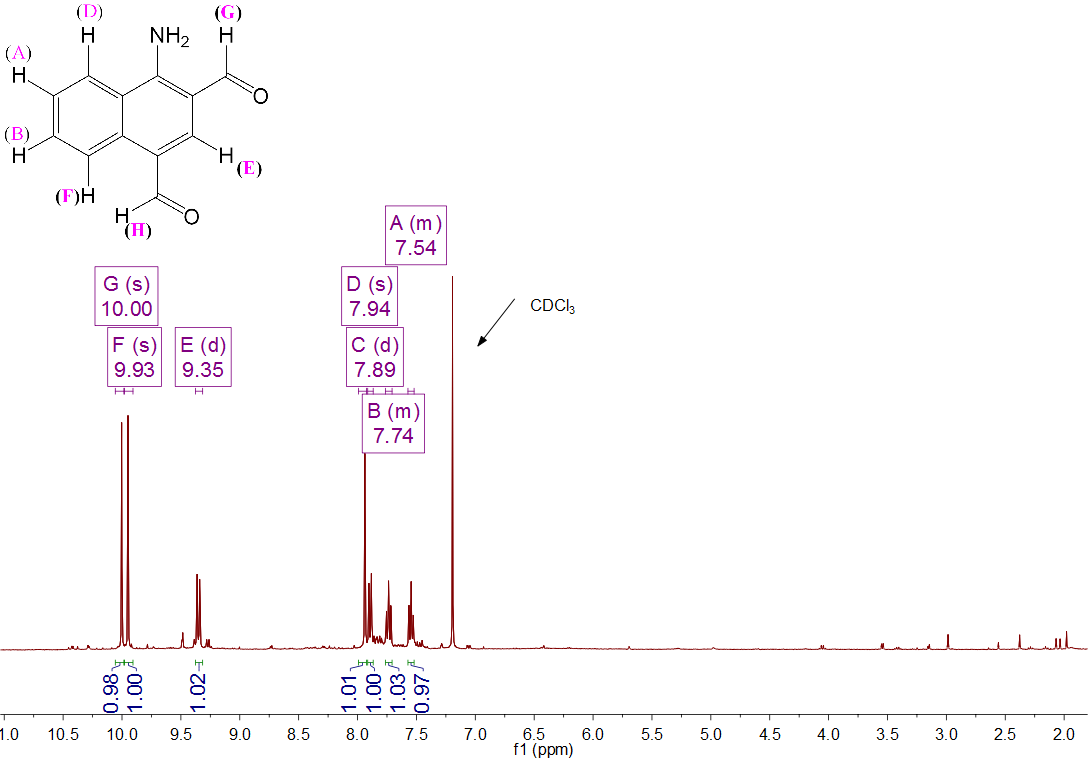
**

**
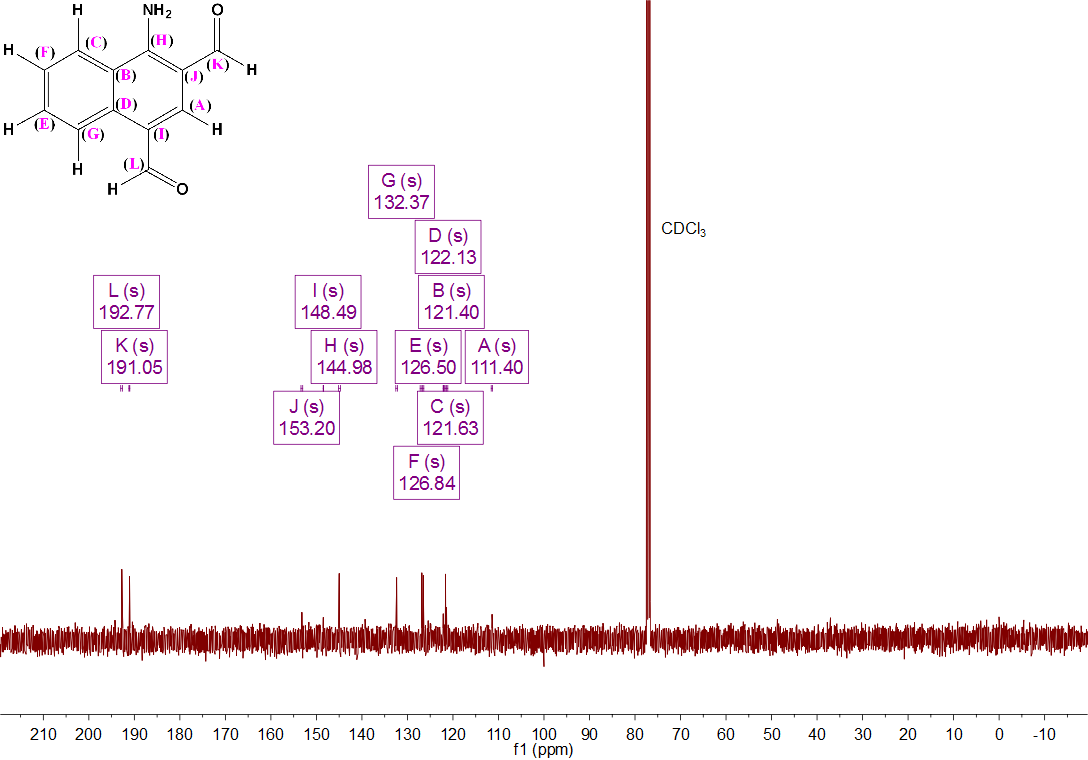
**

**^1^H NMR (400 MHz, CDCl_3_) and ^13^C NMR (101 MHz, CDCl_3_) of 4-(methylamino)isophthal-aldehyde (3w)**


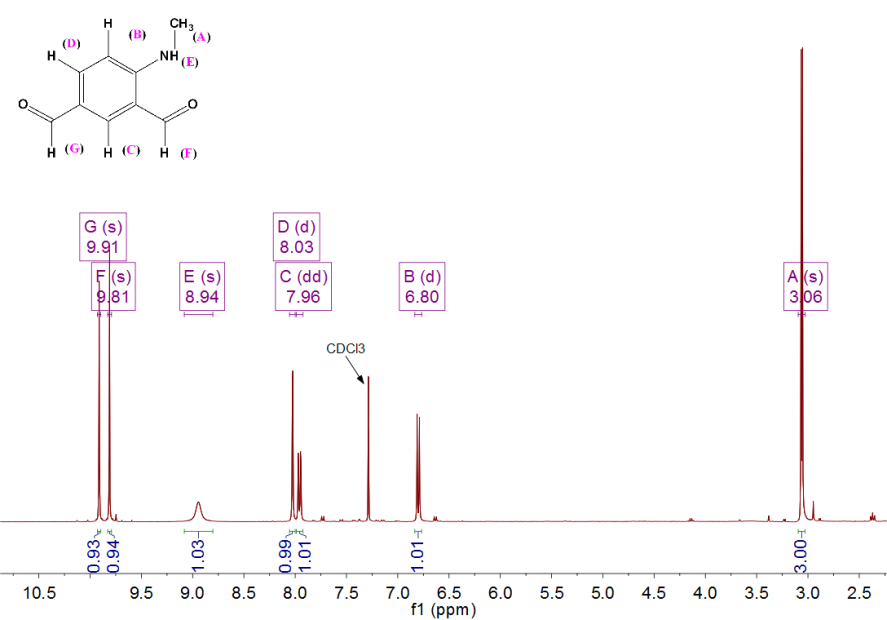


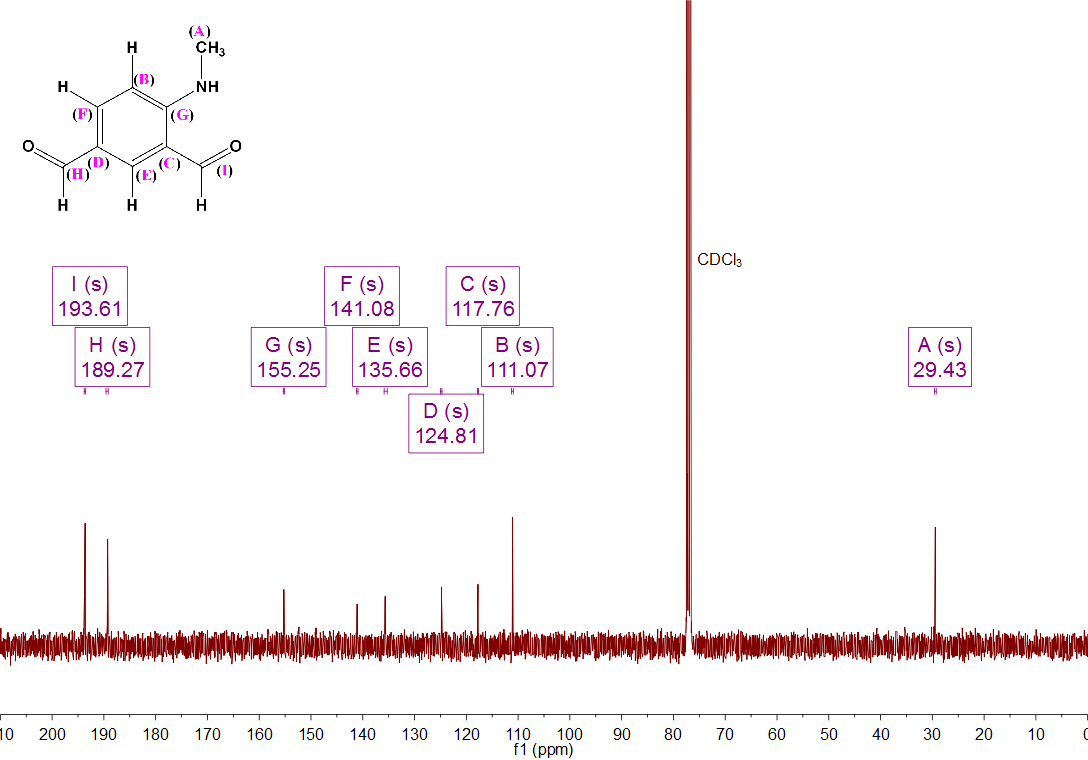


**^1^H NMR (400 MHz, CDCl_3_) and ^13^C NMR (101 MHz, CDCl_3_) of 2-amino-5-benzoylbenz-aldehyde (3x)**


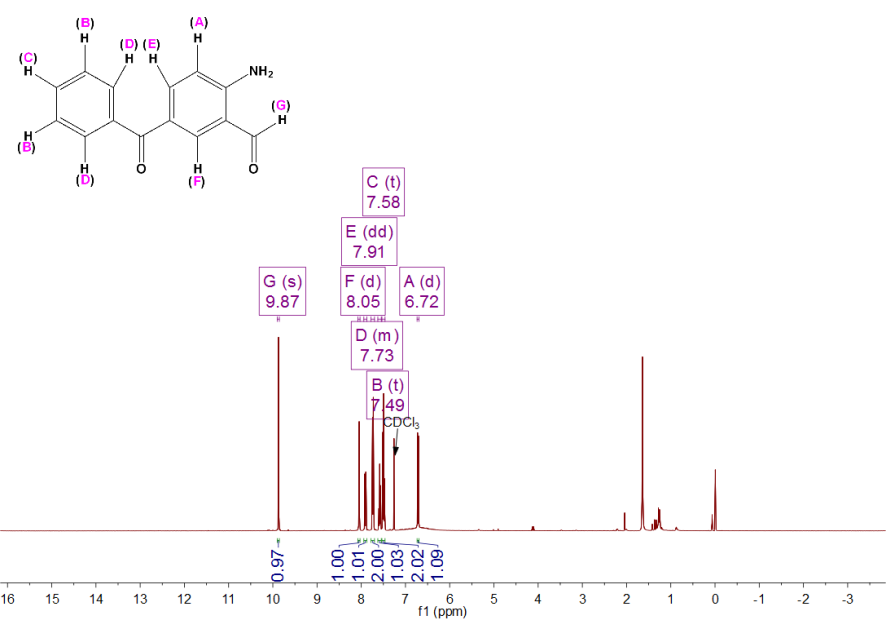

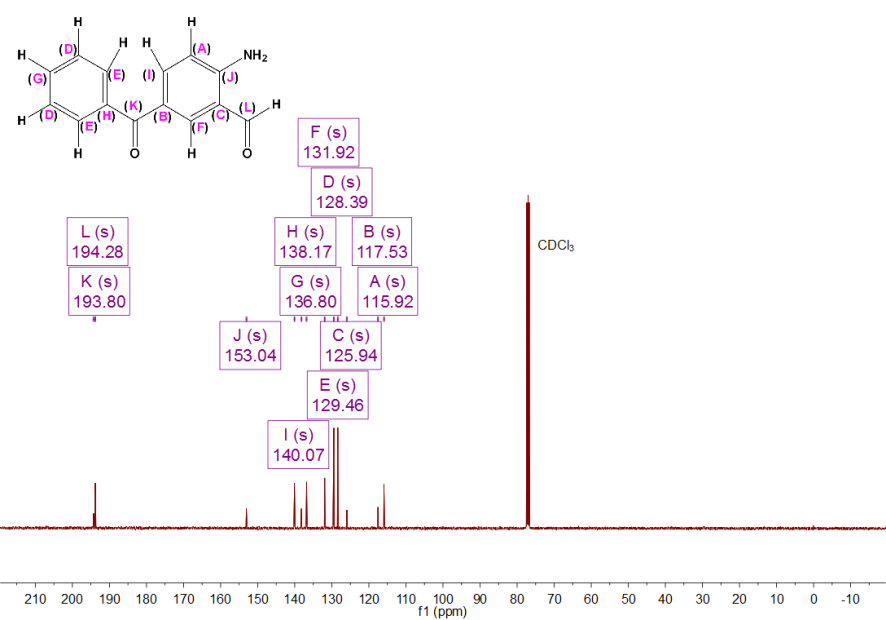


**6. IR spectrum of compound 3a**





**Figure S4** IR spectrum of compound **3a**, recorded on Bruker Vertex 70 without the support of KBr.
